# Global, regional, and national burden of tobacco-related neurological disorders from 1990 to 2021: Trends and future projections

**DOI:** 10.18332/tid/201966

**Published:** 2025-03-12

**Authors:** Chenyang Zhang, Zhihan An, Jixuan Jiang, Jingyi Ge, Wanqiong Huang, Jialin Pei, Yiyao Liu, Jiayu Yao, Zirui Guo, Xuanxi Liu, Yanhui Cui

**Affiliations:** 1 Xiangya School of Medicine, Central South University, Changsha, China; 2 Xiangya School of Basic Medical Sciences, Central South University, Changsha, China; 3 Department of Anatomy and Neurobiology, Xiangya School of Basic Medical Sciences, Central South University, Changsha, China

**Keywords:** disability-adjusted life years, neurological disorders, tobacco, trends

## Abstract

**INTRODUCTION:**

Tobacco use is as a major public health concern around the world, adversely impacting quality of life. Our study aims to analyze the trends in the burden of tobacco-related neurological disorders (ND) at global, regional, and national levels from 1990 to 2021, as well as potential future trends.

**METHODS:**

We performed a secondary dataset analysis for the assessment of mortality and disability-adjusted life years (DALYs) using data from the Global Burden of Diseases, Injuries, and Risk Factors Study (GBD) 2021, to explore the burden of tobacco-related ND. We also analyzed the associations between the burden of ND and factors such as age, gender, and the Sociodemographic Index (SDI).

**RESULTS:**

In 2021, the age-standardized mortality rate (ASMR) and age-standardized DALYs rate (ASDR) for neurological disorders were 0.50 per 100000 persons (95% UI: -0.15–1.98) and 11.25 per 100000 persons (95% UI: 1.36–34.36), respectively. Alzheimer’s disease and other dementias (ADOD) had a particularly significant impact on the burden of ND. However, the ASDR for Parkinson’s disease was -8.38 per 100000 persons (95% UI: -10.72 – -6.20). The burden of disease was greater in men and older people, with substantial regional variation. While aging and population growth might contribute to the increase in DALYs for tobacco-related ND, epidemiological changes have the potential to reduce the burden. From 2022 to 2050, the BAPC model predicted a decline in the age-standardized rate (ASR) of DALYs and mortality for tobacco-related ND, globally.

**CONCLUSIONS:**

Tobacco use increased the burden of ND such as ADOD and multiple sclerosis (MS), while reducing the burden of Parkinson’s disease (PD). The burden of disease was disproportionately higher among older individuals and males, with significant disparities across nations and regions. Over the past 32 years, the burden of these diseases has gradually decreased, and this trend is expected to continue from 2022 to 2050.

## INTRODUCTION

According to the World Health Organization’s report on trends in the prevalence of tobacco use from 2000 to 2030, the global population exposed to tobacco has been decreasing, primarily due to the implementation of national policies and increased public awareness of prevention. However, the addictive nature of tobacco and its tendency for recurrence has contributed to the persistently high prevalence of tobacco exposure^1,2^, posing as a major public health concern. Tobacco exposure is a key driver of various diseases, including neurological, respiratory, and circulatory diseases^3^, thereby significantly contributing to the global disease burden^4,5^. Tobacco use can impair cognition, memory, social-emotional, and other neurological functions^6,7^, by altering brain structure and influencing neurological development^8,9^, increasing the risk of multiple neurological diseases by more than 10%^4^. However, no studies have thoroughly analyzed the trend and imbalance in the distribution of tobacco-related ND. In this study, we explored the disease burden of tobacco-related ND, including ADOD, MS, and PD from 1990 to 2021, based on the GBD database. We focused on the trend of DALYs and the imbalance in their distribution, aiming to provide new insights for medical practitioners and public health policymakers worldwide.

## METHODS

### Overview

In this secondary dataset analysis, we analyzed mortality and DALYs for tobacco-related ND (including ADOD, MS, and PD) from 1990 to 2021. The GBD database does not provide results for tobacco-related neurological diseases caused by secondhand smoke or chewing tobacco. So in our research, the burden of tobacco-related neurological diseases is primarily caused by active smoking. The GBD 2021 database provided age-structured data for these diseases, focusing on individuals aged ≥40 years for ADOD, and ≥30 years for both MS and PD. Our study comprised several components: 1) the distribution of the disease burden across different countries over various years; 2) the distribution of the disease burden in different ages and genders; 3) trends in disease burden across regions with varying SDI levels; 4) the impact of socio-economic development on health; and 5) projection of disease burden trends from 2022 to 2050.

### Definitions

*Disease definitions*

According to WHO, neurological disorders are diseases of the central and peripheral nervous system, that cause significant damage to cognitive functioning^10^. The ICD-10 codes for ND are as follows: F00-F02.0, F02.2-F02.3, F02.8-F03.9, G10-G13.8, G20-G20.9, G23-G24, G24.1-G25.0, G25.2-G25.3, G25.5, G25.8-G26.0, G30-G31.1, G31.8-G31.9, G35-G37.9, G40-G41.9, G61-G61.9, G70-G71.1, G71.3-G72, G72.2-G73.7, G90-G90.9, G95-G95.9, and M33-M33.9. When exploring the burden of tobacco-related ND, in GBD 2021, it includes the burden in ADOD, MS, PD. ADOD is one of the neurodegenerative conditions that primarily affect older adults, inhibiting cognitive capacity, mobility, independence^11^, The corresponding ICD-10 codes are F00-F02.0, F02.8-F03.9, G30-G31.1 and G31.8-G31.9. MS is a neuroinflammatory disease of the CNS that causes demyelination and neuronal injury^12^, with the ICD-10 codes G35-G35.9. PD is defined as a core clinical motor syndrome accompanied by substantia nigra pars compacta neurodegeneration and synuclein deposition^13^, with the ICD-10 codes F02.3 and G20-G20.9.

*Age-standardized DALYs rate*

To calculate DALYs for tobacco-related ND, the GBD database combined years of life lost (YLL) and years lived with disability (YLD) to evaluate the impact of tobacco on quality of life and premature death. After adjusting for the population age distribution, the ASDR reflects the overall health burden of a specific disease, providing a standardized measure for comparisons across populations with varying age structures.

*Sociodemographic index*

The SDI combines fertility rates, education level, and per capita income to assess the socioeconomic development levels of countries or regions. SDI typically ranges from 0 to 1, with higher values reflecting greater levels of socioeconomic development^14^. Beyond serving as a tool for measuring national development, SDI also helps to uncover the underlying causes of health inequalities between different regions. GBD 2021 divided different countries and regions into five main categories: low SDI (0.47), low-middle SDI (0.47–0.62), middle SDI (0.63–0.71), high-middle SDI (0.72–0.81), and high SDI (0.82–1.00).

### Data sources

To explore the global, regional, and national burden of tobacco-related ND, we utilized data from the GBD 2021 website (https://www.healthdata.org). This comprehensive database includes the attributable burden of 88 major risk factors from 1990 to 2021, covering seven super regions, 21 regions, 204 countries and territories, and 811 subnational areas. The data in our study mainly included: 1) DALYs and mortality attributable to tobacco-related ND across different sexes and age groups from 1990 to 2021 at global, regional, and national levels; 2) population data for various age groups from 1990 to 2021 at global, regional, and national levels; 3) SDI data from 1990 to 2021 for 204 countries and regions; 4) population projections from 2018 to 2100 in the GBD database; and 5) standard age-structured data from the GBD database.

### Statistical analysis

We analyzed the burden of ND, ADOD, MS, and PD from 1990 to 2021 by the distribution of DALYs across 21 regions and 204 countries and territories. Bar charts and global maps visually highlight the inequalities in the distribution of the disease burden. Joinpoint 5.1.0 was employed to obtain annual percent change (APC), average annual percent change (AAPC), and 95% uncertain intervals (UI) for ND, primarily to evaluate ASDR trends^15^; 95% UI indicates that, in repeated sampling, 95% of the uncertain intervals derived from the samples will contain the true value of the population parameter. Previous studies have discovered that tobacco was a protective factor for PD^4,16,17^. In the GBD database, tobacco was associated with a reduced burden of PD^18^, with negative values for YLL, YLD, and DALYs across all age groups. The APC and AAPC for PD were calculated manually by fitting curves for different intervals given by Joinpoint, as well as through logarithmic transformation in Joinpoint after converting negative values to positive ones. The results of both methods were comparable. It is clarified that the APC and AAPC for PD presented in our follow-up results were obtained after fitting with Joinpoint 5.1.0 with negative value transformation. Furthermore, the positive and negative values of APC and AAPC were opposed to the actual trends in PD.

Decomposition analysis was used to differentiate the contributions of aging, population growth, and epidemiological changes to DALYs for tobacco-related ND in different SDI regions. Decomposition analysis facilitated a detailed assessment of the independent contributions of each factor to the overall changes in disease burden. This approach allowed us to break down the overall changes in disease burden into key contributing factors, providing clearer insights into how demographic and epidemiological shifts have influenced trends over time.

Health inequality analyses using the slope index of inequality (SII) and the concentration index (CI) revealed disparities in health status distribution across SDI regions. The SII measures absolute inequality in a health indicator between the most and least advantaged subgroups within a population, while the CI quantifies relative health inequality, emphasizing the degree of bias in the distribution of health resources.

To assess the relationship between the burden and SDI levels, we employed frontier analysis. Frontier analysis is a methodological approach used in epidemiology and public health research to evaluate the efficiency and performance of health interventions, healthcare systems, or population health outcomes. It identifies the frontier of optimal performance by comparing observed data with the best possible outcomes achieved under similar conditions. Using frontier analysis, we identified the potential for different countries to improve their disease burden including: 1) 15 countries with the greatest potential for reducing the disease burden; 2) 5 countries with low SDI but have better control of the disease burden; and 3) 5 countries with high SDI but still have potential to improve the disease burden.

INLA (version 23.05.30) and BAPC (version 0.0.36) models were employed to forecast future trends. All analyses were conducted by R version 4.3.3, with p<0.05 considered statistically significant.

## RESULTS

### DALYs burden of ND

Worldwide, the DALYs for tobacco-related ND increased significantly. The DALYs for ADOD increased, and for PD decreased. However, the DALYs for MS remained stable (Supplementary file Figure 1). The crude DALYs rate for ND rose from 10.10 (95% UI: 1.89–29.33) in 1990 to 11.74 (95% UI: 1.32–36.24) in 2021 (Table 1). Similarly, the crude DALYs rate for ADOD increased from 14.90 (95% UI: 6.46–34.49) in 1990 to 19.43 (95% UI: 8.40–44.31) in 2021. However, the crude DALYs rates for MS and PD decreased, from 1.98 (95% UI: 1.65–2.35) to 1.41 (95% UI: 1.15–1.70) for MS, and from -6.78 (95% UI: -8.70 – -5.09) to -9.10 (95% UI: -11.59 – -6.72) for PD (Supplementary file Table 1). Despite these trends, the ASDR for ND has been declining overall, with a greater burden observed in males compared to females (Supplementary file: Figure 1 and Table 1).

**Table 1:** Global and regional DALYs of tobacco-related neurological disorders in 1990 and 2021, and AAPC of DALYs from 1990 to 2021

	*1990*		*2021*		*1990–2021*	
	*All-ages DALYs rate* *(95% UI)*	*ASDR* *(95% UI)*	*All-ages DALYs rate* *(95% UI)*	*ASDR* *(95% UI)*	*AAPC* *(95% UI)*	*p*
**Global**	10.10 (1.89–29.33)	16.07 (3.03–45.62)	11.74 (1.32–36.24)	11.25 (1.36–34.36)	-1.17 (-1.22 – -1.12)	<0.001
**Sex**						
Female	9.86 (4.24–22.60)	13.40 (5.58–31.13)	10.03 (4.01–23.17)	8.45 (3.41–19.43)	-1.49 (-1.51 – -1.46)	<0.001
Male	10.34 (-0.47–36.53)	20.80 (0.34–68.96)	13.44 (-1.50–50.05)	15.50 (-1.00–54.96)	-0.98 (-1.07 – -0.88)	<0.001
**SDI quintile**						
High	31.47 (11.70–76.25)	25.87 (10.00–61.19)	32.55 (9.57–81.61)	16.46 (5.54–39.76)	-1.45 (-1.47 – -1.43)	<0.001
High-middle	11.26 (0.87–35.31)	14.05 (1.34–42.65)	18.56 (1.05–59.80)	12.50 (0.92–39.38)	-0.46 (-0.58 – -0.35)	<0.001
Low	1.57 (-0.06–5.51)	5.71 (0.26–18.36)	1.47 (-0.05–5.16)	4.78 (0.18–15.83)	-0.57 (-0.71 – -0.44)	<0.001
Low-middle	3.17 (-0.08–11.17)	9.12 (0.45–29.62)	4.07 (-0.29–14.57)	7.10 (-0.07–24.88)	-0.78 (-0.90 – -0.67)	<0.001
Middle	5.62 (-0.40–20.09)	13.36 (0.24–44.85)	9.52 (-0.53–33.46)	10.18 (-0.01–33.97)	-0.94 (-1.12 – -0.76)	<0.001
**GBD region**						
Andean Latin America	1.17 (-0.66–5.36)	2.67 (-1.23–11.71)	1.29 (-1.64–7.54)	1.56 (-1.87–8.92)	-1.76 (-2.30 – -1.22)	<0.001
Australasia	17.81 (5.73–44.36)	15.94 (5.44–39.37)	19.19 (6.03–47.45)	11.07 (3.97–26.74)	-1.17 (-1.23 – -1.11)	<0.001
Caribbean	6.28 (1.07–18.48)	9.48 (1.68–27.47)	7.76 (0.85–23.32)	6.69 (0.69–20.20)	-1.14 (-1.28 – -1.00)	<0.001
Central Asia	4.27 (0.87–12.18)	6.73 (1.25–19.56)	5.70 (1.17–16.70)	7.43 (1.27–22.27)	0.32 (0.21–0.43)	<0.001
Central Europe	21.76 (9.75–48.34)	19.65 (8.91–43.36)	21.95 (5.51–58.18)	12.42 (4.14–31.04)	-1.49 (-1.57 – -1.40)	<0.001
Central Latin America	3.18 (0.37–9.90)	7.76 (1.05–23.52)	3.80 (0.38–11.58)	3.99 (0.39–12.05)	-2.10 (-2.22 – -1.98)	<0.001
Central Sub-Saharan Africa	0.96 (-0.01–3.45)	3.62 (0.22–11.92)	0.98 (0.02–3.22)	3.29 (0.28–10.41)	-0.31 (-0.35 – -0.27)	<0.001
East Asia	7.46 (-2.40–30.32)	17.33 (-1.70–59.70)	18.68 (-2.55–70.77)	14.61 (-1.08–51.18)	-0.74 (-0.99 – -0.49)	<0.001
Eastern Europe	9.80 (3.00–25.70)	8.83 (2.91–22.26)	14.18 (2.90–40.08)	8.69 (2.25–23.38)	-0.04 (-0.25–0.18)	0.742
Eastern Sub-Saharan Africa	1.50 (0.07–4.86)	6.42 (0.93–18.67)	1.26 (0.09–3.90)	4.67 (0.59–13.50)	-1.02 (-1.08 – -0.96)	<0.001
High-income Asia Pacific	17.77 (2.92–52.16)	17.27 (3.20–49.93)	34.91 (8.80–92.01)	11.38 (2.74–30.67)	-1.33 (-1.41 – -1.24)	<0.001
High-income North America	45.62 (20.05– 104.61)	36.82 (17.04–82.24)	39.07 (13.60–93.03)	22.34 (8.80–50.27)	-1.61 (-1.64 – -1.57)	<0.001
North Africa and Middle East	6.26 (1.30–17.46)	16.45 (3.06–46.91)	7.85 (1.87–21.60)	12.99 (2.76–36.41)	-0.76 (-0.80 – -0.72)	<0.001
Oceania	1.95 (-0.55–7.76)	6.41 (-0.73–22.60)	2.27 (-0.34–8.36)	5.58 (-0.19–19.33)	-0.45 (-0.50 – -0.40)	<0.001
South Asia	2.29 (-0.51–8.98)	7.31 (-0.39–25.51)	3.35 (-0.57–12.92)	5.73 (-0.44–20.87)	-0.77 (-0.98 – -0.56)	<0.001
South-East Asia	4.58 (0.03–15.55)	11.78 (0.86–37.14)	6.11 (-0.74–22.99)	8.23 (-0.48–29.39)	-1.15 (-1.19 – -1.11)	<0.001
Southern Latin America	7.69 (1.55–21.35)	8.60 (1.75–23.41)	8.93 (1.36–26.18)	6.98 (1.22–20.14)	-0.69 (-0.79 – -0.60)	<0.001
Southern SubSaharan Africa	5.85 (1.54–15.44)	14.21 (3.99–36.79)	3.25 (0.46–10.21)	5.57 (0.84–17.01)	-2.98 (-3.04 – -2.91)	<0.001
Tropical Latin America	13.27 (3.49–35.90)	27.20 (6.94–73.48)	18.03 (4.94–47.96)	16.36 (4.46–43.37)	-1.64 (-1.68 – -1.59)	<0.001
Western Europe	32.58 (10.06–82.50)	22.99 (8.35–55.24)	34.20 (10.09–86.02)	15.95 (5.89–37.29)	-1.18 (-1.22 – -1.14)	<0.001
Western Sub-Saharan Africa	0.67 (-0.05–2.39)	2.10 (0.00–6.99)	0.44 (-0.09–1.78)	1.48 (-0.24–5.57)	-1.13 (-1.21 – -1.04)	<0.001

ASDR: age-standardized DALYs rate. AAPC: average annual percent change.

### Mortality burden of ND

The crude mortality for tobacco-related ND increased from 0.34 (95%UI: -0.14–1.40) in 1990 to 0.49 (95% UI: -0.17–2.01) in 2021. Similarly, the crude mortality for ADOD increased from 0.60 (95% UI: 0.14–1.67) in 1990 to 0.85 (95% UI: 0.20–2.34) in 2021. In contrast, the crude mortality for MS and PD decreased from 0.03 (95% UI: 0.03– 0.04) and -0.30 (95%UI: -0.39 – -0.22) to 0.02 (95% UI: 0.02–0.03) and -0.38 (95% UI: -0.50 – -0.28). The AAPCs of ASMR for ND, ADOD, MS, and PD were -0.98 (95% UI: -1.06 – -0.90), -0.84 (95% UI: -0.87 – -0.82), -2.01 (95% UI: -2.24 – -1.78), and -0.73 (95% UI: -0.84 – -0.61), respectively (Supplementary file Table 2).

### Epidemiological trends of ND

Joinpoint regression analysis was employed to analyze the trend of ASDR from 1990 to 2021, calculating the APC and AAPC (Supplementary file: Figure 2 and Table 3). The AAPCs in ASDR for ND, ADOD, MS, and PD were -1.17 (95% UI: -1.22 – -1.12), -0.79 (95% UI: -0.82 – -0.76), -1.96 (95% UI: -2.13 – -1.79), and -0.43 (95% UI: -0.51 – -0.35), respectively. Our findings indicated that the burden of ND has been decreasing. However, the protective effect of tobacco on PD has also declined.

### Age and SDI distribution of ASDR

Subsequently, we analyzed the distribution of DALYs for ND across age groups and then stratified the data into five-year age intervals with bar graphs. The global DALYs rate is displayed in the bottom transparency layer, with the DALYs rate across different age groups on the top (Supplementary file Figure 3). Our findings indicated that the DALYs rate for ADOD increased with age in 1990, with rates in high SDI regions significantly exceeding global levels and decreasing in lower SDI regions. By 2021, this age-related trend remained consistent. However, the DALYs rate tended to rise and subsequently fall with decreasing SDI levels (Supplementary file Figure 4). The age structure of the DALYs rate for MS was spindle-shaped and decreased with decreasing SDI levels in both 1990 and 2021 (Supplementary file Figure 5). The DALYs rate for PD followed a similar trajectory as for ADOD (Supplementary file Figure 6).

### Regional differences in ASDR

**Figure 1:**
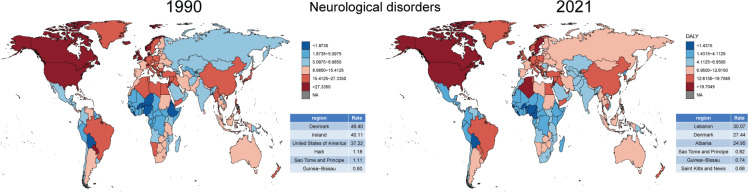
Comparison of ASDR for tobacco-related neurological disorders in 1990 and 2021 from 204 countries and territories worldwide

**Figure 2:**
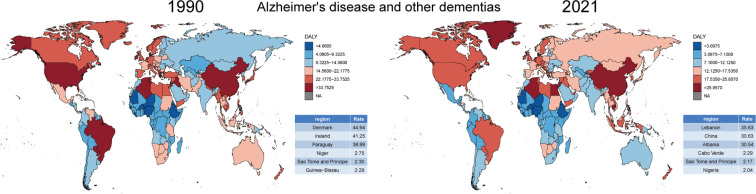
Comparison of ASDR for tobacco-related Alzheimer’s disease and other dementias in 1990 and 2021 from 204 countries and territories worldwide

**Figure 3:**
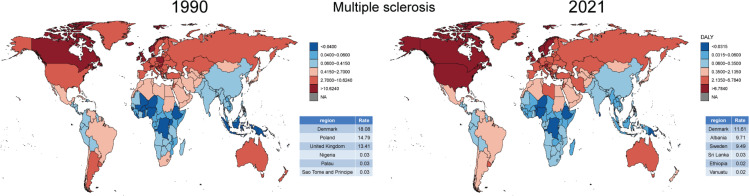
Comparison of ASDR for tobacco-related multiple sclerosis in 1990 and 2021 from 204 countries and territories worldwide

**Figure 4:**
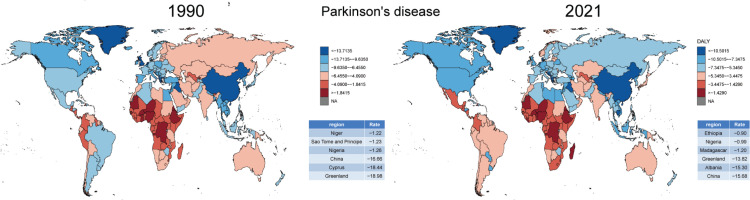
Comparison of ASDR for tobacco-related Parkinson’s disease in 1990 and 2021 from 204 countries and territories worldwide

There were significant regional differences in the ASDR and its trends for tobacco-related ND. To better understand the ASDR across 204 countries and regions, we represented the distribution of ASDR in 1990 and 2021 (Figure 1). The data were divided into six categories based on the 5th, 25th, 50th, 75th, and 95th percentiles, highlighting the three regions with the highest and lowest ASDR. In 1990, Denmark, Ireland, and the United States had the highest disease burden, while Haiti, São Tomé and Príncipe, and Guinea-Bissau had the lowest. However, by 2021, the burden shifted, with Lebanon, Denmark, and Albania showing the highest ASDR, and São Tomé and Príncipe, Guinea-Bissau, and Saint Kitts and Nevis the lowest. In ADOD and MS, there was a significant imbalance in the distribution of disease burden (Figures 2 and 3). However, tobacco use could reduce the disease burden of PD (Figure 4).

To assess trends in ND among 204 countries and regions, we created an AAPC map for ASDR (Supplementary file Figure 7). Between 1990 and 2021, Seychelles, Kyrgyzstan, and Mali exhibited the most rapid upward trends in tobacco-related ND, while Cape Verde, Ecuador, and South Africa showed the most significant decreases.

### Decomposition analysis of DALYs

A decomposition analysis was conducted to evaluate the effects of population growth, aging, and epidemiological change trends in DALYs from 1990 to 2021 (Supplementary file: Figure 8 and Table 4). Our findings revealed that epidemiological changes reduced the burden of tobacco-related ND, including ADOD and MS, whereas population growth and aging could greatly increase the burden of the above diseases, as opposed to Parkinson’s disease caused by tobacco. Among the factors, aging had the most substantial impact on these diseases.

### Health inequality analysis

We then conducted a health inequality analysis (Supplementary file Figure 9) to assess the improvement in the unbalanced worldwide distribution of disease burden. The SII for tobacco-related ND decreased from 11.95 (95% UI: 8.86–15.05) in 1990 to 9.19 (95% UI: 7.11–11.27) in 2021, and the CI increased from -0.22 (95% UI: -0.30 – -0.15) in 1990 to -0.21 (95% UI: -0.29 – -0.13) in 2021. The SII for ADOD decreased from 13.20 (95% UI: 9.54–16.86) in 1990 to 8.22 (95% UI: 5.72–10.73) in 2021, and the CI increased from -0.14 (95% UI: -0.21 – -0.06) to -0.12 (95% UI: -0.20 – -0.04). The SII for MS decreased from 3.97 (95% UI: 3.19–4.74) in 1990 to 3.31 (95% UI: 2.77–3.84) in 2021, and the CI increased from -0.60 (95% UI: -0.67 – -0.53) in 1990 to -0.56 in 2021 (95% UI: -0.63 – -0.48). The SII for PD decreased from -5.58 (95% UI: -6.96 – -4.20) in 1990 to -3.31 (95% UI: -4.30 – -2.32) in 2021, and the CI decreased from -0.13 (95% UI: -0.22 – -0.06) in 1990 to -0.11 (95% UI: -0.19 – -0.03). This indicated that the disease burden was concentrated in regions with high SDI but a small percentage of their population. From 1990 to 2021, inequality in the distribution of the disease burden has improved.

### Correlation between the distribution of the burden of disease and the SDI

Next, we explored the relationship between ASDR and SDI, and the expected ASDR level based on SDI for tobacco-related ND in global, 21 GBD regions, 204 nations and regions (Supplementary file Figure 10). The results revealed a positive correlation between SDI and ND (p<0.001), indicating that countries with higher SDI levels tend to have a greater disease burden. However, a negative correlation was observed for PD (p<0.001).

Our analysis indicated that the disease burden in regions such as Eastern Europe and East Asia initially increased with rising SDI before subsequently declining. In Central Asia, the disease burden first decreased and then declined further as SDI increased. In other regions, the burden of tobacco-related neurological diseases decreased with increasing SDI.

With changes in SDI levels, it is noteworthy that the burden of disease for tobacco-related ND, ADOD, and MS decreased globally and across most of the 21 GBD regions over time, as well as the protective effect of PD decreased. These findings are consistent with the results obtained from our AAPC joinpoint regression analysis (Supplementary file Table 1).

### Frontier analysis

In the comprehensive frontier analysis based on SDI and ASDR of tobacco-related ND spanning from 1990 to 2021 across 204 countries and territories, distinct trends emerged. Countries with high SDI are expected to have a lower disease burden, but many countries show the opposite trend. When comparing the disease burden between 1990 and 2021, many countries have seen a decrease in ASDR. This trend suggested that the global burden of tobacco-related neurological diseases had decreased over time. (Supplementary file: Figure 11 and Table 5). The 15 countries furthest from the frontier (country names highlighted in black) demonstrated the greatest potential for improvement. Additionally, we identified the 5 countries closest to the expected level with low SDI levels and the 5 countries farthest from the expected level with high SDI levels: country names are marked in blue and red, respectively, where blue indicates countries with effective disease burden control despite relatively low SDI levels, and red signifies countries with less satisfactory outcomes despite high SDI levels.

### BAPC projections of the burden for ND from 2022 to 2050

**Figure 5:**
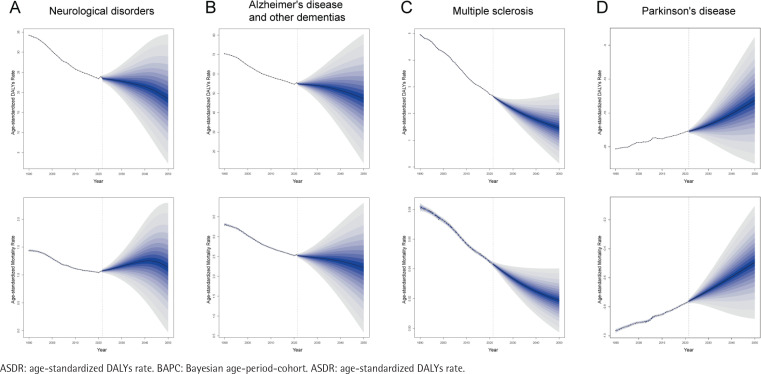
Projections of ASDR from 2022 to 2050 based on the BAPC model: A) ASDR for tobacco-related neurological disorders; B) ASDR for tobacco-related Alzheimer’s disease and other dementias; C) ASDR for tobacco-related multiple sclerosis; and D) ASDR for tobacco-related Parkinson’s disease

Using standard age-structured data from the GBD database and population projections from 2018 to 2100, BAPC and INLA were used to forecast the ASDR and ASMR for tobacco-related ND from 2022 to 2050 (Figure 5). The line preceding the dividing marker represented actual data from 1990 to 2021, while the line following the marker represented projected data from 2022 to 2050, including uncertainty intervals. The ASDR for ND, ADOD, and MS would decrease over the next 29 years, whereas the ASDR for PD was expected to increase. The ASMR for ND was anticipated to rise initially before declining. Overall, the burden of tobacco-related ND would improve in the future.

## DISCUSSION

Smoking is widely recognized as a major risk factor contributing to the global disease burden. Although smoking prevalence has decreased over the past 30 years, the total number of smokers worldwide has continued to rise due to population growth^2^. According to GBD 2021, smoking is the fourth leading contributor to the global burden of ND. Tobacco-related ND can significantly impact patients’ behavior and consciousness^6^, thereby reducing their quality of life. Therefore, it is essential to conduct in-depth research on the trend in the burden of tobacco-related ND and to implement effective public health strategies to reduce the burden.

Based on the GBD database, we carried out a comprehensive analysis of the burden of tobacco-related ND from 1990 to 2021. We also analyzed the distribution and the influence on gender, age, and SDI. Additionally, we focused on cross-national disparities and future disease burdens. To present the findings more intuitively, we primarily used DALYs to measure the disease burden, providing new insights for the epidemiological study of tobacco-related diseases.

We discovered that tobacco use could greatly increase the burden of ND, including ADOD and MS. However, we also noticed that tobacco reduced the burden of Parkinson’s disease, which is consistent with current studies^4,19^. The research found that tobacco might reduce α-synuclein levels and prevent damage to dopamine neurons^20^. Carbon monoxide in tobacco could reduce oxidative stress and inhibit cell death^21,22^. Parkinson’s disease patients might have a lower sensitivity to nicotine, so they are more likely to quit smoking^23^. However, the potential benefits of tobacco could not overcome its toxic effects on multiple systems throughout the body.

The trends of ASDR and crude DALYs rate for tobacco-related ND were completely opposite. This suggested that, with the strengthening of public health measures, improvements in treatment, and increased health awareness, the burden of ND was decreasing^24^. However, population growth, aging, and increased life expectancy might increase the absolute burden^25^. Thus, health strategies should be adjusted accordingly with changes in population structure.

Our findings indicated that effective public health policies could significantly reduce the disease burden and improve the inequality in its distribution. South Africa achieved notable success by implementing strict tobacco control measures^26^. Population growth and aging could increase the burden of ND^27,28^, with aging being the most critical factor. Aging leads to irreversible changes in brain structure and function, including reduced neuroplasticity and the accumulation of neurotoxic proteins^27,28^, which significantly increased the incidence of diseases within the elderly population.

The crude DALYs rate and ASDR in ND were significantly higher in males than females, as well as in AD and MS. However, current studies suggest that females were at a higher risk of developing AD and MS^29,30^. This trend might depend on the greater prevalence of smoking among males^31^. According to WHO projections, the gender gap is expected to increase.

The population’s risk of developing ND increased with age^32^, which was consistent with our findings. However, MS tends to occur at an earlier age than AD and PD. In our results, the DALY rate for MS exhibited a spindle-shaped distribution.

In high SDI regions, the ASDR for MS showed a declining trend, possibly due to increased health awareness among residents or the implementation of effective public health policies by local governments^33^. In contrast, the initially rising and then declining trend in high-middle and middle SDI regions might suggest that tobacco use remains prevalent in the early stages due to cultural factors and insufficient public health policies. Although the proportion of tobacco use decreased with the improvement of policies and increased health awareness, the cumulative effect of early exposure might result in the trend of the disease burden, potentially reflecting a lag between the implementation of public health measures and their impact on disease burden. Socioeconomic factors, limited healthcare resources, and high tobacco use might all contribute to the increasing trend in ASDR in low-middle and low SDI regions. Our study suggested that the ASDR trends for tobacco-related MS across different SDI regions demonstrated the complex interplay between demographic factors, public health interventions, and attitudes toward smoking.

By projecting trends of tobacco-related ND from 2022 to 2050, we found that ASDR would decline over the next 29 years. Although the ASMR for ND has shown an upward trend in recent years, it is expected to decrease in the future. These trends emphasize the importance of implementing effective public health strategies.

### Limitations

Our study used the latest data from the GBD 2021 to analyze the burden of tobacco-related ND, but it still has limitations. First of all, some data in the GBD database were not directly collected, which might lead to discrepancies from actual conditions. In the GBD database, data collection is subject to several limitations, including unmeasured or unknown confounders, measurement error in confounders, and limitations in statistical models, all of which may lead to residual confounding and potentially impact our conclusions. In our data analysis, methods such as Joinpoint regression and future trend predictions rely on model-fitted results generated by statistical software or R packages, which may lead to error. Additionally, when studying the disease burden of ND attributable to tobacco, our study only focuses on smoking, as the GBD database lacks data on secondhand smoke or chewing tobacco exposure. Furthermore, the GBD database did not include data on tobacco-related ND for all age groups, limiting our ability to analyze age distribution comprehensively. Meanwhile, the GBD database covers a limited range of diseases. In our analysis of tobacco-related neurological disorders, we can only focus on ADOD, MS, and PD, further research is needed to explore the burden on other diseases.

## CONCLUSIONS

Although the burden of tobacco-related ND has declined over the past 32 years, it remains a significant concern due to global population growth. There are significant differences in the burden of tobacco-related ND across regions, genders, and SDI levels. Implementing effective public health measures to control tobacco use remains crucial for reducing the burden.

## Supplementary Material



## Data Availability

The data supporting this research are available from the authors on reasonable request.
