# Supplementary material for: Global, regional, and national burden of tobacco-related neurological disorders from 1990 to 2021: Trends and future projections
Source: Tob Induc Dis. 2025 Mar 12;23:10.18332/tid/201966. doi: 10.18332/tid/201966 (PMC11897908; doi:10.18332/tid/201966)
Supplement: Supplementary file 1 [file TID-23-27-s1.pdf]

Supplementary Table 1. Global and regional DALYs of tobacco-related three Neurological Disorders in 1990 and 2021, and AAPC of DALYs from 1990 to 2021

		1990		2021		1990-2021	
**Disease**	**Location**	**All-ages DALYs rate**	**ASDR (95% UI)**	**All-ages DALYs rate**	**ASDR (95% UI)**	**AAPC (95% UI)**	**P-value**
Alzheimer's disease and other dementias	Global	14.90 (6.46, 34.49)	23.33 (9.99, 54.46)	19.43 (8.40, 44.31)	18.36 (7.90, 42.07)	-0.79 (-0.82, -0.76)	<0.001
	sex						
	Female	9.91 (4.26, 22.74)	13.65 (5.80, 31.32)	10.76 (4.71, 24.10)	9.01 (3.94, 20.16)	-1.35 (-1.38, -1.32)	<0.001
	Male	19.83 (8.48, 46.24)	37.45 (15.98, 87.69)	28.04 (11.97, 64.62)	30.56 (12.72, 71.50)	-0.67 (-0.71, -0.64)	<0.001
	SDI quintile						
	High SDI	35.32 (15.52, 81.48)	27.97 (12.27, 64.53)	40.48 (17.45, 93.30)	19.05 (8.33, 43.29)	-1.23 (-1.25, -1.21)	<0.001
	High-middle SDI	18.88 (7.95, 43.43)	22.72 (9.46, 52.39)	33.37 (14.66, 74.51)	21.96 (9.60, 49.15)	-0.13 (-0.18, -0.07)	<0.001
	Low SDI	3.04 (1.31, 7.33)	9.72 (4.09, 23.07)	2.69 (1.07, 6.50)	8.10 (3.29, 19.44)	-0.59 (-0.67, -0.51)	<0.001
	Low-middle SDI	6.20 (2.72, 14.75)	15.95 (6.82, 37.64)	8.00 (3.30, 19.09)	13.02 (5.45, 31.35)	-0.64 (-0.68, -0.60)	<0.001
	Middle SDI	11.35 (4.85,	24.14 (10.01,	19.21 (8.24,	19.38 (8.25,	-0.74 (-0.81,	<0.001

	25.91)	56.67)	44.37)	44.76)	-0.67)	
GBD region						
Andean Latin America	3.32 (1.40, 7.89)	7.05 (2.96, 16.84)	4.82 (2.05, 11.48)	5.65 (2.40, 13.40)	-0.70 (-0.76, -0.65)	<0.001
Australasia	21.76 (9.74, 49.52)	18.86 (8.37, 42.69)	22.66 (9.74, 53.55)	12.11 (5.19, 28.39)	-1.42 (-1.46, -1.39)	<0.001
Caribbean	9.82 (4.22, 22.34)	14.49 (6.29, 33.31)	12.77 (5.62, 29.51)	11.08 (4.89, 25.64)	-0.86 (-0.90, -0.81)	<0.001
Central Asia	6.29 (2.81, 14.56)	10.16 (4.53, 23.61)	8.42 (3.68, 19.76)	11.45 (5.08, 26.38)	0.40 (0.33, 0.47)	<0.001
Central Europe	21.51 (9.47, 48.92)	19.07 (8.36, 43.19)	29.45 (12.99, 67.55)	14.77 (6.44, 33.75)	-0.82 (-0.84, -0.80)	<0.001
Central Latin America	5.45 (2.43, 12.37)	12.89 (5.72, 29.41)	6.40 (2.82, 14.18)	6.74 (2.97, 14.90)	-2.07 (-2.11, -2.02)	<0.001
Central Sub-Saharan Africa	1.75 (0.71, 4.23)	5.88 (2.41, 14.34)	1.58 (0.60, 3.79)	5.05 (1.92, 12.35)	-0.50 (-0.56, -0.43)	<0.001
East Asia	17.98 (7.42, 41.73)	33.68 (13.33, 78.42)	41.54 (17.79, 95.19)	29.95 (12.67, 68.10)	-0.43 (-0.50, -0.35)	<0.001
Eastern Europe	12.67 (5.55, 29.11)	10.81 (4.76, 24.69)	20.88 (9.05, 47.29)	11.92 (5.23, 26.87)	0.32 (0.20, 0.44)	<0.001
Eastern Sub-Saharan	2.51 (1.02, 6.08)	9.54 (3.88, 22.72)	2.01 (0.78, 4.94)	6.91 (2.76, 16.31)	-1.03 (-1.09, -0.98)	<0.001

Africa						
High-income Asia Pacific	26.45 (11.23, 61.26)	24.84 (10.44, 57.20)	47.15 (19.88, 107.25)	15.81 (6.77, 36.48)	-1.45 (-1.47, -1.42)	<0.001
High-income North America	44.61 (19.72, 103.45)	34.48 (15.23, 79.44)	44.38 (19.67, 100.81)	23.32 (10.39, 52.46)	-1.26 (-1.30, -1.21)	<0.001
North Africa and Middle East	9.03 (3.91, 20.44)	23.87 (10.23, 54.52)	10.66 (4.56, 24.34)	18.22 (7.72, 41.54)	-0.87 (-0.89, -0.85)	<0.001
Oceania	4.56 (1.90, 10.67)	12.78 (5.38, 29.87)	4.75 (2.01, 10.94)	10.53 (4.46, 24.93)	-0.63 (-0.66, -0.59)	<0.001
South Asia	5.11 (2.21, 12.27)	13.94 (5.84, 32.94)	7.04 (2.87, 16.83)	10.95 (4.55, 26.31)	-0.76 (-0.84, -0.67)	<0.001
Southeast Asia	8.80 (3.93, 20.00)	20.78 (9.10, 47.13)	12.85 (5.52, 30.16)	16.17 (6.85, 37.31)	-0.81 (-0.84, -0.78)	<0.001
Southern Latin America	11.42 (5.13, 25.93)	12.59 (5.64, 28.39)	13.80 (5.99, 30.82)	10.53 (4.59, 23.53)	-0.58 (-0.62, -0.54)	<0.001
Southern Sub-Saharan Africa	7.80 (3.42, 17.54)	18.60 (8.00, 41.45)	5.11 (2.15, 11.91)	8.43 (3.50, 19.63)	-2.51 (-2.57, -2.45)	<0.001
Tropical Latin America	17.25 (7.05, 40.65)	34.90 (14.37, 82.59)	22.88 (9.96, 53.64)	20.81 (9.05, 48.62)	-1.65 (-1.70, -1.59)	<0.001
Western	40.17 (17.62,	25.78 (11.25,	42.20 (18.04,	17.23 (7.43,	-1.29 (-1.31,	<0.001

	Europe	91.37)	59.30)	96.21)	38.39)	-1.27)	
	Western Sub-Saharan Africa	1.30 (0.52, 3.09)	3.72 (1.48, 8.68)	0.93 (0.37, 2.33)	2.95 (1.15, 7.14)	-0.74 (-0.78, -0.71)	<0.001
Multiple sclerosis	Global	1.98 (1.65, 2.35)	2.36 (1.97, 2.81)	1.41 (1.15, 1.70)	1.28 (1.05, 1.55)	-1.96 (-2.13, -1.79)	<0.001
	sex						
	Female	1.86 (1.54, 2.24)	2.20 (1.82, 2.64)	1.31 (1.07, 1.61)	1.17 (0.95, 1.43)	-2.02 (-2.09, -1.94)	<0.001
	Male	2.08 (1.76, 2.47)	2.54 (2.14, 3.01)	1.50 (1.23, 1.81)	1.39 (1.14, 1.68)	-1.92 (-2.09, -1.75)	<0.001
	SDI quintile						
	High SDI	8.31 (6.92, 9.88)	7.26 (6.05, 8.64)	6.51 (5.30, 7.90)	4.60 (3.73, 5.62)	-1.47 (-1.56, -1.39)	<0.001
	High-middle SDI	2.38 (2.01, 2.82)	2.39 (2.02, 2.83)	1.90 (1.55, 2.29)	1.39 (1.13, 1.69)	-1.68 (-1.97, -1.40)	<0.001
	Low SDI	0.07 (0.05, 0.10)	0.12 (0.08, 0.17)	0.08 (0.06, 0.11)	0.13 (0.09, 0.18)	0.29 (0.27, 0.32)	<0.001
	Low-middle SDI	0.15 (0.11, 0.22)	0.23 (0.16, 0.31)	0.22 (0.16, 0.29)	0.24 (0.18, 0.33)	0.20 (0.15, 0.26)	<0.001
	Middle SDI	0.26 (0.20, 0.34)	0.35 (0.27, 0.45)	0.40 (0.32, 0.51)	0.35 (0.28, 0.43)	-0.03 (-0.11, 0.05)	0.471

GBD region						
Andean Latin America	0.11 (0.08, 0.14)	0.16 (0.13, 0.21)	0.20 (0.15, 0.26)	0.20 (0.15, 0.27)	0.62 (-0.07, 1.31)	0.078
Australasia	3.83 (3.10, 4.78)	3.51 (2.84, 4.36)	3.77 (2.88, 4.90)	2.94 (2.26, 3.84)	-0.53 (-0.82, -0.25)	<0.001
Caribbean	0.83 (0.67, 1.01)	1.04 (0.84, 1.26)	0.99 (0.78, 1.25)	0.89 (0.70, 1.11)	-0.49 (-0.66, -0.32)	<0.001
Central Asia	1.07 (0.85, 1.37)	1.43 (1.14, 1.82)	1.13 (0.85, 1.51)	1.12 (0.85, 1.51)	-0.83 (-1.17, -0.49)	<0.001
Central Europe	11.15 (9.56, 12.81)	9.75 (8.36, 11.23)	6.36 (5.25, 7.60)	4.52 (3.71, 5.42)	-2.46 (-2.56, -2.37)	<0.001
Central Latin America	0.26 (0.22, 0.31)	0.40 (0.33, 0.48)	0.43 (0.35, 0.52)	0.41 (0.33, 0.49)	0.03 (-0.18, 0.25)	0.755
Central Sub-Saharan Africa	0.03 (0.02, 0.04)	0.05 (0.03, 0.07)	0.03 (0.02, 0.05)	0.05 (0.04, 0.08)	0.51 (0.45, 0.57)	<0.001
East Asia	0.07 (0.05, 0.10)	0.08 (0.05, 0.11)	0.13 (0.09, 0.18)	0.09 (0.07, 0.13)	0.57 (0.45, 0.70)	<0.001
Eastern Europe	4.35 (3.73, 5.04)	3.87 (3.32, 4.47)	3.84 (3.19, 4.54)	2.89 (2.40, 3.42)	-0.87 (-1.49, -0.25)	0.006
Eastern Sub-Saharan Africa	0.03 (0.02, 0.04)	0.05 (0.04, 0.08)	0.03 (0.02, 0.04)	0.05 (0.03, 0.07)	-0.18 (-0.22, -0.14)	<0.001

High-income Asia Pacific	0.67 (0.50, 0.89)	0.56 (0.42, 0.75)	0.51 (0.38, 0.68)	0.35 (0.26, 0.47)	-1.50 (-1.74, -1.27)	<0.001
High-income North America	11.57 (9.38, 14.11)	10.52 (8.54, 12.81)	9.38 (7.57, 11.51)	6.92 (5.56, 8.51)	-1.34 (-1.42, -1.27)	<0.001
North Africa and Middle East	0.97 (0.70, 1.29)	1.48 (1.07, 1.96)	1.54 (1.20, 1.98)	1.56 (1.22, 2.01)	0.16 (0.12, 0.21)	<0.001
Oceania	0.03 (0.02, 0.04)	0.04 (0.02, 0.05)	0.03 (0.02, 0.05)	0.04 (0.02, 0.06)	0.05 (0.03, 0.07)	<0.001
South Asia	0.13 (0.09, 0.18)	0.18 (0.12, 0.25)	0.14 (0.10, 0.19)	0.15 (0.11, 0.21)	-0.56 (-0.59, -0.53)	<0.001
Southeast Asia	0.05 (0.04, 0.07)	0.07 (0.05, 0.10)	0.08 (0.06, 0.11)	0.07 (0.05, 0.10)	0.01 (-0.03, 0.06)	0.617
Southern Latin America	2.36 (1.94, 2.86)	2.50 (2.05, 3.02)	1.65 (1.29, 2.04)	1.43 (1.12, 1.78)	-1.71 (-1.91, -1.51)	<0.001
Southern Sub-Saharan Africa	0.33 (0.25, 0.43)	0.51 (0.38, 0.66)	0.26 (0.20, 0.34)	0.29 (0.22, 0.37)	-1.83 (-2.06, -1.60)	<0.001
Tropical Latin America	0.94 (0.73, 1.20)	1.28 (0.99, 1.63)	1.04 (0.80, 1.35)	0.89 (0.68, 1.15)	-1.21 (-1.43, -0.98)	<0.001
Western Europe	9.58 (7.99, 11.30)	7.93 (6.62, 9.41)	8.59 (6.89, 10.33)	6.04 (4.82, 7.30)	-0.90 (-1.04, -0.75)	<0.001

	Western Sub-Saharan Africa	0.03 (0.02, 0.04)	0.04 (0.03, 0.06)	0.03 (0.02, 0.04)	0.04 (0.03, 0.06)	0.15 (0.12, 0.18)	<0.001
	Global	-6.78 (-8.70, -5.09)	-9.61 (-12.33, -7.21)	-9.10 (-11.59, -6.72)	-8.38 (-10.72, -6.20)	-0.43 (-0.51, -0.35)	<0.001
	sex						
	Female	-1.91 (-2.44, -1.46)	-2.45 (-3.12, -1.86)	-2.04 (-2.66, -1.50)	-1.72 (-2.25, -1.27)	-1.13 (-1.21, -1.04)	<0.001
	Male	-11.57 (-14.86, -8.59)	-19.18 (-24.67, -14.24)	-16.10 (-20.58, -11.86)	-16.46 (-21.16, -12.10)	-0.49 (-0.57, -0.41)	<0.001
Parkinson's disease	SDI quintile						
	High SDI	-12.17 (-16.09, -8.72)	-9.36 (-12.36, -6.72)	-14.44 (-19.82, -9.82)	-7.20 (-9.79, -4.95)	-0.86 (-0.94, -0.78)	<0.001
	High-middle SDI	-10.00 (-12.70, -7.52)	-11.06 (-14.05, -8.32)	-16.71 (-21.30, -12.37)	-10.86 (-13.87, -8.04)	-0.06 (-0.16, 0.05)	0.293
	Low SDI	-1.54 (-2.04, -1.11)	-4.13 (-5.47, -2.92)	-1.31 (-1.75, -0.92)	-3.45 (-4.68, -2.41)	-0.56 (-0.81, -0.30)	<0.001
	Low-middle SDI	-3.18 (-4.17, -2.29)	-7.06 (-9.31, -5.01)	-4.14 (-5.42, -3.06)	-6.16 (-8.10, -4.53)	-0.42 (-0.74, -0.10)	0.009
	Middle SDI	-5.99 (-7.57,	-11.13	-10.10	-9.54 (-12.22,	-0.50 (-0.64,	<0.001

	-4.46)	(-14.02, -8.28)	(-12.99, -7.41)	-7.03)	-0.35)	
GBD region						
Andean Latin America	-2.26 (-3.16, -1.53)	-4.54 (-6.38, -3.07)	-3.73 (-5.37, -2.34)	-4.29 (-6.24, -2.68)	-0.15 (-0.49, 0.18)	0.374
Australasia	-7.79 (-10.47, -5.47)	-6.43 (-8.65, -4.52)	-7.23 (-10.64, -4.53)	-3.98 (-5.77, -2.54)	-1.59 (-1.72, -1.46)	<0.001
Caribbean	-4.37 (-5.85, -3.04)	-6.05 (-8.13, -4.19)	-6.00 (-8.35, -4.07)	-5.28 (-7.35, -3.59)	-0.39 (-0.63, -0.15)	0.002
Central Asia	-3.10 (-3.98, -2.30)	-4.86 (-6.25, -3.57)	-3.85 (-4.89, -2.88)	-5.14 (-6.63, -3.84)	0.19 (-0.10, 0.49)	0.205
Central Europe	-10.90 (-14.18, -7.94)	-9.18 (-11.99, -6.68)	-13.86 (-18.59, -9.86)	-6.87 (-9.18, -4.93)	-0.90 (-0.95, -0.85)	<0.001
Central Latin America	-2.54 (-3.26, -1.89)	-5.53 (-7.11, -4.08)	-3.03 (-4.01, -2.14)	-3.16 (-4.20, -2.22)	-1.80 (-1.98, -1.62)	<0.001
Central Sub-Saharan Africa	-0.82 (-1.12, -0.56)	-2.31 (-3.16, -1.56)	-0.64 (-0.86, -0.42)	-1.81 (-2.45, -1.19)	-0.77 (-0.90, -0.64)	<0.001
East Asia	-10.59 (-13.54, -7.68)	-16.42 (-20.90, -11.97)	-22.99 (-29.96, -16.79)	-15.43 (-20.03, -11.27)	-0.19 (-0.40, 0.03)	0.085
Eastern	-7.23 (-9.32,	-5.85 (-7.57,	-10.55	-6.11 (-8.06,	0.19 (-0.12,	0.229

Europe	-5.30)	-4.28)	(-13.96, -7.64)	-4.45)	0.51)	
Eastern Sub-Saharan Africa	-1.05 (-1.39, -0.74)	-3.18 (-4.25, -2.23)	-0.78 (-1.07, -0.51)	-2.29 (-3.23, -1.51)	-1.03 (-1.11, -0.95)	<0.001
High-income Asia Pacific	-9.35 (-11.88, -6.95)	-8.14 (-10.33, -6.06)	-12.75 (-17.17, -9.10)	-4.78 (-6.34, -3.49)	-1.69 (-1.81, -1.56)	<0.001
High-income North America	-10.56 (-14.26, -7.29)	-8.17 (-10.99, -5.65)	-14.69 (-21.32, -9.01)	-7.90 (-11.39, -4.90)	-0.10 (-0.25, 0.06)	0.213
North Africa and Middle East	-3.74 (-4.89, -2.71)	-8.90 (-11.71, -6.38)	-4.35 (-5.67, -3.09)	-6.79 (-9.00, -4.78)	-0.86 (-0.92, -0.81)	<0.001
Oceania	-2.63 (-3.55, -1.72)	-6.40 (-8.59, -4.32)	-2.51 (-3.41, -1.69)	-4.98 (-6.82, -3.34)	-0.79 (-0.83, -0.75)	<0.001
South Asia	-2.94 (-4.04, -2.05)	-6.81 (-9.37, -4.65)	-3.82 (-5.16, -2.67)	-5.37 (-7.21, -3.73)	-0.72 (-1.08, -0.37)	<0.001
Southeast Asia	-4.28 (-5.59, -3.17)	-9.07 (-12.02, -6.77)	-6.82 (-8.74, -5.01)	-8.01 (-10.29, -5.91)	-0.41 (-0.49, -0.33)	<0.001
Southern Latin America	-6.10 (-8.31, -4.07)	-6.49 (-8.93, -4.29)	-6.53 (-9.31, -4.17)	-4.98 (-7.07, -3.21)	-0.85 (-0.96, -0.73)	<0.001
Southern Sub-Saharan Africa	-2.28 (-2.88, -1.69)	-4.91 (-6.23, -3.63)	-2.12 (-2.69, -1.60)	-3.15 (-3.96, -2.37)	-1.40 (-1.70, -1.09)	<0.001

Tropical Latin America	-4.93 (-6.57, -3.59)	-8.98 (-12.07, -6.49)	-5.89 (-8.44, -3.70)	-5.34 (-7.70, -3.33)	-1.65 (-1.77, -1.53)	<0.001
Western Europe	-17.17 (-23.02, -12.11)	-10.73 (-14.33, -7.59)	-16.60 (-23.41, -10.89)	-7.32 (-10.14, -4.93)	-1.24 (-1.35, -1.13)	<0.001
Western Sub-Saharan Africa	-0.66 (-0.88, -0.48)	-1.66 (-2.22, -1.20)	-0.52 (-0.70, -0.35)	-1.51 (-2.08, -1.00)	-0.27 (-0.33, -0.20)	<0.001

Supplementary Table 2. Global and regional Mortality of tobacco-related Neurological Disorders in 1990 and 2019, and AAPC of Mortality from 1990 to 2019

		1990		2021		1990-2021	
**Disease**	**Location**	**All-ages Mortality rate**	**ASMR (95% UI)**	**All-ages Mortality rate**	**ASMR (95% UI)**	**AAPC (95% UI)**	**P-value**
	Global	0.34 (-0.14, 1.40)	0.67 (-0.17, 2.64)	0.49 (-0.17, 2.01)	0.50 (-0.15, 1.98)	-0.98 (-1.06, -0.90)	<0.001
	sex						
	Female	0.38 (0.04, 1.14)	0.58 (0.07, 1.73)	0.45 (0.05, 1.29)	0.37 (0.04, 1.07)	-1.46 (-1.50, -1.42)	<0.001
	Male	0.30 (-0.32, 1.70)	0.87 (-0.53, 4.25)	0.54 (-0.39, 2.69)	0.72 (-0.40, 3.35)	-0.66 (-0.78, -0.54)	<0.001
Neurological disorders	SDI quintile						
	High SDI	1.12 (-0.07, 3.88)	0.95 (-0.02, 3.20)	1.40 (-0.14, 4.89)	0.60 (-0.06, 2.10)	-1.46 (-1.49, -1.44)	<0.001
	High-middle SDI	0.35 (-0.22, 1.66)	0.56 (-0.24, 2.42)	0.80 (-0.31, 3.28)	0.56 (-0.19, 2.22)	-0.13 (-0.25, -0.01)	0.041
	Low SDI	0.04 (-0.04, 0.25)	0.25 (-0.11, 1.13)	0.05 (-0.03, 0.26)	0.22 (-0.09, 1.00)	-0.33 (-0.57, -0.10)	0.005
	Low-middle SDI	0.10 (-0.09, 0.52)	0.39 (-0.20, 1.79)	0.15 (-0.12, 0.76)	0.32 (-0.18, 1.49)	-0.58 (-0.76, -0.40)	<0.001
	Middle SDI	0.18 (-0.15, 0.94)	0.58 (-0.28, 2.61)	0.40 (-0.22, 1.85)	0.48 (-0.20, 2.09)	-0.67 (-0.89, -0.44)	<0.001

GBD region						
Andean Latin America	0.04 (-0.08, 0.31)	0.10 (-0.17, 0.72)	0.06 (-0.13, 0.46)	0.07 (-0.15, 0.55)	-1.31 (-2.06, -0.56)	0.001
Australasia	0.55 (-0.12, 2.06)	0.52 (-0.07, 1.91)	0.77 (-0.08, 2.66)	0.38 (-0.04, 1.31)	-0.99 (-1.06, -0.92)	<0.001
Caribbean	0.18 (-0.11, 0.87)	0.32 (-0.15, 1.49)	0.27 (-0.15, 1.31)	0.23 (-0.13, 1.11)	-1.20 (-1.45, -0.95)	<0.001
Central Asia	0.11 (-0.07, 0.55)	0.21 (-0.11, 1.02)	0.16 (-0.08, 0.74)	0.26 (-0.12, 1.17)	0.61 (0.21, 1.00)	0.002
Central Europe	0.51 (-0.10, 1.87)	0.53 (-0.06, 1.94)	0.65 (-0.27, 2.69)	0.34 (-0.10, 1.32)	-1.49 (-1.58, -1.40)	<0.001
Central Latin America	0.09 (-0.07, 0.47)	0.27 (-0.17, 1.31)	0.13 (-0.07, 0.58)	0.14 (-0.08, 0.63)	-2.06 (-2.37, -1.74)	<0.001
Central Sub-Saharan Africa	0.03 (-0.02, 0.14)	0.16 (-0.04, 0.67)	0.03 (-0.01, 0.13)	0.14 (-0.03, 0.55)	-0.34 (-0.38, -0.30)	<0.001
East Asia	0.25 (-0.29, 1.45)	0.87 (-0.40, 3.84)	0.90 (-0.45, 4.01)	0.79 (-0.28, 3.22)	-0.49 (-0.71, -0.27)	<0.001
Eastern Europe	0.23 (-0.12, 1.02)	0.25 (-0.08, 1.04)	0.43 (-0.20, 1.93)	0.25 (-0.10, 1.10)	0.00 (-0.11, 0.12)	0.933
Eastern Sub-Saharan Africa	0.05 (-0.02, 0.23)	0.32 (-0.04, 1.20)	0.05 (-0.02, 0.20)	0.23 (-0.04, 0.87)	-1.04 (-1.12, -0.97)	<0.001

High-income Asia Pacific	0.72 (-0.16, 2.71)	0.81 (-0.11, 2.87)	1.83 (-0.11, 6.20)	0.49 (-0.06, 1.74)	-1.61 (-1.66, -1.56)	<0.001
High-income North America	1.61 (0.13, 5.02)	1.26 (0.13, 3.85)	1.53 (-0.10, 5.16)	0.77 (-0.05, 2.62)	-1.56 (-1.63, -1.50)	<0.001
North Africa and Middle East	0.19 (-0.09, 0.80)	0.66 (-0.20, 2.77)	0.26 (-0.08, 1.03)	0.54 (-0.13, 2.12)	-0.69 (-0.77, -0.61)	<0.001
Oceania	0.04 (-0.07, 0.28)	0.23 (-0.17, 1.14)	0.05 (-0.06, 0.33)	0.20 (-0.12, 0.96)	-0.49 (-0.58, -0.40)	<0.001
South Asia	0.06 (-0.09, 0.43)	0.31 (-0.22, 1.61)	0.12 (-0.12, 0.70)	0.27 (-0.17, 1.29)	-0.39 (-0.68, -0.10)	0.008
Southeast Asia	0.14 (-0.11, 0.71)	0.47 (-0.25, 2.22)	0.21 (-0.19, 1.13)	0.35 (-0.24, 1.73)	-0.91 (-0.99, -0.84)	<0.001
Southern Latin America	0.16 (-0.14, 0.83)	0.21 (-0.15, 1.00)	0.25 (-0.16, 1.20)	0.19 (-0.11, 0.89)	-0.29 (-0.46, -0.11)	0.002
Southern Sub-Saharan Africa	0.22 (-0.02, 0.78)	0.64 (-0.03, 2.11)	0.11 (-0.04, 0.46)	0.23 (-0.06, 0.98)	-3.24 (-3.32, -3.16)	<0.001
Tropical Latin America	0.47 (-0.04, 1.71)	1.19 (-0.04, 4.17)	0.72 (-0.03, 2.43)	0.67 (-0.03, 2.27)	-1.85 (-1.95, -1.75)	<0.001
Western Europe	1.19 (-0.25, 4.58)	0.84 (-0.09, 3.03)	1.49 (-0.20, 5.37)	0.55 (-0.06, 1.98)	-1.35 (-1.41, -1.28)	<0.001
Western	0.02 (-0.02,	0.10 (-0.04,	0.01 (-0.02,	0.06 (-0.05,	-1.35 (-1.45,	<0.001

	Sub-Saharan Africa	0.11)	0.43)	0.08)	0.32)	-1.26)	
	Global	0.60 (0.14, 1.67)	1.08 (0.25, 3.03)	0.85 (0.20, 2.34)	0.84 (0.19, 2.29)	-0.84 (-0.87, -0.82)	<0.001
	sex						
	Female	0.44 (0.11, 1.20)	0.67 (0.16, 1.83)	0.52 (0.13, 1.37)	0.43 (0.10, 1.14)	-1.42 (-1.44, -1.39)	<0.001
	Male	0.76 (0.17, 2.15)	1.78 (0.40, 5.14)	1.18 (0.28, 3.35)	1.43 (0.34, 4.05)	-0.72 (-0.76, -0.68)	<0.001
Alzheimer's disease and other dementias	SDI quintile						
	High SDI	1.55 (0.36, 4.32)	1.26 (0.29, 3.55)	1.99 (0.47, 5.49)	0.85 (0.20, 2.36)	-1.27 (-1.29, -1.25)	<0.001
	High-middle SDI	0.74 (0.17, 2.07)	1.03 (0.23, 2.89)	1.42 (0.33, 3.85)	0.96 (0.22, 2.60)	-0.25 (-0.34, -0.16)	<0.001
	Low SDI	0.11 (0.03, 0.32)	0.47 (0.11, 1.36)	0.11 (0.03, 0.31)	0.41 (0.10, 1.18)	-0.45 (-0.60, -0.29)	<0.001
	Low-middle SDI	0.24 (0.05, 0.67)	0.75 (0.17, 2.14)	0.34 (0.08, 0.96)	0.63 (0.14, 1.81)	-0.52 (-0.59, -0.45)	<0.001
	Middle SDI	0.42 (0.10, 1.19)	1.11 (0.26, 3.14)	0.78 (0.19, 2.22)	0.88 (0.21, 2.45)	-0.78 (-0.82, -0.75)	<0.001
	GBD region						
	Andean	0.14 (0.03,	0.32 (0.07,	0.21 (0.05,	0.26 (0.06,	-0.70 (-0.78,	<0.001

Latin America	0.41)	0.97)	0.63)	0.75)	-0.63)	
Australasia	0.85 (0.20, 2.39)	0.77 (0.17, 2.16)	1.09 (0.26, 3.03)	0.54 (0.13, 1.50)	-1.17 (-1.22, -1.12)	<0.001
Caribbean	0.37 (0.08, 1.05)	0.61 (0.13, 1.74)	0.54 (0.12, 1.58)	0.46 (0.10, 1.35)	-0.85 (-0.91, -0.80)	<0.001
Central Asia	0.23 (0.05, 0.67)	0.42 (0.10, 1.20)	0.31 (0.07, 0.92)	0.49 (0.11, 1.41)	0.55 (0.47, 0.63)	<0.001
Central Europe	0.76 (0.18, 2.14)	0.76 (0.18, 2.21)	1.17 (0.27, 3.20)	0.56 (0.13, 1.55)	-0.94 (-0.97, -0.91)	<0.001
Central Latin America	0.20 (0.05, 0.60)	0.55 (0.12, 1.61)	0.26 (0.06, 0.72)	0.28 (0.06, 0.78)	-2.15 (-2.22, -2.09)	<0.001
Central Sub-Saharan Africa	0.06 (0.01, 0.17)	0.27 (0.06, 0.79)	0.05 (0.01, 0.16)	0.22 (0.05, 0.64)	-0.57 (-0.63, -0.51)	<0.001
East Asia	0.68 (0.15, 1.89)	1.67 (0.37, 4.69)	1.72 (0.41, 4.88)	1.38 (0.32, 3.87)	-0.64 (-0.77, -0.52)	<0.001
Eastern Europe	0.44 (0.10, 1.25)	0.42 (0.09, 1.21)	0.80 (0.18, 2.26)	0.45 (0.10, 1.28)	0.23 (0.13, 0.34)	<0.001
Eastern Sub-Saharan Africa	0.10 (0.02, 0.28)	0.48 (0.11, 1.37)	0.08 (0.02, 0.23)	0.35 (0.08, 0.99)	-1.08 (-1.12, -1.04)	<0.001
High-income Asia Pacific	1.14 (0.27, 3.20)	1.19 (0.27, 3.29)	2.55 (0.61, 6.94)	0.72 (0.17, 1.97)	-1.61 (-1.66, -1.57)	<0.001
High-income	1.94 (0.46,	1.48 (0.35,	2.10 (0.51,	1.05 (0.25,	-1.10 (-1.16,	<0.001

North America	5.37)	4.09)	5.75)	2.89)	-1.04)	
North Africa and Middle East	0.35 (0.08, 0.97)	1.12 (0.25, 3.26)	0.43 (0.10, 1.24)	0.85 (0.19, 2.46)	-0.85 (-0.89, -0.81)	<0.001
Oceania	0.13 (0.03, 0.39)	0.51 (0.11, 1.42)	0.14 (0.03, 0.43)	0.41 (0.09, 1.17)	-0.74 (-0.77, -0.70)	<0.001
South Asia	0.19 (0.04, 0.56)	0.67 (0.15, 1.93)	0.31 (0.07, 0.87)	0.55 (0.13, 1.59)	-0.56 (-0.79, -0.34)	<0.001
Southeast Asia	0.32 (0.07, 0.91)	0.93 (0.21, 2.68)	0.51 (0.12, 1.47)	0.75 (0.17, 2.19)	-0.70 (-0.74, -0.66)	<0.001
Southern Latin America	0.37 (0.09, 1.08)	0.44 (0.10, 1.28)	0.52 (0.12, 1.50)	0.38 (0.09, 1.11)	-0.46 (-0.52, -0.40)	<0.001
Southern Sub-Saharan Africa	0.32 (0.08, 0.89)	0.88 (0.21, 2.37)	0.19 (0.04, 0.56)	0.37 (0.08, 1.14)	-2.71 (-2.80, -2.62)	<0.001
Tropical Latin America	0.67 (0.15, 1.93)	1.61 (0.36, 4.56)	0.98 (0.23, 2.65)	0.91 (0.22, 2.49)	-1.81 (-1.85, -1.77)	<0.001
Western Europe	1.86 (0.43, 5.32)	1.21 (0.28, 3.41)	2.16 (0.50, 5.97)	0.78 (0.18, 2.18)	-1.40 (-1.43, -1.37)	<0.001
Western Sub-Saharan Africa	0.05 (0.01, 0.14)	0.18 (0.04, 0.52)	0.04 (0.01, 0.10)	0.14 (0.03, 0.41)	-0.83 (-0.89, -0.77)	<0.001

	Global	0.03 (0.03, 0.04)	0.04 (0.03, 0.04)	0.02 (0.02, 0.03)	0.02 (0.02, 0.02)	-2.01 (-2.24, -1.78)	<0.001
	sex						
	Female	0.03 (0.02, 0.03)	0.03 (0.03, 0.04)	0.02 (0.02, 0.03)	0.02 (0.02, 0.02)	-1.86 (-2.03, -1.69)	<0.001
	Male	0.04 (0.03, 0.04)	0.05 (0.04, 0.05)	0.02 (0.02, 0.03)	0.02 (0.02, 0.03)	-2.16 (-2.38, -1.93)	<0.001
	SDI quintile						
	High SDI	0.13 (0.12, 0.15)	0.12 (0.10, 0.13)	0.12 (0.10, 0.14)	0.07 (0.06, 0.08)	-1.47 (-1.63, -1.30)	<0.001
Multiple sclerosis	High-middle SDI	0.04 (0.03, 0.05)	0.04 (0.03, 0.05)	0.03 (0.02, 0.03)	0.02 (0.02, 0.02)	-2.19 (-2.68, -1.69)	<0.001
	Low SDI	0.00 (0.00, 0.00)	0.00 (0.00, 0.00)	0.00 (0.00, 0.00)	0.00 (0.00, 0.00)	1.73 (1.58, 1.89)	<0.001
	Low-middle SDI	0.00 (0.00, 0.00)	0.00 (0.00, 0.00)	0.00 (0.00, 0.00)	0.00 (0.00, 0.00)	0.82 (0.68, 0.96)	<0.001
	Middle SDI	0.00 (0.00, 0.00)	0.00 (0.00, 0.01)	0.01 (0.00, 0.01)	0.00 (0.00, 0.01)	0.05 (-0.03, 0.12)	0.214
	GBD region						
	Andean Latin America	0.00 (0.00, 0.00)	0.00 (0.00, 0.00)	0.00 (0.00, 0.01)	0.00 (0.00, 0.01)	0.93 (0.04, 1.82)	0.041
	Australasia	0.06 (0.05,	0.06 (0.05,	0.06 (0.05,	0.04 (0.03,	-1.23 (-1.70,	<0.001

	0.08)	0.07)	0.07)	0.05)	-0.76)	
Caribbean	0.02 (0.01, 0.02)	0.02 (0.02, 0.02)	0.02 (0.02, 0.03)	0.02 (0.01, 0.02)	-0.32 (-0.52, -0.12)	0.002
Central Asia	0.01 (0.01, 0.02)	0.02 (0.01, 0.02)	0.01 (0.01, 0.01)	0.01 (0.01, 0.02)	-1.56 (-2.63, -0.48)	0.005
Central Europe	0.23 (0.20, 0.26)	0.19 (0.17, 0.22)	0.12 (0.10, 0.15)	0.08 (0.07, 0.10)	-2.80 (-2.93, -2.67)	<0.001
Central Latin America	0.00 (0.00, 0.01)	0.01 (0.01, 0.01)	0.01 (0.01, 0.01)	0.01 (0.01, 0.01)	0.34 (0.02, 0.67)	0.037
Central Sub-Saharan Africa	0.00 (0.00, 0.00)	0.00 (0.00, 0.00)	0.00 (0.00, 0.00)	0.00 (0.00, 0.00)	1.41 (1.23, 1.59)	<0.001
East Asia	0.00 (0.00, 0.00)	0.00 (0.00, 0.00)	0.00 (0.00, 0.00)	0.00 (0.00, 0.00)	0.31 (-0.30, 0.92)	0.323
Eastern Europe	0.08 (0.07, 0.10)	0.07 (0.06, 0.08)	0.07 (0.06, 0.08)	0.05 (0.04, 0.06)	-1.17 (-1.98, -0.36)	0.005
Eastern Sub-Saharan Africa	0.00 (0.00, 0.00)	0.00 (0.00, 0.00)	0.00 (0.00, 0.00)	0.00 (0.00, 0.00)	0.82 (0.75, 0.90)	<0.001
High-income Asia Pacific	0.01 (0.01, 0.01)	0.01 (0.00, 0.01)	0.01 (0.00, 0.01)	0.00 (0.00, 0.00)	-1.71 (-2.27, -1.16)	<0.001
High-income North America	0.15 (0.13, 0.18)	0.14 (0.12, 0.16)	0.17 (0.14, 0.20)	0.11 (0.09, 0.13)	-0.86 (-1.10, -0.61)	<0.001
North Africa	0.01 (0.00,	0.01 (0.01,	0.02 (0.01,	0.02 (0.01,	0.75 (0.65,	<0.001

	and Middle East	0.01)	0.02)	0.02)	0.02)	0.84)	
	Oceania	0.00 (0.00, 0.00)	0.00 (0.00, 0.00)	0.00 (0.00, 0.00)	0.00 (0.00, 0.00)	-0.01 (-0.15, 0.13)	0.856
	South Asia	0.00 (0.00, 0.00)	0.00 (0.00, 0.00)	0.00 (0.00, 0.00)	0.00 (0.00, 0.00)	0.70 (0.54, 0.87)	<0.001
	Southeast Asia	0.00 (0.00, 0.00)	0.00 (0.00, 0.00)	0.00 (0.00, 0.00)	0.00 (0.00, 0.00)	0.55 (0.42, 0.67)	<0.001
	Southern Latin America	0.04 (0.03, 0.05)	0.04 (0.03, 0.05)	0.02 (0.02, 0.03)	0.02 (0.02, 0.02)	-2.39 (-2.81, -1.97)	<0.001
	Southern Sub-Saharan Africa	0.01 (0.00, 0.01)	0.01 (0.01, 0.01)	0.00 (0.00, 0.01)	0.01 (0.00, 0.01)	-1.69 (-2.10, -1.27)	<0.001
	Tropical Latin America	0.01 (0.01, 0.01)	0.02 (0.01, 0.02)	0.01 (0.01, 0.02)	0.01 (0.01, 0.01)	-1.07 (-1.43, -0.70)	<0.001
	Western Europe	0.17 (0.15, 0.19)	0.13 (0.11, 0.15)	0.15 (0.13, 0.18)	0.09 (0.08, 0.11)	-1.13 (-1.40, -0.86)	<0.001
	Western Sub-Saharan Africa	0.00 (0.00, 0.00)	0.00 (0.00, 0.00)	0.00 (0.00, 0.00)	0.00 (0.00, 0.00)	0.64 (0.57, 0.70)	<0.001
Parkinson's disease	Global	-0.30 (-0.39, -0.22)	-0.46 (-0.59, -0.33)	-0.38 (-0.50, -0.28)	-0.36 (-0.48, -0.26)	-0.73 (-0.84, -0.61)	<0.001

sex						
Female	-0.09 (-0.11, -0.07)	-0.12 (-0.15, -0.09)	-0.09 (-0.13, -0.07)	-0.08 (-0.11, -0.06)	-1.30 (-1.40, -1.20)	<0.001
Male	-0.50 (-0.65, -0.37)	-0.96 (-1.25, -0.70)	-0.67 (-0.88, -0.48)	-0.74 (-0.98, -0.54)	-0.83 (-0.97, -0.70)	<0.001
SDI quintile						
High SDI	-0.57 (-0.76, -0.40)	-0.43 (-0.58, -0.30)	-0.71 (-1.01, -0.46)	-0.33 (-0.46, -0.22)	-0.92 (-1.04, -0.79)	<0.001
High-middle SDI	-0.43 (-0.55, -0.31)	-0.51 (-0.65, -0.37)	-0.64 (-0.84, -0.47)	-0.42 (-0.56, -0.31)	-0.57 (-0.74, -0.40)	<0.001
Low SDI	-0.07 (-0.09, -0.05)	-0.22 (-0.29, -0.15)	-0.06 (-0.08, -0.04)	-0.18 (-0.25, -0.12)	-0.53 (-0.95, -0.12)	0.012
Low-middle SDI	-0.14 (-0.19, -0.10)	-0.36 (-0.49, -0.25)	-0.19 (-0.25, -0.14)	-0.31 (-0.42, -0.22)	-0.42 (-0.50, -0.34)	<0.001
Middle SDI	-0.25 (-0.32, -0.18)	-0.54 (-0.68, -0.39)	-0.39 (-0.52, -0.28)	-0.40 (-0.53, -0.29)	-0.93 (-1.11, -0.74)	<0.001
GBD region						
Andean Latin America	-0.10 (-0.15, -0.07)	-0.22 (-0.33, -0.14)	-0.16 (-0.24, -0.10)	-0.19 (-0.29, -0.11)	-0.39 (-0.80, 0.02)	0.063
Australasia	-0.37 (-0.52, -0.25)	-0.31 (-0.43, -0.20)	-0.38 (-0.58, -0.22)	-0.20 (-0.30, -0.12)	-1.45 (-1.57, -1.32)	<0.001
Caribbean	-0.21 (-0.29, -0.14)	-0.30 (-0.42, -0.20)	-0.29 (-0.42, -0.19)	-0.26 (-0.37, -0.17)	-0.50 (-0.77, -0.23)	<0.001

Central Asia	-0.13 (-0.17, -0.10)	-0.22 (-0.29, -0.16)	-0.16 (-0.21, -0.12)	-0.25 (-0.31, -0.18)	0.29 (0.12, 0.46)	0.001
Central Europe	-0.48 (-0.63, -0.34)	-0.42 (-0.55, -0.30)	-0.65 (-0.88, -0.45)	-0.31 (-0.42, -0.21)	-0.93 (-1.00, -0.86)	<0.001
Central Latin America	-0.12 (-0.15, -0.09)	-0.29 (-0.37, -0.21)	-0.14 (-0.19, -0.09)	-0.15 (-0.20, -0.10)	-2.12 (-2.31, -1.92)	<0.001
Central Sub-Saharan Africa	-0.03 (-0.04, -0.02)	-0.11 (-0.16, -0.07)	-0.02 (-0.03, -0.01)	-0.08 (-0.12, -0.05)	-0.90 (-1.02, -0.79)	<0.001
East Asia	-0.43 (-0.56, -0.31)	-0.79 (-1.02, -0.57)	-0.82 (-1.11, -0.58)	-0.59 (-0.80, -0.42)	-0.94 (-1.13, -0.75)	<0.001
Eastern Europe	-0.29 (-0.38, -0.21)	-0.24 (-0.32, -0.17)	-0.43 (-0.59, -0.31)	-0.25 (-0.34, -0.18)	0.14 (-0.33, 0.60)	0.570
Eastern Sub-Saharan Africa	-0.04 (-0.06, -0.03)	-0.16 (-0.22, -0.11)	-0.03 (-0.05, -0.02)	-0.11 (-0.17, -0.07)	-1.12 (-1.20, -1.03)	<0.001
High-income Asia Pacific	-0.43 (-0.54, -0.32)	-0.39 (-0.50, -0.29)	-0.73 (-1.01, -0.49)	-0.24 (-0.32, -0.16)	-1.64 (-1.77, -1.51)	<0.001
High-income North America	-0.48 (-0.66, -0.32)	-0.36 (-0.49, -0.24)	-0.74 (-1.11, -0.43)	-0.38 (-0.58, -0.23)	0.21 (0.06, 0.37)	0.007
North Africa and Middle East	-0.17 (-0.23, -0.12)	-0.47 (-0.63, -0.33)	-0.19 (-0.25, -0.13)	-0.34 (-0.45, -0.23)	-1.05 (-1.13, -0.98)	<0.001
Oceania	-0.10 (-0.13,	-0.28 (-0.38,	-0.09 (-0.12,	-0.21 (-0.29,	-0.95 (-1.06,	<0.001

	-0.06)	-0.19)	-0.06)	-0.13)	-0.84)	
South Asia	-0.13 (-0.19, -0.09)	-0.36 (-0.51, -0.23)	-0.18 (-0.25, -0.12)	-0.29 (-0.40, -0.19)	-0.74 (-1.37, -0.10)	0.023
Southeast Asia	-0.19 (-0.25, -0.14)	-0.46 (-0.61, -0.34)	-0.30 (-0.39, -0.22)	-0.40 (-0.52, -0.29)	-0.50 (-0.59, -0.40)	<0.001
Southern Latin America	-0.25 (-0.37, -0.16)	-0.28 (-0.41, -0.17)	-0.29 (-0.43, -0.16)	-0.21 (-0.32, -0.12)	-0.84 (-1.06, -0.62)	<0.001
Southern Sub-Saharan Africa	-0.10 (-0.13, -0.07)	-0.25 (-0.32, -0.18)	-0.09 (-0.11, -0.06)	-0.15 (-0.19, -0.11)	-1.60 (-1.91, -1.30)	<0.001
Tropical Latin America	-0.21 (-0.29, -0.15)	-0.43 (-0.60, -0.31)	-0.27 (-0.41, -0.16)	-0.26 (-0.38, -0.15)	-1.65 (-1.83, -1.47)	<0.001
Western Europe	-0.83 (-1.13, -0.57)	-0.50 (-0.68, -0.34)	-0.82 (-1.19, -0.52)	-0.32 (-0.46, -0.21)	-1.42 (-1.58, -1.25)	<0.001
Western Sub-Saharan Africa	-0.03 (-0.04, -0.02)	-0.09 (-0.12, -0.06)	-0.02 (-0.03, -0.01)	-0.08 (-0.11, -0.05)	-0.31 (-0.39, -0.23)	<0.001

Supplementary Table S3. Joinpoint regression of DALYs for global and six regional levels from 1990 to 2021.

Location	Disease	Both				Female				Male			
		**Segment**	**Period**	**APC (95% CI)**	**P value**	**Segment**	**Period**	**APC (95% CI)**	**P value**	**Segment**	**Period**	**APC (95% CI)**	**P value**
Global	Neurological disorders	1	1990 - 1995	-0.73 (-0.85, -0.60)	<0.001	1	1990 - 1995	-0.51 (-0.57, -0.45)	<0.001	1	1990 - 1994	-0.92 (-1.23, -0.61)	<0.001
	Neurological disorders	2	1995 - 2004	-1.76 (-1.82, -1.70)	<0.001	2	1995 - 2000	-1.50 (-1.58, -1.42)	<0.001	2	1994 - 2003	-1.90 (-2.00, -1.79)	<0.001
	Neurological disorders	3	2004 - 2011	-1.40 (-1.49, -1.30)	<0.001	3	2000 - 2013	-1.85 (-1.86, -1.83)	<0.001	3	2003 - 2010	-1.16 (-1.34, -0.98)	<0.001
	Neurological disorders	4	2011 - 2019	-0.95 (-1.04, -0.87)	<0.001	4	2013 - 2019	-1.72 (-1.78, -1.66)	<0.001	4	2010 - 2019	-0.42 (-0.54, -0.30)	<0.001
	Neurological disorders	5	2019 - 2021	0.36 (-0.26, 0.97)	0.237	5	2019 - 2021	-0.86 (-1.13, -0.59)	<0.001	5	2019 - 2021	1.23 (0.14, 2.33)	0.029
	Neurological disorders	6	...	...	...	6	...	...	...	6	...	...	...
	Neurological disorders	AAPC	1990 - 2021	-1.17 (-1.22, -1.12)	<0.001	AAPC	1990 - 2021	-1.49 (-1.51, -1.46)	<0.001	AAPC	1990 - 2021	-0.98 (-1.07, -0.88)	<0.001
High SDI	Neurological disorders	1	1990 - 1995	-1.04 (-1.08, -1.00)	<0.001	1	1990 - 1995	-0.91 (-0.97, -0.85)	<0.001	1	1990 - 1995	-1.29 (-1.37, -1.20)	<0.001
	Neurological disorders	2	1995 - 2001	-2.04 (-2.08, -1.99)	<0.001	2	1995 - 2001	-1.54 (-1.60, -1.48)	<0.001	2	1995 - 2004	-2.76 (-2.80, -2.71)	<0.001
	Neurological disorders	3	2001 - 2004	-2.22 (-2.41, -2.03)	<0.001	3	2001 - 2004	-1.88 (-2.14, -1.62)	<0.001	3	2004 - 2010	-1.97 (-2.07, -1.88)	<0.001
	Neurological disorders	4	2004 - 2010	-1.64 (-1.68, -1.60)	<0.001	4	2004 - 2009	-1.49 (-1.57, -1.41)	<0.001	4	2010 - 2016	-1.14 (-1.24, -1.05)	<0.001

	Neurological disorders	5	2010 - 2015	-1.23 (-1.29, -1.17)	<0.001	5	2009 - 2014	-1.35 (-1.43, -1.27)	<0.001	5	2016 - 2021	-0.43 (-0.52, -0.33)	<0.001
	Neurological disorders	6	2015 - 2021	-0.83 (-0.86, -0.79)	<0.001	6	2014 - 2021	-1.12 (-1.16, -1.09)	<0.001	6	...	...	...
	Neurological disorders	AAPC	1990 - 2021	-1.45 (-1.48, -1.43)	<0.001	AAPC	1990 - 2021	-1.34 (-1.37, -1.31)	<0.001	AAPC	1990 - 2021	-1.68 (-1.72, -1.65)	<0.001
	Neurological disorders	1	1990 - 1995	0.04 (-0.51, 0.59)	0.885	1	1990 - 1995	0.59 (0.40, 0.78)	<0.001	1	1990 - 2010	-0.97 (-1.05, -0.88)	<0.001
	Neurological disorders	2	1995 - 2011	-0.78 (-0.88, -0.67)	<0.001	2	1995 - 2003	-0.29 (-0.40, -0.17)	<0.001	2	2010 - 2021	0.21 (-0.02, 0.43)	0.070
	Neurological disorders	3	2011 - 2021	-0.21 (-0.42, -0.01)	0.039	3	2003 - 2016	-0.93 (-0.98, -0.88)	<0.001	3	...	...	...
High-middle SDI	Neurological disorders	4	...	...	...	4	2016 - 2019	-1.95 (-2.77, -1.11)	<0.001	4	...	...	...
	Neurological disorders	5	...	...	...	5	2019 - 2021	0.39 (-0.43, 1.23)	0.331	5	...	...	...
	Neurological disorders	6	...	...	...	6	...	...	...	6	...	...	...
	Neurological disorders	AAPC	1990 - 2021	-0.46 (-0.58, -0.35)	<0.001	AAPC	1990 - 2021	-0.53 (-0.63, -0.43)	<0.001	AAPC	1990 - 2021	-0.55 (-0.64, -0.46)	<0.001
	Neurological disorders	1	1990 - 1994	0.08 (-0.65, 0.81)	0.828	1	1990 - 1993	1.26 (0.98, 1.54)	<0.001	1	1990 - 2004	-1.42 (-1.58, -1.26)	<0.001
Middle SDI	Neurological disorders	2	1994 - 2010	-1.59 (-1.68, -1.49)	<0.001	2	1993 - 1996	0.07 (-0.47, 0.61)	0.795	2	2004 - 2019	-0.22 (-0.38, -0.05)	0.011
	Neurological disorders	3	2010 - 2019	-0.73 (-0.99, -0.47)	<0.001	3	1996 - 2001	-1.64 (-1.81, -1.47)	<0.001	3	2019 - 2021	2.39 (-1.06, 5.95)	0.168

	Neurological disorders	4	2019 - 2021	1.58 (-0.79, 4.00)	0.183	4	2001 - 2013	-2.91 (-2.95, -2.87)	<0.001	4	...	...	...
	Neurological disorders	5	...	...	...	5	2013 - 2019	-1.79 (-1.91, -1.67)	<0.001	5	...	...	...
	Neurological disorders	6	...	...	...	6	2019 - 2021	0.53 (0.01, 1.05)	0.046	6	...	...	...
Low-middle SDI	Neurological disorders	AAPC	1990 - 2021	-0.92 (-1.11, -0.73)	<0.001	AAPC	1990 - 2021	-1.59 (-1.66, -1.51)	<0.001	AAPC	1990 - 2021	-0.60 (-0.83, -0.37)	<0.001
	Neurological disorders	1	1990 - 2002	-0.59 (-0.71, -0.47)	<0.001	1	1990 - 2000	0.01 (-0.04, 0.06)	0.641	1	1990 - 2015	-0.75 (-0.80, -0.69)	<0.001
	Neurological disorders	2	2002 - 2018	-1.06 (-1.15, -0.98)	<0.001	2	2000 - 2005	-0.57 (-0.76, -0.38)	<0.001	2	2015 - 2021	-0.07 (-0.59, 0.45)	0.784
	Neurological disorders	3	2018 - 2021	-0.06 (-1.16, 1.04)	0.909	3	2005 - 2011	-1.37 (-1.51, -1.23)	<0.001	3	...	...	...
	Neurological disorders	4	...	...	...	4	2011 - 2015	-1.80 (-2.11, -1.48)	<0.001	4	...	...	...
	Neurological disorders	5	...	...	...	5	2015 - 2021	-1.07 (-1.18, -0.96)	<0.001	5	...	...	...
	Neurological disorders	6	...	...	...	6	...	...	...	6	...	...	...
Low SDI	Neurological disorders	AAPC	1990 - 2021	-0.78 (-0.90, -0.67)	<0.001	AAPC	1990 - 2021	-0.80 (-0.86, -0.74)	<0.001	AAPC	1990 - 2021	-0.61 (-0.72, -0.51)	<0.001
	Neurological disorders	1	1990 - 2005	-0.05 (-0.11, 0.00)	0.056	1	1990 - 1999	0.74 (0.65, 0.82)	<0.001	1	1990 - 1995	-1.11 (-1.47, -0.74)	<0.001
	Neurological disorders	2	2005 - 2012	-1.07 (-1.28, -0.87)	<0.001	2	1999 - 2004	1.13 (0.83, 1.44)	<0.001	2	1995 - 2005	-0.58 (-0.72, -0.43)	<0.001

	Neurological disorders	3	2012 - 2015	-3.19 (-4.44, -1.93)	<0.001	3	2004 - 2011	-0.55 (-0.71, -0.40)	<0.001	3	2005 - 2012	-1.34 (-1.61, -1.06)	<0.001
	Neurological disorders	4	2015 - 2021	0.05 (-0.17, 0.27)	0.651	4	2011 - 2015	-3.32 (-3.78, -2.85)	<0.001	4	2012 - 2015	-2.47 (-4.12, -0.80)	0.006
	Neurological disorders	5	...	...	...	5	2015 - 2021	-0.34 (-0.50, -0.18)	<0.001	5	2015 - 2021	0.45 (0.16, 0.75)	0.005
	Neurological disorders	6	...	...	...	6	...	...	...	6	...	...	...
	Neurological disorders	AAPC	1990 - 2021	-0.57 (-0.71, -0.44)	<0.001	AAPC	1990 - 2021	-0.23 (-0.32, -0.14)	<0.001	AAPC	1990 - 2021	-0.82 (-1.01, -0.64)	<0.001
	Alzheimer's disease and other dementias	1	1990 - 1995	-0.54 (-0.63, -0.45)	<0.001	1	1990 - 1996	-0.43 (-0.49, -0.37)	<0.001	1	1990 - 1995	-0.77 (-0.86, -0.68)	<0.001
Global	Alzheimer's disease and other dementias	2	1995 - 2005	-1.23 (-1.26, -1.19)	<0.001	2	1996 - 2013	-1.67 (-1.68, -1.65)	<0.001	2	1995 - 2000	-1.35 (-1.48, -1.23)	<0.001
	Alzheimer's disease and other dementias	3	2005 - 2019	-0.68 (-0.70, -0.66)	<0.001	3	2013 - 2019	-1.54 (-1.62, -1.46)	<0.001	3	2000 - 2006	-0.95 (-1.03, -0.86)	<0.001
	Alzheimer's disease and other	4	2019 - 2021	0.06 (-0.33, 0.46)	0.740	4	2019 - 2021	-0.78 (-1.12, -0.44)	<0.001	4	2006 - 2019	-0.40 (-0.42, -0.38)	<0.001

	dementias												
	Alzheimer's disease and other dementias	5	...	...	...	5	...	...	...	5	2019 - 2021	0.31 (-0.09, 0.70)	0.120
	Alzheimer's disease and other dementias	6	...	...	...	6	...	...	...	6	...	...	...
	Alzheimer's disease and other dementias	AAPC	1990 - 2021	-0.79 (-0.82, -0.76)	<0.001	AAPC	1990 - 2021	-1.35 (-1.38, -1.32)	<0.001	AAPC	1990 - 2021	-0.67 (-0.71, -0.64)	<0.001
	Alzheimer's disease and other dementias	1	1990 - 1995	-1.00 (-1.05, -0.95)	<0.001	1	1990 - 1995	-0.91 (-0.97, -0.86)	<0.001	1	1990 - 1995	-1.25 (-1.31, -1.19)	<0.001
High SDI	Alzheimer's disease and other dementias	2	1995 - 2004	-1.74 (-1.77, -1.72)	<0.001	2	1995 - 2004	-1.69 (-1.71, -1.66)	<0.001	2	1995 - 2004	-2.02 (-2.05, -1.99)	<0.001
	Alzheimer's disease and other dementias	3	2004 - 2009	-1.41 (-1.48, -1.34)	<0.001	3	2004 - 2009	-1.30 (-1.38, -1.23)	<0.001	3	2004 - 2009	-1.78 (-1.86, -1.70)	<0.001
	Alzheimer's	4	2009 -	-1.13 (-1.20,	<0.001	4	2009 -	-1.10 (-1.18,	<0.001	4	2009 -	-1.46 (-1.72,	<0.001

	disease and other dementias		2014	-1.06)			2014	-1.03)			2012	-1.20)	
	Alzheimer's disease and other dementias	5	2014 - 2021	-0.69 (-0.72, -0.66)	<0.001	5	2014 - 2021	-0.97 (-1.00, -0.94)	<0.001	5	2012 - 2015	-1.13 (-1.39, -0.86)	<0.001
	Alzheimer's disease and other dementias	6	...	...	...	6	...	...	...	6	2015 - 2021	-0.63 (-0.67, -0.58)	<0.001
	Alzheimer's disease and other dementias	AAPC	1990 - 2021	-1.23 (-1.25, -1.21)	<0.001	AAPC	1990 - 2021	-1.24 (-1.27, -1.22)	<0.001	AAPC	1990 - 2021	-1.45 (-1.49, -1.41)	<0.001
High-middle SDI	Alzheimer's disease and other dementias	1	1990 - 1995	0.06 (-0.04, 0.16)	0.245	1	1990 - 1996	0.66 (0.52, 0.80)	<0.001	1	1990 - 2000	-0.59 (-0.64, -0.54)	<0.001
	Alzheimer's disease and other dementias	2	1995 - 2006	-0.27 (-0.31, -0.24)	<0.001	2	1996 - 2003	-0.35 (-0.49, -0.21)	<0.001	2	2000 - 2007	-0.31 (-0.41, -0.20)	<0.001
	Alzheimer's disease and other	3	2006 - 2016	-0.02 (-0.06, 0.02)	0.379	3	2003 - 2016	-0.87 (-0.92, -0.82)	<0.001	3	2007 - 2016	0.02 (-0.05, 0.09)	0.608

	dementias												
	Alzheimer's disease and other dementias	4	2016 - 2019	-0.93 (-1.39, -0.47)	0.001	4	2016 - 2019	-1.83 (-2.65, -1.01)	<0.001	4	2016 - 2019	-0.86 (-1.45, -0.26)	0.007
	Alzheimer's disease and other dementias	5	2019 - 2021	0.86 (0.41, 1.32)	0.001	5	2019 - 2021	0.44 (-0.37, 1.25)	0.270	5	2019 - 2021	0.86 (0.26, 1.47)	0.008
	Alzheimer's disease and other dementias	6	...	...	...	6	...	...	...	6	...	...	...
	Alzheimer's disease and other dementias	AAPC	1990 - 2021	-0.13 (-0.18, -0.07)	<0.001	AAPC	1990 - 2021	-0.47 (-0.57, -0.37)	<0.001	AAPC	1990 - 2021	-0.28 (-0.36, -0.21)	<0.001
	Alzheimer's disease and other dementias	1	1990 - 1995	-0.18 (-0.30, -0.05)	0.008	1	1990 - 1993	1.15 (0.92, 1.39)	<0.001	1	1990 - 1995	-0.54 (-0.63, -0.45)	<0.001
Middle SDI	Alzheimer's disease and other dementias	2	1995 - 2005	-1.45 (-1.50, -1.40)	<0.001	2	1993 - 1996	0.03 (-0.43, 0.51)	0.878	2	1995 - 2000	-1.46 (-1.59, -1.34)	<0.001
	Alzheimer's	3	2005 -	-0.52 (-0.56,	<0.001	3	1996 -	-1.76 (-1.90,	<0.001	3	2000 -	-0.84 (-0.96,	<0.001

	disease and other dementias		2016	-0.47)			2001	-1.61)			2005	-0.72)	
	Alzheimer's disease and other dementias	4	2016 - 2019	-1.13 (-1.68, -0.58)	<0.001	4	2001 - 2013	-2.91 (-2.94, -2.88)	<0.001	4	2005 - 2016	0.04 (0.01, 0.07)	0.010
	Alzheimer's disease and other dementias	5	2019 - 2021	0.79 (0.25, 1.34)	0.007	5	2013 - 2019	-1.77 (-1.87, -1.66)	<0.001	5	2016 - 2019	-1.07 (-1.46, -0.67)	<0.001
	Alzheimer's disease and other dementias	6	...	...	...	6	2019 - 2021	0.34 (-0.12, 0.81)	0.135	6	2019 - 2021	1.21 (0.81, 1.60)	<0.001
	Alzheimer's disease and other dementias	AAPC	1990 - 2021	-0.74 (-0.81, -0.67)	<0.001	AAPC	1990 - 2021	-1.63 (-1.69, -1.56)	<0.001	AAPC	1990 - 2021	-0.47 (-0.52, -0.42)	<0.001
Low-middle SDI	Alzheimer's disease and other dementias	1	1990 - 1996	-0.48 (-0.58, -0.37)	<0.001	1	1990 - 1998	0.14 (0.08, 0.21)	<0.001	1	1990 - 1997	-0.61 (-0.68, -0.54)	<0.001
	Alzheimer's disease and other	2	1996 - 2009	-0.75 (-0.78, -0.71)	<0.001	2	1998 - 2006	-0.44 (-0.53, -0.36)	<0.001	2	1997 - 2000	-0.85 (-1.39, -0.30)	0.005

	dementias												
	Alzheimer's disease and other dementias	3	2009 - 2016	-0.83 (-0.94, -0.72)	<0.001	3	2006 - 2015	-1.41 (-1.48, -1.35)	<0.001	3	2000 - 2006	-0.51 (-0.63, -0.39)	<0.001
	Alzheimer's disease and other dementias	4	2016 - 2021	-0.29 (-0.43, -0.15)	<0.001	4	2015 - 2021	-1.20 (-1.31, -1.09)	<0.001	4	2006 - 2017	-0.39 (-0.43, -0.35)	<0.001
	Alzheimer's disease and other dementias	5	...	...	...	5	...	...	...	5	2017 - 2021	0.22 (0.04, 0.39)	0.021
	Alzheimer's disease and other dementias	6	...	...	...	6	...	...	...	6	...	...	...
	Alzheimer's disease and other dementias	AAPC	1990 - 2021	-0.64 (-0.68, -0.60)	<0.001	AAPC	1990 - 2021	-0.72 (-0.76, -0.69)	<0.001	AAPC	1990 - 2021	-0.43 (-0.49, -0.37)	<0.001
Low SDI	Alzheimer's disease and other dementias	1	1990 - 1995	0.02 (-0.12, 0.16)	0.773	1	1990 - 1995	0.80 (0.61, 0.99)	<0.001	1	1990 - 1994	-0.39 (-0.59, -0.19)	0.001
	Alzheimer's	2	1995 -	-0.68 (-0.88,	<0.001	2	1995 -	0.26 (-0.01,	0.054	2	1994 -	-0.96 (-1.07,	<0.001

	disease and other dementias		2000	-0.48)			2000	0.54)			2001	-0.85)	
	Alzheimer's disease and other dementias	3	2000 - 2003	-0.02 (-0.67, 0.63)	0.949	3	2000 - 2003	1.82 (0.95, 2.70)	<0.001	3	2001 - 2006	-0.73 (-0.94, -0.53)	<0.001
	Alzheimer's disease and other dementias	4	2003 - 2013	-0.79 (-0.85, -0.73)	<0.001	4	2003 - 2013	-0.48 (-0.56, -0.40)	<0.001	4	2006 - 2009	-1.26 (-1.90, -0.62)	0.001
	Alzheimer's disease and other dementias	5	2013 - 2017	-1.21 (-1.54, -0.89)	<0.001	5	2013 - 2016	-2.78 (-3.62, -1.94)	<0.001	5	2009 - 2018	-0.58 (-0.65, -0.51)	<0.001
	Alzheimer's disease and other dementias	6	2017 - 2021	-0.47 (-0.68, -0.27)	<0.001	6	2016 - 2021	-0.76 (-0.95, -0.57)	<0.001	6	2018 - 2021	-0.12 (-0.45, 0.21)	0.440
	Alzheimer's disease and other dementias	AAPC	1990 - 2021	-0.58 (-0.67, -0.50)	<0.001	AAPC	1990 - 2021	-0.21 (-0.33, -0.08)	0.001	AAPC	1990 - 2021	-0.69 (-0.77, -0.61)	<0.001
Global	Multiple sclerosis	1	1990 - 1995	-1.07 (-1.32, -0.82)	<0.001	1	1990 - 1998	-1.44 (-1.55, -1.33)	<0.001	1	1990 - 1995	-0.85 (-1.12, -0.59)	<0.001

	Multiple sclerosis	2	1995 - 1998	-2.09 (-3.18, -0.98)	0.001	2	1998 - 2002	-1.05 (-1.55, -0.56)	<0.001	2	1995 - 1998	-2.48 (-3.66, -1.29)	0.001
	Multiple sclerosis	3	1998 - 2002	-1.15 (-1.70, -0.60)	<0.001	3	2002 - 2008	-2.33 (-2.55, -2.10)	<0.001	3	1998 - 2002	-1.40 (-2.00, -0.79)	<0.001
	Multiple sclerosis	4	2002 - 2013	-2.57 (-2.66, -2.48)	<0.001	4	2008 - 2013	-2.69 (-3.01, -2.37)	<0.001	4	2002 - 2013	-2.65 (-2.75, -2.55)	<0.001
	Multiple sclerosis	5	2013 - 2017	-1.74 (-2.31, -1.16)	<0.001	5	2013 - 2017	-2.19 (-2.70, -1.68)	<0.001	5	2013 - 2017	-1.22 (-1.88, -0.56)	0.001
	Multiple sclerosis	6	2017 - 2021	-2.33 (-2.70, -1.95)	<0.001	6	2017 - 2021	-2.77 (-3.11, -2.43)	<0.001	6	2017 - 2021	-1.98 (-2.40, -1.56)	<0.001
	Multiple sclerosis	AAPC	1990 - 2021	-1.96 (-2.11, -1.81)	<0.001	AAPC	1990 - 2021	-2.03 (-2.15, -1.92)	<0.001	AAPC	1990 - 2021	-1.92 (-2.08, -1.75)	<0.001
High SDI	Multiple sclerosis	1	1990 - 1993	-1.72 (-2.16, -1.27)	<0.001	1	1990 - 1993	-1.36 (-1.90, -0.82)	<0.001	1	1990 - 1993	-2.11 (-2.56, -1.66)	<0.001
	Multiple sclerosis	2	1993 - 2002	-1.08 (-1.17, -0.98)	<0.001	2	1993 - 2002	-0.79 (-0.91, -0.67)	<0.001	2	1993 - 2002	-1.45 (-1.56, -1.35)	<0.001
	Multiple sclerosis	3	2002 - 2013	-1.85 (-1.92, -1.78)	<0.001	3	2002 - 2013	-1.75 (-1.84, -1.67)	<0.001	3	2002 - 2013	-1.96 (-2.04, -1.89)	<0.001
	Multiple sclerosis	4	2013 - 2018	-1.08 (-1.38, -0.77)	<0.001	4	2013 - 2021	-1.36 (-1.49, -1.24)	<0.001	4	2013 - 2018	-0.86 (-1.17, -0.55)	<0.001
	Multiple sclerosis	5	2018 - 2021	-1.70 (-2.20, -1.19)	<0.001	5	...	...	...	5	2018 - 2021	-1.69 (-2.21, -1.18)	<0.001
	Multiple sclerosis	6	...	...	...	6	...	...	...	6	...	...	...
	Multiple sclerosis	AAPC	1990 - 2021	-1.47 (-1.56, -1.39)	<0.001	AAPC	1990 - 2021	-1.34 (-1.41, -1.26)	<0.001	AAPC	1990 - 2021	-1.63 (-1.71, -1.54)	<0.001

	Multiple sclerosis	1	1990 - 1994	1.93 (1.10, 2.77)	<0.001	1	1990 - 1994	0.68 (-0.09, 1.46)	0.081	1	1990 - 1994	2.44 (1.71, 3.19)	<0.001
	Multiple sclerosis	2	1994 - 1997	-3.03 (-5.42, -0.59)	0.018	2	1994 - 1997	-2.23 (-4.49, 0.09)	0.058	2	1994 - 1997	-3.77 (-5.83, -1.67)	0.002
	Multiple sclerosis	3	1997 - 2005	-1.49 (-1.83, -1.14)	<0.001	3	1997 - 2005	-0.03 (-0.36, 0.29)	0.825	3	1997 - 2000	-1.40 (-3.65, 0.90)	0.211
High-middle SDI	Multiple sclerosis	4	2005 - 2012	-3.45 (-3.90, -2.99)	<0.001	4	2005 - 2021	-2.10 (-2.20, -2.00)	<0.001	4	2000 - 2005	-2.55 (-3.25, -1.84)	<0.001
	Multiple sclerosis	5	2012 - 2021	-1.60 (-1.87, -1.33)	<0.001	5	...	...	...	5	2005 - 2012	-4.13 (-4.53, -3.73)	<0.001
	Multiple sclerosis	6	...	...	...	6	...	...	...	6	2012 - 2021	-1.36 (-1.60, -1.12)	<0.001
	Multiple sclerosis	AAPC	1990 - 2021	-1.68 (-1.97, -1.40)	<0.001	AAPC	1990 - 2021	-1.23 (-1.47, -0.98)	<0.001	AAPC	1990 - 2021	-1.94 (-2.27, -1.62)	<0.001
	Multiple sclerosis	1	1990 - 1994	0.27 (0.02, 0.52)	0.037	1	1990 - 1997	0.94 (0.79, 1.09)	<0.001	1	1990 - 1994	-0.02 (-0.25, 0.22)	0.886
	Multiple sclerosis	2	1994 - 1997	0.97 (0.24, 1.71)	0.012	2	1997 - 2004	-0.75 (-0.93, -0.57)	<0.001	2	1994 - 1997	1.13 (0.42, 1.84)	0.003
Middle SDI	Multiple sclerosis	3	1997 - 2005	-0.13 (-0.22, -0.04)	0.009	3	2004 - 2008	-1.76 (-2.29, -1.22)	<0.001	3	1997 - 2021	0.16 (0.15, 0.18)	<0.001
	Multiple sclerosis	4	2005 - 2011	-0.41 (-0.56, -0.26)	<0.001	4	2008 - 2018	-1.31 (-1.41, -1.21)	<0.001	4	...	...	...
	Multiple sclerosis	5	2011 - 2021	-0.14 (-0.19, -0.08)	<0.001	5	2018 - 2021	0.02 (-0.57, 0.62)	0.941	5	...	...	...
	Multiple sclerosis	6	...	...	...	6	...	...	...	6	...	...	...

	Multiple sclerosis	AAPC	1990 - 2021	-0.03 (-0.11, 0.05)	0.471	AAPC	1990 - 2021	-0.61 (-0.71, -0.51)	<0.001	AAPC	1990 - 2021	0.23 (0.16, 0.30)	<0.001
	Multiple sclerosis	1	1990 - 2005	0.16 (0.15, 0.18)	<0.001	1	1990 - 1996	0.33 (0.21, 0.44)	<0.001	1	1990 - 1999	0.19 (0.16, 0.22)	<0.001
	Multiple sclerosis	2	2005 - 2008	0.60 (0.21, 0.99)	0. 005	2	1996 - 2000	-0.10 (-0.44, 0.23)	0.517	2	1999 - 2005	0.39 (0.31, 0.46)	<0.001
	Multiple sclerosis	3	2008 - 2015	0.08 (0.01, 0.14)	0. 020	3	2000 - 2003	0.34 (-0.30, 0.98)	0.274	3	2005 - 2008	1.04 (0.71, 1.37)	<0.001
Low-middle SDI	Multiple sclerosis	4	2015 - 2018	0.50 (0.11, 0.89)	0. 014	4	2003 - 2006	-0.86 (-1.48, -0.23)	0.011	4	2008 - 2015	0.31 (0.26, 0.37)	<0.001
	Multiple sclerosis	5	2018 - 2021	-0.02 (-0.21, 0.18)	0. 870	5	2006 - 2015	-0.45 (-0.52, -0.39)	<0.001	5	2015 - 2018	0.65 (0.32, 0.98)	0.001
	Multiple sclerosis	6	...	...	...	6	2015 - 2021	-0.12 (-0.23, -0.01)	0.041	6	2018 - 2021	0.12 (-0.04, 0.28)	0.144
	Multiple sclerosis	AAPC	1990 - 2021	0.20 (0.15, 0.26)	<0.001	AAPC	1990 - 2021	-0.16 (-0.25, -0.06)	0.001	AAPC	1990 - 2021	0.38 (0.33, 0.42)	<0.001
	Multiple sclerosis	1	1990 - 1995	0.46 (0.39, 0.54)	<0.001	1	1990 - 1995	0.92 (0.80, 1.04)	<0.001	1	1990 - 2005	0.30 (0.28, 0.32)	<0.001
	Multiple sclerosis	2	1995 - 2005	0.36 (0.33, 0.39)	<0.001	2	1995 - 2005	0.65 (0.60, 0.69)	<0.001	2	2005 - 2012	0.15 (0.09, 0.21)	<0.001
Low SDI	Multiple sclerosis	3	2005 - 2012	0.02 (-0.03, 0.07)	0.345	3	2005 - 2011	-0.23 (-0.34, -0.12)	<0.001	3	2012 - 2018	0.36 (0.28, 0.45)	<0.001
	Multiple sclerosis	4	2012 - 2018	0.27 (0.20, 0.33)	<0.001	4	2011 - 2021	0.18 (0.14, 0.21)	<0.001	4	2018 - 2021	0.65 (0.47, 0.83)	<0.001
	Multiple sclerosis	5	2018 - 2021	0.47 (0.33, 0.62)	<0.001	5	...	...	...	5	...	...	...

	Multiple sclerosis	6	...	...	...	6	...	...	...	6	...	...	...
	Multiple sclerosis	AAPC	1990 - 2021	0.29 (0.27, 0.32)	<0.001	AAPC	1990 - 2021	0.37 (0.34, 0.40)	<0.001	AAPC	1990 - 2021	0.31 (0.28, 0.34)	<0.001
	Parkinson's disease	1	1990 - 1995	-0.24 (-0.35, -0.12)	0.001	1	1990 - 1996	-0.49 (-0.61, -0.38)	<0.001	1	1990 - 1995	-0.40 (-0.52, -0.28)	<0.001
	Parkinson's disease	2	1995 - 1999	-0.81 (-1.06, -0.55)	<0.001	2	1996 - 2003	-0.87 (-0.99, -0.76)	<0.001	2	1995 - 1999	-0.94 (-1.21, -0.67)	<0.001
	Parkinson's disease	3	1999 - 2004	-0.20 (-0.37, -0.04)	0.021	3	2003 - 2014	-1.51 (-1.57, -1.46)	<0.001	3	1999 - 2004	-0.22 (-0.40, -0.05)	0.016
Global	Parkinson's disease	4	2004 - 2007	-1.01 (-1.54, -0.48)	0.001	4	2014 - 2017	-0.87 (-1.63, -0.11)	0.027	4	2004 - 2007	-1.11 (-1.66, -0.56)	0.001
	Parkinson's disease	5	2007 - 2010	0.36 (-0.16, 0.89)	0.156	5	2017 - 2021	-1.65 (-1.88, -1.42)	<0.001	5	2007 - 2010	0.42 (-0.11, 0.96)	0.112
	Parkinson's disease	6	2010 - 2021	-0.54 (-0.58, -0.51)	<0.001	6	...	...	...	6	2010 - 2021	-0.56 (-0.60, -0.52)	<0.001
	Parkinson's disease	AAPC	1990 - 2021	-0.43 (-0.51, -0.35)	<0.001	AAPC	1990 - 2021	-1.13 (-1.21, -1.04)	<0.001	AAPC	1990 - 2021	-0.49 (-0.57, -0.41)	<0.001
	Parkinson's disease	1	1990 - 1993	-1.44 (-1.79, -1.09)	<0.001	1	1990 - 1995	-1.51 (-1.69, -1.32)	<0.001	1	1990 - 1993	-1.58 (-1.89, -1.26)	<0.001
High SDI	Parkinson's disease	2	1993 - 1998	-0.75 (-0.98, -0.53)	<0.001	2	1995 - 2003	-0.41 (-0.53, -0.29)	<0.001	2	1993 - 1998	-0.97 (-1.18, -0.75)	<0.001
	Parkinson's disease	3	1998 - 2003	-0.25 (-0.48, -0.01)	0.044	3	2003 - 2014	-0.90 (-0.98, -0.83)	<0.001	3	1998 - 2003	-0.62 (-0.84, -0.41)	<0.001
	Parkinson's	4	2003 -	-1.11 (-1.17,	<0.001	4	2014 -	0.12 (-0.86,	0.796	4	2003 -	-1.56 (-1.61,	<0.001

	disease		2014	-1.05)			2017	1.11)			2014	-1.50)	
	Parkinson's disease	5	2014 - 2018	-0.32 (-0.72, 0.07)	0.104	5	2017 - 2021	-1.50 (-1.81, -1.19)	<0.001	5	2014 - 2018	-0.65 (-1.01, -0.28)	0.002
	Parkinson's disease	6	2018 - 2021	-1.28 (-1.67, -0.88)	<0.001	6	...	...	...	6	2018 - 2021	-1.34 (-1.70, -0.98)	<0.001
	Parkinson's disease	AAPC	1990 - 2021	-0.86 (-0.94, -0.78)	<0.001	AAPC	1990 - 2021	-0.85 (-0.96, -0.75)	<0.001	AAPC	1990 - 2021	-1.17 (-1.25, -1.10)	<0.001
High-middle SDI	Parkinson's disease	1	1990 - 1995	0.43 (0.27, 0.59)	<0.001	1	1990 - 1995	0.93 (0.71, 1.14)	<0.001	1	1990 - 1995	0.02 (-0.15, 0.18)	0.835
	Parkinson's disease	2	1995 - 1998	-0.79 (-1.48, -0.09)	0.030	2	1995 - 2004	-0.30 (-0.40, -0.20)	<0.001	2	1995 - 1998	-1.20 (-1.90, -0.49)	0.003
	Parkinson's disease	3	1998 - 2004	0.46 (0.30, 0.62)	<0.001	3	2004 - 2007	-1.50 (-2.40, -0.59)	0.003	3	1998 - 2004	0.33 (0.16, 0.49)	0.001
	Parkinson's disease	4	2004 - 2007	-0.64 (-1.34, 0.07)	0.075	4	2007 - 2010	-0.64 (-1.54, 0.28)	0.158	4	2004 - 2007	-0.89 (-1.62, -0.16)	0.021
	Parkinson's disease	5	2007 - 2011	0.66 (0.30, 1.02)	0.001	5	2010 - 2014	-1.66 (-2.12, -1.19)	<0.001	5	2007 - 2011	0.42 (0.06, 0.79)	0.025
	Parkinson's disease	6	2011 - 2021	-0.50 (-0.56, -0.45)	<0.001	6	2014 - 2021	-1.28 (-1.42, -1.15)	<0.001	6	2011 - 2021	-0.64 (-0.69, -0.58)	<0.001
	Parkinson's disease	AAPC	1990 - 2021	-0.06 (-0.16, 0.05)	0.293	AAPC	1990 - 2021	-0.65 (-0.79, -0.52)	<0.001	AAPC	1990 - 2021	-0.29 (-0.40, -0.18)	<0.001
Middle SDI	Parkinson's disease	1	1990 - 1996	-0.20 (-0.35, -0.05)	0.013	1	1990 - 1995	0.66 (0.45, 0.88)	<0.001	1	1990 - 1996	-0.33 (-0.52, -0.15)	0.002
	Parkinson's disease	2	1996 - 1999	-1.64 (-2.52, -0.74)	0.001	2	1995 - 2002	-1.98 (-2.14, -1.83)	<0.001	2	1996 - 1999	-1.49 (-2.58, -0.39)	0.012
	Parkinson's	3	1999 -	-0.55 (-0.82,	0.001	3	2002 -	-3.04 (-3.32,	<0.001	3	1999 -	-0.31 (-0.65,	0.073

	disease		2004	-0.27)			2007	-2.75)			2004	0.03)	
	Parkinson's disease	4	2004 - 2007	-1.44 (-2.30, -0.58)	0.003	4	2007 - 2015	-2.49 (-2.62, -2.36)	<0.001	4	2004 - 2007	-1.34 (-2.39, -0.27)	0.017
	Parkinson's disease	5	2007 - 2010	1.63 (0.77, 2.51)	0.001	5	2015 - 2021	-1.12 (-1.30, -0.94)	<0.001	5	2007 - 2010	1.99 (0.93, 3.06)	0.001
	Parkinson's disease	6	2010 - 2021	-0.64 (-0.70, -0.58)	<0.001	6	...	...	...	6	2010 - 2021	-0.53 (-0.60, -0.46)	<0.001
	Parkinson's disease	AAPC	1990 - 2021	-0.50 (-0.64, -0.35)	<0.001	AAPC	1990 - 2021	-1.70 (-1.77, -1.62)	<0.001	AAPC	1990 - 2021	-0.39 (-0.57, -0.21)	<0.001
	Parkinson's disease	1	1990 - 1997	-0.09 (-0.53, 0.36)	0.687	1	1990 - 1998	0.59 (0.33, 0.86)	<0.001	1	1990 - 2005	-0.52 (-0.68, -0.35)	<0.001
	Parkinson's disease	2	1997 - 2000	-1.87 (-5.03, 1.38)	0.243	2	1998 - 2001	-1.60 (-3.92, 0.77)	0.169	2	2005 - 2021	-0.01 (-0.16, 0.14)	0.880
	Parkinson's disease	3	2000 - 2021	-0.32 (-0.40, -0.24)	<0.001	3	2001 - 2007	0.50 (-0.04, 1.05)	0.067	3	...	...	...
Low-middle SDI	Parkinson's disease	4	...	...	...	4	2007 - 2010	-1.66 (-4.13, 0.87)	0.180	4	...	...	...
	Parkinson's disease	5	...	...	...	5	2010 - 2019	-0.62 (-0.89, -0.35)	<0.001	5	...	...	...
	Parkinson's disease	6	...	...	...	6	2019 - 2021	-3.47 (-5.91, -0.96)	0.010	6	...	...	...
	Parkinson's disease	AAPC	1990 - 2021	-0.42 (-0.74, -0.10)	0.009	AAPC	1990 - 2021	-0.48 (-0.84, -0.10)	0.012	AAPC	1990 - 2021	-0.26 (-0.36, -0.15)	<0.001
Low SDI	Parkinson's disease	1	1990 - 1995	0.45 (-0.03, 0.93)	0.063	1	1990 - 1995	1.32 (0.78, 1.86)	<0.001	1	1990 - 1995	0.24 (-0.28, 0.76)	0.341
	Parkinson's	2	1995 -	-1.56 (-2.24,	<0.001	2	1995 -	-2.12 (-3.24,	0.001	2	1995 -	-1.54 (-2.06,	<0.001

disease		2000	-0.88)			1999	-0.99)			2001	-1.03)	
Parkinson's disease	3	2000 - 2011	-0.67 (-0.84, -0.50)	<0.001	3	1999 - 2003	2.17 (1.03, 3.32)	0.001	3	2001 - 2012	-0.62 (-0.81, -0.43)	<0.001
Parkinson's disease	4	2011 - 2014	2.08 (-0.23, 4.43)	0.075	4	2003 - 2010	-0.82 (-1.21, -0.42)	0.001	4	2012 - 2015	1.66 (-0.78, 4.16)	0.171
Parkinson's disease	5	2014 - 2021	-1.48 (-1.77, -1.19)	<0.001	5	2010 - 2013	6.15 (3.67, 8.69)	<0.001	5	2015 - 2021	-1.20 (-1.58, -0.81)	<0.001
Parkinson's disease	6	...	...	...	6	2013 - 2021	-2.72 (-2.98, -2.45)	<0.001	6	...	...	...
Parkinson's disease	AAPC	1990 - 2021	-0.56 (-0.81, -0.30)	<0.001	AAPC	1990 - 2021	-0.11 (-0.42, 0.21)	0.502	AAPC	1990 - 2021	-0.56 (-0.82, -0.29)	<0.001

Supplementary Table S4. Decomposition analysis of DALYs at global and six regional levels from 1990 to 2021.

Disease	Sex	Location	Overall different	Aging	Population growth	Epidemiologic changes
Neurological disorders	Both	Low SDI	8531.59	-22.143(-0.26%)	10241.246(120.04%)	-1687.509(-19.78%)
Neurological disorders	Both	Low-middle SDI	41355.41	11630.792(28.12%)	43254.889(104.59%)	-13530.274(-32.72%)
Neurological disorders	Both	Middle SDI	136272.09	65171.026(47.82%)	118472.878(86.94%)	-47371.81(-34.76%)
Neurological disorders	Both	High-middle SDI	122202.42	54422.206(44.53%)	89200.076(72.99%)	-21419.865(-17.53%)
Neurological disorders	Both	High SDI	79336.99	98808.726(124.54%)	129253.713(162.92%)	-148725.452(-187.46%)
Neurological disorders	Both	Global	387780.76	193444.055(49.88%)	469089.577(120.97%)	-274752.874(-70.85%)
Neurological disorders	Female	Low SDI	4027.82	113.016(2.81%)	4154.213(103.14%)	-239.412(-5.94%)
Neurological disorders	Female	Low-middle SDI	13510.58	4033.449(29.85%)	13959.185(103.32%)	-4482.051(-33.17%)
Neurological disorders	Female	Middle SDI	33510.53	19809.239(59.11%)	39538.72(117.99%)	-25837.425(-77.1%)
Neurological disorders	Female	High-middle SDI	44189.74	19845.16(44.91%)	35613.751(80.59%)	-11269.175(-25.5%)
Neurological disorders	Female	High SDI	38204.76	47734.397(124.94%)	69088.303(180.84%)	-78617.941(-205.78%)
Neurological disorders	Female	Global	133494.21	76118.046(57.02%)	216718.123(162.34%)	-159341.962(-119.36%)

Neurological disorders	Male	Low SDI	4503.78	-190.654(-4.23%)	6027.717(133.84%)	-1333.286(-29.6%)
Neurological disorders	Male	Low-middle SDI	27844.82	6304.804(22.64%)	28505.556(102.37%)	-6965.536(-25.02%)
Neurological disorders	Male	Middle SDI	102761.56	46371.707(45.13%)	77382.614(75.3%)	-20992.761(-20.43%)
Neurological disorders	Male	High-middle SDI	78012.68	39650.365(50.83%)	54156.032(69.42%)	-15793.717(-20.25%)
Neurological disorders	Male	High SDI	41132.23	55885.52(135.87%)	59610.808(144.92%)	-74364.1(-180.79%)
Neurological disorders	Male	Global	254286.55	128028.652(50.35%)	252453.461(99.28%)	-126195.563(-49.63%)
Alzheimer's disease and other dementias	Both	Low SDI	14856.96	-88.398(-0.59%)	19041.394(128.16%)	-4096.033(-27.57%)
Alzheimer's disease and other dementias	Both	Low-middle SDI	81689.64	14769.717(18.08%)	89804.976(109.93%)	-22885.05(-28.01%)
Alzheimer's disease and other dementias	Both	Middle SDI	274892.34	72207.537(26.27%)	279899.793(101.82%)	-77214.989(-28.09%)
Alzheimer's disease and other dementias	Both	High-middle SDI	234297.49	54582.106(23.3%)	189861.943(81.03%)	-10146.562(-4.33%)
Alzheimer's disease and other dementias	Both	High SDI	132249.24	95194.179(71.98%)	188922.986(142.85%)	-151867.93(-114.83%)

Alzheimer's disease and other dementias	Both	Global	738298.34	192077.007(26.02%)	828148.928(112.17%)	-281927.598(-38.19%)
Alzheimer's disease and other dementias	Female	Low SDI	5247.06	130.199(2.48%)	5483.695(104.51%)	-366.832(-6.99%)
Alzheimer's disease and other dementias	Female	Low-middle SDI	17434.48	3703.336(21.24%)	19140.549(109.79%)	-5409.405(-31.03%)
Alzheimer's disease and other dementias	Female	Middle SDI	38850.65	15206.95(39.14%)	56228.318(144.73%)	-32584.617(-83.87%)
Alzheimer's disease and other dementias	Female	High-middle SDI	51042.93	14867.29(29.13%)	47512.332(93.08%)	-11336.692(-22.21%)
Alzheimer's disease and other dementias	Female	High SDI	48189.66	38710.198(80.33%)	80806.15(167.68%)	-71326.69(-148.01%)
Alzheimer's disease and other dementias	Female	Global	160881.04	57198.785(35.55%)	254103.25(157.94%)	-150420.996(-93.5%)
Alzheimer's disease and other dementias	Male	Low SDI	9609.9	-344.488(-3.58%)	13257.016(137.95%)	-3302.628(-34.37%)
Alzheimer's disease and other dementias	Male	Low-middle SDI	64255.16	9059.188(14.1%)	67589.796(105.19%)	-12393.822(-19.29%)
Alzheimer's disease and other dementias	Male	Middle SDI	236041.69	63123.039(26.74%)	217352.093(92.08%)	-44433.441(-18.82%)

Alzheimer's disease and other dementias	Male	High-middle SDI	183254.56	53991.367(29.46%)	145988.347(79.66%)	-16725.158(-9.13%)
Alzheimer's disease and other dementias	Male	High SDI	84059.58	69383.967(82.54%)	110922.618(131.96%)	-96247.008(-114.5%)
Alzheimer's disease and other dementias	Male	Global	577417.3	167764.777(29.05%)	573744.835(99.36%)	-164092.313(-28.42%)
Multiple sclerosis	Both	Global	5755.48	2938.369(51.05%)	73815.843(1282.53%)	-70998.737(-1233.58%)
Multiple sclerosis	Both	High SDI	-1897.12	454.171(-23.94%)	29460.669(-1552.92%)	-31811.957(1676.86%)
Multiple sclerosis	Both	High-middle SDI	-585.84	-37.609(6.42%)	13471.174(-2299.46%)	-14019.403(2393.04%)
Multiple sclerosis	Both	Middle SDI	5397.3	229.58(4.25%)	5170.6(95.8%)	-2.878(-0.05%)
Multiple sclerosis	Both	Low-middle SDI	2368.62	21.718(0.92%)	2178.487(91.97%)	168.412(7.11%)
Multiple sclerosis	Both	Low SDI	572.5	-1.921(-0.34%)	520.703(90.95%)	53.717(9.38%)
Multiple sclerosis	Female	Low SDI	148.75	-0.808(-0.54%)	133.123(89.49%)	16.432(11.05%)
Multiple sclerosis	Female	Low-middle SDI	401.42	11.706(2.92%)	423.654(105.54%)	-33.943(-8.46%)
Multiple sclerosis	Female	Middle SDI	1120.33	86.069(7.68%)	1378.661(123.06%)	-344.405(-30.74%)
Multiple sclerosis	Female	High-middle SDI	926.58	-85.637(-9.24%)	4341.731(468.58%)	-3329.518(-359.33%)

Multiple sclerosis	Female	High SDI	-335.47	-144.449(43.06%)	14974.142(-4463.63%)	-15165.167(4520.57%)
Multiple sclerosis	Female	Global	2224.4	1303.86(58.62%)	34786.259(1563.85%)	-33865.716(-1522.47%)
Multiple sclerosis	Male	Low SDI	423.75	-0.751(-0.18%)	381.451(90.02%)	43.052(10.16%)
Multiple sclerosis	Male	Low-middle SDI	1967.2	10.418(0.53%)	1698.668(86.35%)	258.113(13.12%)
Multiple sclerosis	Male	Middle SDI	4276.98	153.093(3.58%)	3700.379(86.52%)	423.506(9.9%)
Multiple sclerosis	Male	High-middle SDI	-1512.41	142.762(-9.44%)	9255.726(-611.99%)	-10910.901(721.42%)
Multiple sclerosis	Male	High SDI	-1561.64	657.297(-42.09%)	14390.419(-921.49%)	-16609.36(1063.58%)
Multiple sclerosis	Male	Global	3531.07	1715.301(48.58%)	38983.115(1104%)	-37167.343(-1052.58%)
Parkinson's disease	Both	Low SDI	-6897.87	357.4(-5.18%)	-9616.987(139.42%)	2361.719(-34.24%)
Parkinson's disease	Both	Low-middle SDI	-42702.85	-8385.447(19.64%)	-43396.963(101.63%)	9079.558(-21.26%)
Parkinson's disease	Both	Middle SDI	-144017.55	-48554.184(33.71%)	-124447.223(86.41%)	28983.859(-20.13%)
Parkinson's disease	Both	High-middle SDI	-111509.23	-34593.745(31.02%)	-79631.086(71.41%)	2715.6(-2.44%)
Parkinson's disease	Both	High SDI	-51015.13	-33036.835(64.76%)	-52660.96(103.23%)	34682.665(-67.99%)
Parkinson's disease	Both	Global	-356273.05	-98404.198(27.62%)	-334353.924(93.85%)	76485.068(-21.47%)

Parkinson's disease	Female	Low SDI	-1367.99	12.376(-0.9%)	-1491.567(109.03%)	111.199(-8.13%)
Parkinson's disease	Female	Low-middle SDI	-4325.31	-988.236(22.85%)	-4266.998(98.65%)	929.92(-21.5%)
Parkinson's disease	Female	Middle SDI	-6460.44	-3683.099(57.01%)	-9522.761(147.4%)	6745.418(-104.41%)
Parkinson's disease	Female	High-middle SDI	-7779.77	-3182.527(40.91%)	-7944.248(102.11%)	3347.004(-43.02%)
Parkinson's disease	Female	High SDI	-9649.42	-6133.044(63.56%)	-11283.979(116.94%)	7767.599(-80.5%)
Parkinson's disease	Female	Global	-29611.23	-10972.579(37.06%)	-42679.7(144.13%)	24041.046(-81.19%)
Parkinson's disease	Male	Low SDI	-5529.88	489.092(-8.84%)	-7954.153(143.84%)	1935.185(-35%)
Parkinson's disease	Male	Low-middle SDI	-38377.54	-5925.164(15.44%)	-37577.749(97.92%)	5125.375(-13.36%)
Parkinson's disease	Male	Middle SDI	-137557.11	-48927.37(35.57%)	-111149.641(80.8%)	22519.904(-16.37%)
Parkinson's disease	Male	High-middle SDI	-103729.46	-42061.788(40.55%)	-73441.497(70.8%)	11773.823(-11.35%)
Parkinson's disease	Male	High SDI	-41365.7	-35869.464(86.71%)	-43900.143(106.13%)	38403.902(-92.84%)
Parkinson's disease	Male	Global	-326661.82	-110524.942(33.83%)	-290281.491(88.86%)	74144.613(-22.7%)

Supplementary Table S5. Frontier analysis based on SDI and DALYs in 204 countries and territories.

Location	Disease	SDI	Age-standardized DALY rate	Frontier DALYs	Effective difference	Effective difference rank (Age-standardized DALY rank)
Afghanistan	Neurological disorders	0.337199998	6.84763487	0.470799551	6.37683532	98(98)
Albania	Neurological disorders	0.706849791	24.9500604	0.470923839	24.47913657	202(202)
Algeria	Neurological disorders	0.659500924	20.2686355	0.470905446	19.79773005	196(196)
American Samoa	Neurological disorders	0.723727533	6.328444971	0.472511785	5.855933186	90(90)
Andorra	Neurological disorders	0.869444113	16.90871125	0.471124388	16.43758686	179(179)
Angola	Neurological disorders	0.453721949	6.252156419	0.472080835	5.780075584	89(89)
Antigua and Barbuda	Neurological disorders	0.749886887	2.884969288	0.471287174	2.413682113	35(34)
Argentina	Neurological disorders	0.723122973	8.176261409	0.470811812	7.705449596	116(116)
Armenia	Neurological disorders	0.701833194	13.59663497	0.470805124	13.12582985	160(160)
Australia	Neurological disorders	0.844252814	10.62584614	0.470926483	10.15491965	136(136)
Austria	Neurological disorders	0.853837004	18.89293299	0.471101031	18.42183196	190(190)
Azerbaijan	Neurological disorders	0.694851274	10.84280376	0.471045897	10.37175786	140(140)
Bahamas	Neurological disorders	0.805020668	3.7331526	0.471450529	3.261702071	46(45)
Bahrain	Neurological disorders	0.753043204	11.50373474	0.470806788	11.03292795	146(146)
Bangladesh	Neurological disorders	0.492420885	6.884780893	0.471391492	6.4133894	100(100)
Barbados	Neurological disorders	0.746748764	3.415413432	0.470798436	2.944614996	44(43)
Belarus	Neurological disorders	0.784484711	7.546984218	0.475613111	7.071371107	108(108)
Belgium	Neurological disorders	0.853654016	20.9869505	0.470881405	20.51606909	197(197)
Belize	Neurological disorders	0.610229002	4.125018871	0.473716467	3.651302404	52(52)
Benin	Neurological disorders	0.373486574	1.202793036	0.470828533	0.731964504	11(9)
Bermuda	Neurological disorders	0.821365422	6.666497005	0.470830539	6.195666466	94(94)

Bhutan	Neurological disorders	0.473062378	4.47360009	0.470918823	4.002681267	61(61)
Bolivia (Plurinational State of)	Neurological disorders	0.599010799	0.921213745	0.470819838	0.450393907	7(5)
Bosnia and Herzegovina	Neurological disorders	0.723077893	15.68532792	0.4710835	15.21424442	173(173)
Botswana	Neurological disorders	0.642721629	8.076563189	0.472426736	7.604136453	113(113)
Brazil	Neurological disorders	0.653043887	16.33092385	0.471025689	15.85989816	175(175)
Brunei Darussalam	Neurological disorders	0.810234367	6.965121339	0.470964037	6.494157302	103(103)
Bulgaria	Neurological disorders	0.768150939	10.95496352	0.471653369	10.48331015	141(141)
Burkina Faso	Neurological disorders	0.285118402	1.02898505	0.478119443	0.550865607	8(6)
Burundi	Neurological disorders	0.289374365	2.321814973	0.470940925	1.850874048	29(28)
Cabo Verde	Neurological disorders	0.533534539	0.864330831	0.470853559	0.393477271	5(4)
Cambodia	Neurological disorders	0.473621491	14.03113739	0.470882719	13.56025467	164(164)
Cameroon	Neurological disorders	0.479691223	1.429597794	0.470879835	0.958717959	13(11)
Canada	Neurological disorders	0.87317068	19.95458903	0.471589178	19.48299985	195(195)
Central African Republic	Neurological disorders	0.30916769	2.736029555	0.472397754	2.263631801	34(33)
Chad	Neurological disorders	0.240436019	2.144338443	0.540727371	1.603611072	24(24)
Chile	Neurological disorders	0.771514716	4.374580972	0.4726441	3.901936871	59(59)
China	Neurological disorders	0.72162976	15.03847842	0.473434555	14.56504386	168(168)
Colombia	Neurological disorders	0.655442913	4.062924599	0.473672271	3.589252328	51(51)
Comoros	Neurological disorders	0.475978688	5.778382256	0.470932199	5.307450057	82(82)
Congo	Neurological disorders	0.583075236	5.989761581	0.470958951	5.51880263	84(84)
Cook Islands	Neurological disorders	0.779109955	6.764166754	0.471226477	6.292940278	96(96)
Costa Rica	Neurological disorders	0.700340477	6.364096431	0.471342306	5.892754126	91(91)
Croatia	Neurological disorders	0.798341027	17.36656746	0.471279982	16.89528748	182(182)
Cuba	Neurological disorders	0.668729864	8.013668951	0.47093017	7.542738782	111(111)
Cyprus	Neurological disorders	0.835630545	15.3400317	0.470896529	14.86913517	171(171)
Czechia	Neurological disorders	0.828450433	14.95370843	0.470828533	14.4828799	167(167)

Côte d'Ivoire	Neurological disorders	0.425941883	1.658742225	0.471076272	1.187665953	19(17)
Democratic People's Republic of Korea	Neurological disorders	0.569854634	3.309227706	0.475042055	2.834185651	41(40)
Democratic Republic of the Congo	Neurological disorders	0.383179849	2.267161326	0.471526335	1.795634991	27(26)
Denmark	Neurological disorders	0.896424204	27.43737723	0.471026453	26.96635078	203(203)
Djibouti	Neurological disorders	0.487958371	8.970332107	0.471595812	8.498736295	120(120)
Dominica	Neurological disorders	0.746967185	1.466956829	0.478060707	0.988896122	15(13)
Dominican Republic	Neurological disorders	0.619388201	10.69804989	0.4718483	10.22620159	137(137)
Ecuador	Neurological disorders	0.661017053	1.696527896	0.47089781	1.225630086	21(20)
Egypt	Neurological disorders	0.606787094	18.24989403	0.47129503	17.778599	186(186)
El Salvador	Neurological disorders	0.563775188	3.228312799	0.471092427	2.757220372	39(38)
Equatorial Guinea	Neurological disorders	0.657857456	3.475553343	0.470813484	3.004739858	45(44)
Eritrea	Neurological disorders	0.403863943	1.481778734	0.470918823	1.010859912	16(14)
Estonia	Neurological disorders	0.844917787	10.59608028	0.470829437	10.12525084	135(135)
Eswatini	Neurological disorders	0.585459713	4.655285912	0.476004367	4.179281545	65(65)
Ethiopia	Neurological disorders	0.358823295	2.214968324	0.470878694	1.74408963	26(25)
Fiji	Neurological disorders	0.675051631	3.305545424	0.48597395	2.819571474	40(39)
Finland	Neurological disorders	0.859831368	12.06087173	0.480635616	11.58023611	148(148)
France	Neurological disorders	0.838364875	11.96079056	0.470983174	11.48980739	147(147)
Gabon	Neurological disorders	0.634691393	2.989115749	0.47228784	2.516827908	38(37)
Gambia	Neurological disorders	0.40971416	1.668628455	0.472479772	1.196148683	20(18)
Georgia	Neurological disorders	0.732473604	9.247828681	0.476532729	8.771295952	125(125)
Germany	Neurological disorders	0.902957091	18.66507645	0.470823517	18.19425293	189(189)
Ghana	Neurological disorders	0.56493039	2.429866483	0.470798436	1.959068047	30(29)
Greece	Neurological disorders	0.791854408	18.08738787	0.470898758	17.61648911	185(185)
Greenland	Neurological disorders	0.826210336	16.81672959	0.471119466	16.34561013	178(178)

Grenada	Neurological disorders	0.668993028	1.840047229	0.471976461	1.368070768	23(22)
Guam	Neurological disorders	0.803982203	6.45097057	0.470945592	5.980024978	92(92)
Guatemala	Neurological disorders	0.539972424	4.701548228	0.470926957	4.230621271	67(67)
Guinea	Neurological disorders	0.336401293	2.093726607	0.471236058	1.622490549	25(23)
Guinea-Bissau	Neurological disorders	0.353109621	0.736501942	0.471105013	0.265396929	3(2)
Guyana	Neurological disorders	0.650812335	2.287106023	0.47139645	1.815709573	28(27)
Haiti	Neurological disorders	0.448278285	1.197458742	0.470965496	0.726493246	10(8)
Honduras	Neurological disorders	0.513037248	2.985305477	0.471055929	2.514249548	37(36)
Hungary	Neurological disorders	0.790754768	11.24142864	0.470814866	10.77061377	145(145)
Iceland	Neurological disorders	0.87636168	19.49589753	0.473292442	19.02260509	192(192)
India	Neurological disorders	0.575401649	5.563832697	0.471039209	5.092793488	78(78)
Indonesia	Neurological disorders	0.656868336	9.338394066	0.472921911	8.865472155	126(126)
Iran (Islamic Republic of)	Neurological disorders	0.697207398	12.086565	0.470831455	11.61573355	150(150)
Iraq	Neurological disorders	0.662626231	13.3666724	0.471832867	12.89483953	158(158)
Ireland	Neurological disorders	0.87375385	19.72531078	0.471019145	19.25429163	194(194)
Israel	Neurological disorders	0.809011652	10.34963601	0.470979016	9.878656997	131(131)
Italy	Neurological disorders	0.805773534	15.47885131	0.471001278	15.00785003	172(172)
Jamaica	Neurological disorders	0.683263064	5.326274843	0.470798436	4.855476407	76(76)
Japan	Neurological disorders	0.871241813	11.23073445	0.470928539	10.75980591	144(144)
Jordan	Neurological disorders	0.725307227	22.81389004	0.47297842	22.34091162	200(200)
Kazakhstan	Neurological disorders	0.725144495	5.584348014	0.470991617	5.113356397	79(79)
Kenya	Neurological disorders	0.523768077	4.021549569	0.472279407	3.549270162	50(50)
Kiribati	Neurological disorders	0.527186583	15.07025337	0.470855978	14.5993974	169(169)
Kuwait	Neurological disorders	0.846651055	17.96979231	0.480568236	17.48922408	184(184)
Kyrgyzstan	Neurological disorders	0.603979328	12.97160628	0.471422752	12.50018353	155(155)
Lao People's Democratic Republic	Neurological disorders	0.489136091	10.12302996	0.471079338	9.651950623	129(129)

Latvia	Neurological disorders	0.830663516	8.513453988	0.470931363	8.042522625	119(119)
Lebanon	Neurological disorders	0.744746351	30.07028073	0.470988099	29.59929264	204(204)
Lesotho	Neurological disorders	0.510393066	10.75923979	0.472099919	10.28713987	139(139)
Liberia	Neurological disorders	0.352442452	1.235210121	0.471861676	0.763348445	12(10)
Libya	Neurological disorders	0.725771399	12.56645491	0.472472334	12.09398257	152(152)
Lithuania	Neurological disorders	0.856484049	9.177167647	0.470906624	8.706261023	124(124)
Luxembourg	Neurological disorders	0.884428955	11.16764366	0.473685987	10.69395767	143(143)
Madagascar	Neurological disorders	0.400246943	2.457665457	0.470858629	1.986806827	31(30)
Malawi	Neurological disorders	0.384553634	5.913677039	0.470958951	5.442718088	83(83)
Malaysia	Neurological disorders	0.742523828	9.119640412	0.472214587	8.647425825	122(122)
Maldives	Neurological disorders	0.650886627	13.86722832	0.471061454	13.39616687	162(162)
Mali	Neurological disorders	0.268579941	3.804202569	0.494860441	3.309342128	47(48)
Malta	Neurological disorders	0.801585034	10.25431671	0.470803118	9.783513592	130(130)
Marshall Islands	Neurological disorders	0.574091128	4.158543637	0.470821608	3.687722028	53(53)
Mauritania	Neurological disorders	0.4989451	1.757851702	0.476808524	1.281043178	22(21)
Mauritius	Neurological disorders	0.718260446	8.087906798	0.474019271	7.613887528	114(114)
Mexico	Neurological disorders	0.664575304	3.358647333	0.470798436	2.887848897	43(42)
Micronesia (Federated States of)	Neurological disorders	0.587534967	6.873326004	0.470878694	6.40244731	99(99)
Monaco	Neurological disorders	0.908262831	12.5774951	0.470878179	12.10661692	153(153)
Mongolia	Neurological disorders	0.617621565	9.828836059	0.477351437	9.351484622	127(127)
Montenegro	Neurological disorders	0.795800584	17.05548218	0.470818501	16.58466368	181(181)
Morocco	Neurological disorders	0.562698301	5.013067524	0.473871563	4.53919596	70(70)
Mozambique	Neurological disorders	0.326462614	4.312512483	0.471549499	3.840962983	57(57)
Myanmar	Neurological disorders	0.53390084	8.485778417	0.470868662	8.014909755	118(118)
Namibia	Neurological disorders	0.617564872	10.71050347	0.47118241	10.23932106	138(138)
Nauru	Neurological disorders	0.625177834	4.314738733	0.470813484	3.843925249	58(58)

Nepal	Neurological disorders	0.433174635	10.51706565	0.472363875	10.04470177	134(134)
Netherlands	Neurological disorders	0.888464256	21.67155597	0.472036439	21.19951953	198(198)
New Zealand	Neurological disorders	0.849442499	13.6718337	0.471356445	13.20047725	161(161)
Nicaragua	Neurological disorders	0.523958472	5.238791644	0.470958951	4.767832693	72(72)
Niger	Neurological disorders	0.168072774	1.688765352	1.265555018	0.423210334	6(19)
Nigeria	Neurological disorders	0.503390833	1.088035883	0.470902102	0.617133781	9(7)
Niue	Neurological disorders	0.72622205	4.712943539	0.472375661	4.240567877	68(68)
North Macedonia	Neurological disorders	0.750629703	16.50041958	0.472437437	16.02798214	176(176)
Northern Mariana Islands	Neurological disorders	0.771535213	5.774360875	0.471938346	5.302422529	81(81)
Norway	Neurological disorders	0.91613281	16.80289253	0.471108835	16.33178369	177(177)
Oman	Neurological disorders	0.773391602	5.627490781	0.471105947	5.156384835	80(80)
Pakistan	Neurological disorders	0.504028689	4.97447029	0.471580948	4.502889343	69(69)
Palau	Neurological disorders	0.754046931	6.091793586	0.470811812	5.620981773	85(85)
Palestine	Neurological disorders	0.631011665	14.80264994	0.470936455	14.33171348	165(165)
Panama	Neurological disorders	0.708864828	5.47844626	0.473605229	5.004841031	77(77)
Papua New Guinea	Neurological disorders	0.417797443	5.277897371	0.470944868	4.806952503	75(75)
Paraguay	Neurological disorders	0.635718099	17.69236945	0.471256392	17.22111305	183(183)
Peru	Neurological disorders	0.662054037	1.585141869	0.471101588	1.114040281	18(16)
Philippines	Neurological disorders	0.651219329	10.41003671	0.472617608	9.937419103	133(133)
Poland	Neurological disorders	0.812042809	13.07350535	0.470838565	12.60266679	157(157)
Portugal	Neurological disorders	0.744151851	6.178139871	0.480881678	5.697258194	87(88)
Puerto Rico	Neurological disorders	0.825525847	6.170931914	0.471150386	5.699781528	88(87)
Qatar	Neurological disorders	0.846860584	7.575529558	0.47083262	7.104696938	109(109)
Republic of Korea	Neurological disorders	0.886675267	14.9417516	0.480461226	14.46129037	166(166)
Republic of Moldova	Neurological disorders	0.732214875	8.187266857	0.470798436	7.716468421	117(117)
Romania	Neurological disorders	0.768453864	6.760720862	0.480564442	6.28015642	95(95)

Russian Federation	Neurological disorders	0.808536005	8.999271377	0.47109371	8.528177666	121(121)
Rwanda	Neurological disorders	0.435588706	19.56442735	0.470847838	19.09357951	193(193)
Saint Kitts and Nevis	Neurological disorders	0.754987055	0.680080242	0.470813484	0.209266758	2(1)
Saint Lucia	Neurological disorders	0.672509735	2.608010526	0.470920042	2.137090484	32(31)
Saint Vincent and the Grenadines	Neurological disorders	0.637195963	2.610452383	0.473045388	2.137406995	33(32)
Samoa	Neurological disorders	0.593392769	11.1208201	0.470798436	10.65002166	142(142)
San Marino	Neurological disorders	0.888005474	13.91439394	0.471870593	13.44252335	163(163)
Sao Tome and Principe	Neurological disorders	0.505413747	0.819519822	0.470904389	0.348615433	4(3)
Saudi Arabia	Neurological disorders	0.815143493	4.539994037	0.471775469	4.068218568	62(62)
Senegal	Neurological disorders	0.408054193	1.439450907	0.470826526	0.96862438	14(12)
Serbia	Neurological disorders	0.792416294	15.22681823	0.471219788	14.75559844	170(170)
Seychelles	Neurological disorders	0.730150775	3.797913153	0.470950448	3.326962706	48(47)
Sierra Leone	Neurological disorders	0.358665881	1.577943576	0.47162719	1.106316386	17(15)
Singapore	Neurological disorders	0.856097766	4.401588002	0.470857497	3.930730505	60(60)
Slovakia	Neurological disorders	0.81061053	9.832901286	0.47129056	9.361610726	128(128)
Slovenia	Neurological disorders	0.842430731	13.58791853	0.470838565	13.11707996	159(159)
Solomon Islands	Neurological disorders	0.429360316	8.149852335	0.470901433	7.678950901	115(115)
Somalia	Neurological disorders	0.077688109	3.768510524	3.768510524	0	1(46)
South Africa	Neurological disorders	0.679626598	5.216671747	0.471509371	4.745162376	71(71)
South Sudan	Neurological disorders	0.278371125	4.244859614	0.472308152	3.772551462	56(56)
Spain	Neurological disorders	0.769283698	9.137074049	0.470816247	8.666257802	123(123)
Sri Lanka	Neurological disorders	0.701534935	4.219684763	0.47116155	3.748523213	55(55)
Sudan	Neurological disorders	0.541949735	12.06886527	0.470833549	11.59803173	149(149)
Suriname	Neurological disorders	0.633665739	5.248025026	0.470820507	4.777204519	73(73)
Sweden	Neurological disorders	0.886880299	24.78490589	0.471064246	24.31384164	201(201)
Switzerland	Neurological disorders	0.933059111	19.45619181	0.470816271	18.98537554	191(191)

Syrian Arab Republic	Neurological disorders	0.623004075	13.01330272	0.470878694	12.54242403	156(156)
Taiwan (Province of China)	Neurological disorders	0.874747053	4.622656165	0.472563216	4.150092949	64(64)
Tajikistan	Neurological disorders	0.541511187	5.248990631	0.470932199	4.778058432	74(74)
Thailand	Neurological disorders	0.682547933	6.971071252	0.471144332	6.499926921	104(104)
Timor-Leste	Neurological disorders	0.444667619	10.40151947	0.470976486	9.930542984	132(132)
Togo	Neurological disorders	0.408533695	3.345200138	0.470850391	2.874349746	42(41)
Tokelau	Neurological disorders	0.686425621	7.170506368	0.471205353	6.699301015	105(105)
Tonga	Neurological disorders	0.626349936	12.31628068	0.472898163	11.84338252	151(151)
Trinidad and Tobago	Neurological disorders	0.768763254	4.203917056	0.47105702	3.732860036	54(54)
Tunisia	Neurological disorders	0.682432216	18.5938204	0.470989048	18.12283135	188(188)
Turkmenistan	Neurological disorders	0.682160776	6.603068119	0.470827975	6.132240144	93(93)
Tuvalu	Neurological disorders	0.576620529	6.93251453	0.470828533	6.461685997	102(102)
Türkiye	Neurological disorders	0.712692673	12.7238605	0.471330056	12.25253044	154(154)
Uganda	Neurological disorders	0.423261181	4.699373484	0.473200757	4.226172727	66(66)
Ukraine	Neurological disorders	0.760773913	7.927700204	0.470798436	7.456901767	110(110)
United Arab Emirates	Neurological disorders	0.849317734	16.94794061	0.474601019	16.47333959	180(180)
United Kingdom	Neurological disorders	0.859000182	18.43245085	0.470810326	17.96164053	187(187)
United Republic of Tanzania	Neurological disorders	0.446568273	6.808940725	0.471340175	6.33760055	97(97)
United States of America	Neurological disorders	0.862448354	22.6425775	0.470886921	22.17169058	199(199)
United States Virgin Islands	Neurological disorders	0.821830853	3.907716629	0.47198179	3.43573484	49(49)
Uruguay	Neurological disorders	0.719283445	7.330651196	0.470995286	6.85965591	106(106)
Uzbekistan	Neurological disorders	0.662621694	4.564949606	0.470820766	4.094128839	63(63)
Vanuatu	Neurological disorders	0.473100706	2.890893934	0.470949733	2.419944201	36(35)
Venezuela (Bolivarian Republic of)	Neurological disorders	0.596513059	6.106942186	0.470811812	5.636130374	86(86)
Viet Nam	Neurological disorders	0.627933721	6.927848865	0.470802895	6.45704597	101(101)

Yemen	Neurological disorders	0.450376375	15.92289553	0.471172972	15.45172256	174(174)
Zambia	Neurological disorders	0.505948954	7.541196034	0.471117826	7.070078208	107(107)
Zimbabwe	Neurological disorders	0.473819486	8.044535454	0.471449177	7.573086277	112(112)
Afghanistan	Alzheimer's disease and other dementias	0.337199998	10.73737031	1.91072084	8.826649467	91(91)
Albania	Alzheimer's disease and other dementias	0.706849791	30.53952329	1.912264479	28.62725881	202(202)
Algeria	Alzheimer's disease and other dementias	0.659500924	26.05295335	1.912010742	24.1409426	194(194)
American Samoa	Alzheimer's disease and other dementias	0.723727533	12.20715869	1.910902583	10.29625611	106(106)
Andorra	Alzheimer's disease and other dementias	0.869444113	17.53038062	1.91206477	15.61831585	153(153)
Angola	Alzheimer's disease and other dementias	0.453721949	9.2785227	1.912010742	7.366511958	75(75)
Antigua and Barbuda	Alzheimer's disease and other dementias	0.749886887	6.766545453	1.910714087	4.855831366	45(45)
Argentina	Alzheimer's disease and other dementias	0.723122973	11.88352055	1.910789581	9.972730972	101(101)
Armenia	Alzheimer's disease and other dementias	0.701833194	21.36647175	1.910786123	19.45568562	181(181)
Australia	Alzheimer's disease and other dementias	0.844252814	11.12621789	1.910854739	9.215363156	94(94)
Austria	Alzheimer's disease and other dementias	0.853837004	18.06989102	1.912115677	16.15777535	158(158)
Azerbaijan	Alzheimer's disease and other	0.694851274	17.31827026	1.912157377	15.40611288	149(149)

	dementias					
Bahamas	Alzheimer's disease and other dementias	0.805020668	6.861924092	1.912010742	4.949913349	46(46)
Bahrain	Alzheimer's disease and other dementias	0.753043204	18.32865031	1.910714087	16.41793622	160(160)
Bangladesh	Alzheimer's disease and other dementias	0.492420885	15.2941998	1.911318042	13.38288176	136(136)
Barbados	Alzheimer's disease and other dementias	0.746748764	5.639994146	1.910714087	3.729280059	31(30)
Belarus	Alzheimer's disease and other dementias	0.784484711	14.00517646	1.912731107	12.09244536	123(123)
Belgium	Alzheimer's disease and other dementias	0.853654016	20.510883	1.911038251	18.59984475	176(176)
Belize	Alzheimer's disease and other dementias	0.610229002	8.563842696	1.910714087	6.653128609	66(66)
Benin	Alzheimer's disease and other dementias	0.373486574	2.373844317	1.910714087	0.46313023	7(5)
Bermuda	Alzheimer's disease and other dementias	0.821365422	9.702849052	1.910876169	7.791972883	79(79)
Bhutan	Alzheimer's disease and other dementias	0.473062378	8.486333276	1.910822142	6.575511135	65(65)
Bolivia (Plurinational State of)	Alzheimer's disease and other dementias	0.599010799	5.678036069	1.910743343	3.767292726	32(31)
Bosnia and Herzegovina	Alzheimer's disease and other dementias	0.723077893	22.52393487	1.910930196	20.61300468	187(187)
Botswana	Alzheimer's disease and other	0.642721629	12.23911038	1.910810306	10.32830007	107(107)

	dementias					
Brazil	Alzheimer's disease and other dementias	0.653043887	20.70944907	1.910735185	18.79871388	177(177)
Brunei Darussalam	Alzheimer's disease and other dementias	0.810234367	12.54405302	1.912145769	10.63190725	111(111)
Bulgaria	Alzheimer's disease and other dementias	0.768150939	11.54299641	1.912036068	9.630960338	97(97)
Burkina Faso	Alzheimer's disease and other dementias	0.285118402	2.307288863	1.910714087	0.396574776	5(4)
Burundi	Alzheimer's disease and other dementias	0.289374365	4.034984712	1.912010742	2.12297397	20(19)
Cabo Verde	Alzheimer's disease and other dementias	0.533534539	2.288547565	1.910714087	0.377833479	4(3)
Cambodia	Alzheimer's disease and other dementias	0.473621491	26.05913809	1.912321724	24.14681636	195(195)
Cameroon	Alzheimer's disease and other dementias	0.479691223	3.259559031	1.910714087	1.348844945	14(13)
Canada	Alzheimer's disease and other dementias	0.87317068	19.87093327	1.910745734	17.96018754	168(168)
Central African Republic	Alzheimer's disease and other dementias	0.30916769	4.774128553	1.910714087	2.863414466	27(26)
Chad	Alzheimer's disease and other dementias	0.240436019	4.272276258	2.056579885	2.215696374	22(21)
Chile	Alzheimer's disease and other dementias	0.771514716	7.228192334	1.910714087	5.317478247	53(53)
China	Alzheimer's disease and other	0.72162976	30.62826427	1.910714087	28.71755019	203(203)

	dementias					
Colombia	Alzheimer's disease and other dementias	0.655442913	6.181456233	1.912590367	4.268865866	42(42)
Comoros	Alzheimer's disease and other dementias	0.475978688	8.63119406	1.910714087	6.720479973	68(68)
Congo	Alzheimer's disease and other dementias	0.583075236	9.000497674	1.912558448	7.087939226	71(71)
Cook Islands	Alzheimer's disease and other dementias	0.779109955	11.45143353	1.911051652	9.540381881	96(96)
Costa Rica	Alzheimer's disease and other dementias	0.700340477	9.898786458	1.912908894	7.985877564	81(81)
Croatia	Alzheimer's disease and other dementias	0.798341027	22.3754105	1.911318042	20.46409245	186(186)
Cuba	Alzheimer's disease and other dementias	0.668729864	12.76749792	1.912067003	10.85543091	115(115)
Cyprus	Alzheimer's disease and other dementias	0.835630545	19.94355951	1.912500975	18.03105854	169(169)
Czechia	Alzheimer's disease and other dementias	0.828450433	17.56972852	1.912172824	15.65755569	155(155)
Côte d'Ivoire	Alzheimer's disease and other dementias	0.425941883	4.076642888	1.912650716	2.163992172	21(20)
Democratic People's Republic of Korea	Alzheimer's disease and other dementias	0.569854634	12.16254467	1.910883949	10.25166072	105(105)
Democratic Republic of the Congo	Alzheimer's disease and other dementias	0.383179849	3.485044267	1.910714087	1.57433018	16(15)
Denmark	Alzheimer's disease and other	0.896424204	26.38616012	1.910727594	24.47543253	197(197)

	dementias					
Djibouti	Alzheimer's disease and other dementias	0.487958371	13.36297912	1.910770348	11.45220878	119(119)
Dominica	Alzheimer's disease and other dementias	0.746967185	5.369860219	1.910786868	3.459073351	29(28)
Dominican Republic	Alzheimer's disease and other dementias	0.619388201	16.84107618	1.912052938	14.92902324	147(147)
Ecuador	Alzheimer's disease and other dementias	0.661017053	4.992243639	1.912885553	3.079358086	28(27)
Egypt	Alzheimer's disease and other dementias	0.606787094	27.07101277	1.910727594	25.16028518	199(199)
El Salvador	Alzheimer's disease and other dementias	0.563775188	5.687880769	1.910883409	3.776997359	33(32)
Equatorial Guinea	Alzheimer's disease and other dementias	0.657857456	5.762521988	1.910812806	3.851709181	35(34)
Eritrea	Alzheimer's disease and other dementias	0.403863943	2.655828033	1.910714087	0.745113946	10(8)
Estonia	Alzheimer's disease and other dementias	0.844917787	13.52052715	1.91265907	11.60786808	120(120)
Eswatini	Alzheimer's disease and other dementias	0.585459713	7.321831871	1.911696742	5.410135129	55(55)
Ethiopia	Alzheimer's disease and other dementias	0.358823295	3.09466096	1.910941696	1.183719265	12(11)
Fiji	Alzheimer's disease and other dementias	0.675051631	9.117687623	1.91162002	7.206067604	73(73)
Finland	Alzheimer's disease and other	0.859831368	12.50122426	1.912668004	10.58855625	110(110)

	dementias					
France	Alzheimer's disease and other dementias	0.838364875	13.31683014	1.910735185	11.40609496	118(118)
Gabon	Alzheimer's disease and other dementias	0.634691393	5.491126097	1.912038761	3.579087335	30(29)
Gambia	Alzheimer's disease and other dementias	0.40971416	3.959309931	1.910714087	2.048595845	19(18)
Georgia	Alzheimer's disease and other dementias	0.732473604	16.4716862	1.910728152	14.56095804	143(143)
Germany	Alzheimer's disease and other dementias	0.902957091	19.7596457	1.91088287	17.84876283	167(167)
Ghana	Alzheimer's disease and other dementias	0.56493039	4.362729504	1.910714087	2.452015417	23(22)
Greece	Alzheimer's disease and other dementias	0.791854408	24.24536593	1.911127072	22.33423886	190(190)
Greenland	Alzheimer's disease and other dementias	0.826210336	26.92848436	1.910931819	25.01755255	198(198)
Grenada	Alzheimer's disease and other dementias	0.668993028	4.626953906	1.910776543	2.716177363	26(25)
Guam	Alzheimer's disease and other dementias	0.803982203	9.703801087	1.911232174	7.792568913	80(80)
Guatemala	Alzheimer's disease and other dementias	0.539972424	6.992658955	1.912072986	5.080585969	48(48)
Guinea	Alzheimer's disease and other dementias	0.336401293	4.538923981	1.91088287	2.628041112	25(24)
Guinea-Bissau	Alzheimer's disease and other	0.353109621	2.465782836	1.9107982	0.554984636	8(6)

	dementias					
Guyana	Alzheimer's disease and other dementias	0.650812335	5.748328552	1.910798478	3.837530074	34(33)
Haiti	Alzheimer's disease and other dementias	0.448278285	3.938640891	1.91093243	2.027708461	18(17)
Honduras	Alzheimer's disease and other dementias	0.513037248	12.13780964	1.911164174	10.22664547	103(103)
Hungary	Alzheimer's disease and other dementias	0.790754768	12.61551119	1.91162152	10.70388967	112(112)
Iceland	Alzheimer's disease and other dementias	0.87636168	19.07835507	1.911195184	17.16715989	165(165)
India	Alzheimer's disease and other dementias	0.575401649	10.25129543	1.910817859	8.340477567	83(83)
Indonesia	Alzheimer's disease and other dementias	0.656868336	18.42467177	1.910772187	16.51389958	162(162)
Iran (Islamic Republic of)	Alzheimer's disease and other dementias	0.697207398	14.22275734	1.910714087	12.31204325	126(126)
Iraq	Alzheimer's disease and other dementias	0.662626231	25.36432002	1.912126266	23.45219376	192(192)
Ireland	Alzheimer's disease and other dementias	0.87375385	19.47875545	1.910714087	17.56804136	166(166)
Israel	Alzheimer's disease and other dementias	0.809011652	14.57592637	1.912485361	12.66344101	129(129)
Italy	Alzheimer's disease and other dementias	0.805773534	17.40364962	1.912010742	15.49163888	152(152)
Jamaica	Alzheimer's disease and other	0.683263064	8.865071963	1.912527639	6.952544323	69(69)

	dementias					
Japan	Alzheimer's disease and other dementias	0.871241813	15.37446267	1.910714087	13.46374859	137(137)
Jordan	Alzheimer's disease and other dementias	0.725307227	28.23809656	1.912482215	26.32561435	201(201)
Kazakhstan	Alzheimer's disease and other dementias	0.725144495	7.78921277	1.910798478	5.878414291	58(58)
Kenya	Alzheimer's disease and other dementias	0.523768077	6.056835091	1.912638474	4.144196618	39(39)
Kiribati	Alzheimer's disease and other dementias	0.527186583	28.20731272	1.912010742	26.29530198	200(200)
Kuwait	Alzheimer's disease and other dementias	0.846651055	22.07718021	1.912010742	20.16516946	184(184)
Kyrgyzstan	Alzheimer's disease and other dementias	0.603979328	18.69913106	1.910718589	16.78841248	163(163)
Lao People's Democratic Republic	Alzheimer's disease and other dementias	0.489136091	20.05369537	1.910714087	18.14298128	171(171)
Latvia	Alzheimer's disease and other dementias	0.830663516	10.44594632	1.910714087	8.535232233	88(88)
Lebanon	Alzheimer's disease and other dementias	0.744746351	35.63073987	1.910714087	33.72002578	204(204)
Lesotho	Alzheimer's disease and other dementias	0.510393066	16.70130547	1.910714087	14.79059138	145(145)
Liberia	Alzheimer's disease and other dementias	0.352442452	2.616912642	1.912010742	0.704901899	9(7)
Libya	Alzheimer's disease and other	0.725771399	17.71185349	1.911182183	15.80067131	156(156)

	dementias					
Lithuania	Alzheimer's disease and other dementias	0.856484049	10.95327651	1.910736591	9.042539917	93(93)
Luxembourg	Alzheimer's disease and other dementias	0.884428955	13.0091682	1.910945133	11.09822306	116(116)
Madagascar	Alzheimer's disease and other dementias	0.400246943	3.619685206	1.910714087	1.708971119	17(16)
Malawi	Alzheimer's disease and other dementias	0.384553634	10.30034111	1.910936897	8.389404217	85(85)
Malaysia	Alzheimer's disease and other dementias	0.742523828	15.28656798	1.910826609	13.37574137	135(135)
Maldives	Alzheimer's disease and other dementias	0.650886627	21.71308097	1.912010742	19.80107023	182(182)
Mali	Alzheimer's disease and other dementias	0.268579941	7.044700635	1.910718589	5.133982046	51(51)
Malta	Alzheimer's disease and other dementias	0.801585034	13.13847813	1.910967584	11.22751054	117(117)
Marshall Islands	Alzheimer's disease and other dementias	0.574091128	10.69636142	1.910714087	8.785647338	90(90)
Mauritania	Alzheimer's disease and other dementias	0.4989451	3.064775983	1.910816233	1.153959749	11(10)
Mauritius	Alzheimer's disease and other dementias	0.718260446	14.28953661	1.911663877	12.37787273	128(128)
Mexico	Alzheimer's disease and other dementias	0.664575304	6.076750594	1.911051652	4.165698942	40(40)
Micronesia (Federated States of)	Alzheimer's disease and other	0.587534967	15.8505859	1.910732096	13.93985381	141(141)

	dementias					
Monaco	Alzheimer's disease and other dementias	0.908262831	18.40114396	1.911389218	16.48975474	161(161)
Mongolia	Alzheimer's disease and other dementias	0.617621565	14.72431159	1.910714087	12.8135975	130(130)
Montenegro	Alzheimer's disease and other dementias	0.795800584	20.30590055	1.91083003	18.39507052	175(175)
Morocco	Alzheimer's disease and other dementias	0.562698301	7.921637274	1.912132304	6.00950497	60(60)
Mozambique	Alzheimer's disease and other dementias	0.326462614	7.039166387	1.911393536	5.127772851	50(50)
Myanmar	Alzheimer's disease and other dementias	0.53390084	14.93225998	1.912493217	13.01976676	131(131)
Namibia	Alzheimer's disease and other dementias	0.617564872	15.67996693	1.910738099	13.76922883	139(139)
Nauru	Alzheimer's disease and other dementias	0.625177834	14.05904898	1.912010742	12.14703824	124(124)
Nepal	Alzheimer's disease and other dementias	0.433174635	18.85631318	1.910714087	16.94559909	164(164)
Netherlands	Alzheimer's disease and other dementias	0.888464256	24.04025661	1.910765163	22.12949145	189(189)
New Zealand	Alzheimer's disease and other dementias	0.849442499	17.55416431	1.912477007	15.6416873	154(154)
Nicaragua	Alzheimer's disease and other dementias	0.523958472	8.334294137	1.910714087	6.42358005	63(63)
Niger	Alzheimer's disease and other	0.168072774	2.919040201	2.471424741	0.44761546	6(9)

	dementias					
Nigeria	Alzheimer's disease and other dementias	0.503390833	2.043875908	1.9117703	0.132105608	2(1)
Niue	Alzheimer's disease and other dementias	0.72622205	10.53270106	1.912137329	8.620563729	89(89)
North Macedonia	Alzheimer's disease and other dementias	0.750629703	20.9638527	1.910714087	19.05313861	179(179)
Northern Mariana Islands	Alzheimer's disease and other dementias	0.771535213	11.63442531	1.912010742	9.722414565	99(99)
Norway	Alzheimer's disease and other dementias	0.91613281	13.86888793	1.912462588	11.95642535	122(122)
Oman	Alzheimer's disease and other dementias	0.773391602	8.609286086	1.91088287	6.698403217	67(67)
Pakistan	Alzheimer's disease and other dementias	0.504028689	11.15341713	1.910756283	9.242660852	95(95)
Palau	Alzheimer's disease and other dementias	0.754046931	10.7564811	1.912029496	8.844451604	92(92)
Palestine	Alzheimer's disease and other dementias	0.631011665	20.24312136	1.910714087	18.33240728	174(174)
Panama	Alzheimer's disease and other dementias	0.708864828	8.061985489	1.910714087	6.151271402	61(61)
Papua New Guinea	Alzheimer's disease and other dementias	0.417797443	9.477761593	1.911038251	7.566723342	76(76)
Paraguay	Alzheimer's disease and other dementias	0.635718099	25.43485719	1.91093913	23.52391806	193(193)
Peru	Alzheimer's disease and other	0.662054037	5.884876891	1.912444962	3.972431929	37(37)

	dementias					
Philippines	Alzheimer's disease and other dementias	0.651219329	16.8332299	1.910865076	14.92236482	146(146)
Poland	Alzheimer's disease and other dementias	0.812042809	14.93233198	1.911389218	13.02094276	132(132)
Portugal	Alzheimer's disease and other dementias	0.744151851	7.916685746	1.912011868	6.004673878	59(59)
Puerto Rico	Alzheimer's disease and other dementias	0.825525847	9.119644903	1.912128486	7.207516417	74(74)
Qatar	Alzheimer's disease and other dementias	0.846860584	11.60420764	1.910714087	9.693493554	98(98)
Republic of Korea	Alzheimer's disease and other dementias	0.886675267	20.97918144	1.910732096	19.06844934	180(180)
Republic of Moldova	Alzheimer's disease and other dementias	0.732214875	11.86464052	1.912469975	9.95217055	100(100)
Romania	Alzheimer's disease and other dementias	0.768453864	10.26886131	1.91206477	8.356796545	84(84)
Russian Federation	Alzheimer's disease and other dementias	0.808536005	12.3051856	1.910826609	10.39435899	108(108)
Rwanda	Alzheimer's disease and other dementias	0.435588706	26.26482779	1.91091326	24.35391453	196(196)
Saint Kitts and Nevis	Alzheimer's disease and other dementias	0.754987055	4.516481434	1.912238787	2.604242647	24(23)
Saint Lucia	Alzheimer's disease and other dementias	0.672509735	6.001929161	1.910714087	4.091215074	38(38)
Saint Vincent and the Grenadines	Alzheimer's disease and other	0.637195963	6.128976373	1.910714087	4.218262286	41(41)

	dementias					
Samoa	Alzheimer's disease and other dementias	0.593392769	17.82218377	1.910854739	15.91132903	157(157)
San Marino	Alzheimer's disease and other dementias	0.888005474	15.88037704	1.910714087	13.96966295	142(142)
Sao Tome and Principe	Alzheimer's disease and other dementias	0.505413747	2.172883643	1.910919103	0.26196454	3(2)
Saudi Arabia	Alzheimer's disease and other dementias	0.815143493	9.635288014	1.912017496	7.723270518	78(78)
Senegal	Alzheimer's disease and other dementias	0.408054193	3.135134184	1.912804242	1.222329942	13(12)
Serbia	Alzheimer's disease and other dementias	0.792416294	15.77951312	1.910714087	13.86879903	140(140)
Seychelles	Alzheimer's disease and other dementias	0.730150775	15.13456861	1.910714087	13.22385452	134(134)
Sierra Leone	Alzheimer's disease and other dementias	0.358665881	3.305441403	1.910967261	1.394474142	15(14)
Singapore	Alzheimer's disease and other dementias	0.856097766	6.604923421	1.910755724	4.694167697	44(44)
Slovakia	Alzheimer's disease and other dementias	0.81061053	12.4929797	1.910714087	10.58226562	109(109)
Slovenia	Alzheimer's disease and other dementias	0.842430731	13.72662001	1.91093913	11.81568088	121(121)
Solomon Islands	Alzheimer's disease and other dementias	0.429360316	17.3331233	1.910756373	15.42236693	150(150)
Somalia	Alzheimer's disease and other	0.077688109	5.795879743	5.795879743	0	1(35)

	dementias					
South Africa	Alzheimer's disease and other dementias	0.679626598	7.404147561	1.912842998	5.491304563	56(56)
South Sudan	Alzheimer's disease and other dementias	0.278371125	6.958354364	1.912010742	5.046343621	47(47)
Spain	Alzheimer's disease and other dementias	0.769283698	12.61682924	1.911254191	10.70557504	113(113)
Sri Lanka	Alzheimer's disease and other dementias	0.701534935	7.277756089	1.912494533	5.365261556	54(54)
Sudan	Alzheimer's disease and other dementias	0.541949735	15.42632698	1.911272229	13.51505475	138(138)
Suriname	Alzheimer's disease and other dementias	0.633665739	8.899756707	1.91169998	6.988056727	70(70)
Sweden	Alzheimer's disease and other dementias	0.886880299	22.23878368	1.910826609	20.32795708	185(185)
Switzerland	Alzheimer's disease and other dementias	0.933059111	20.03495415	1.910714087	18.12424007	170(170)
Syrian Arab Republic	Alzheimer's disease and other dementias	0.623004075	20.18849034	1.910827328	18.27766301	173(173)
Taiwan (Province of China)	Alzheimer's disease and other dementias	0.874747053	12.10713802	1.912034755	10.19510326	102(102)
Tajikistan	Alzheimer's disease and other dementias	0.541511187	9.572115169	1.912072986	7.660042184	77(77)
Thailand	Alzheimer's disease and other dementias	0.682547933	14.05980994	1.911397411	12.14841253	125(125)
Timor-Leste	Alzheimer's disease and other	0.444667619	16.68268558	1.912531955	14.77015362	144(144)

	dementias					
Togo	Alzheimer's disease and other dementias	0.408533695	5.812224209	1.912019747	3.900204462	36(36)
Tokelau	Alzheimer's disease and other dementias	0.686425621	12.68174036	1.910714087	10.77102627	114(114)
Tonga	Alzheimer's disease and other dementias	0.626349936	20.17569885	1.910764097	18.26493476	172(172)
Trinidad and Tobago	Alzheimer's disease and other dementias	0.768763254	7.551660279	1.910963391	5.640696888	57(57)
Tunisia	Alzheimer's disease and other dementias	0.682432216	25.00841462	1.910723091	23.09769153	191(191)
Turkmenistan	Alzheimer's disease and other dementias	0.682160776	8.407749833	1.911051652	6.49669818	64(64)
Tuvalu	Alzheimer's disease and other dementias	0.576620529	14.26297622	1.91094057	12.35203565	127(127)
Türkiye	Alzheimer's disease and other dementias	0.712692673	17.33875898	1.911366397	15.42739258	151(151)
Uganda	Alzheimer's disease and other dementias	0.423261181	6.533749396	1.910817914	4.622931482	43(43)
Ukraine	Alzheimer's disease and other dementias	0.760773913	10.38639478	1.911052027	8.475342752	86(86)
United Arab Emirates	Alzheimer's disease and other dementias	0.849317734	20.88562284	1.910714087	18.97490875	178(178)
United Kingdom	Alzheimer's disease and other dementias	0.859000182	18.18345228	1.912334906	16.27111737	159(159)
United Republic of Tanzania	Alzheimer's disease and other	0.446568273	10.42769869	1.912302533	8.51539616	87(87)

	dementias					
United States of America	Alzheimer's disease and other dementias	0.862448354	23.74857678	1.912014119	21.83656266	188(188)
United States Virgin Islands	Alzheimer's disease and other dementias	0.821830853	7.124367826	1.912113506	5.21225432	52(52)
Uruguay	Alzheimer's disease and other dementias	0.719283445	12.14404572	1.912442961	10.23160276	104(104)
Uzbekistan	Alzheimer's disease and other dementias	0.662621694	7.029880638	1.910714087	5.119166551	49(49)
Vanuatu	Alzheimer's disease and other dementias	0.473100706	8.154048161	1.910723901	6.24332426	62(62)
Venezuela (Bolivarian Republic of)	Alzheimer's disease and other dementias	0.596513059	9.089747972	1.912179525	7.177568447	72(72)
Viet Nam	Alzheimer's disease and other dementias	0.627933721	17.01369838	1.910815246	15.10288314	148(148)
Yemen	Alzheimer's disease and other dementias	0.450376375	21.80290565	1.910798478	19.89210717	183(183)
Zambia	Alzheimer's disease and other dementias	0.505948954	10.0422816	1.912804761	8.129476837	82(82)
Zimbabwe	Alzheimer's disease and other dementias	0.473819486	15.08552874	1.912051263	13.17347747	133(133)
Afghanistan	Multiple sclerosis	0.337199998	0.831190632	0.021607025	0.809583607	123(123)
Albania	Multiple sclerosis	0.706849791	9.71286705	0.02155036	9.691316689	203(203)
Algeria	Multiple sclerosis	0.659500924	1.557774779	0.021535766	1.536239013	143(143)
American Samoa	Multiple sclerosis	0.723727533	0.057166097	0.021614419	0.035551678	43(43)
Andorra	Multiple sclerosis	0.869444113	5.99522295	0.021586765	5.973636185	186(186)

Angola	Multiple sclerosis	0.453721949	0.120165207	0.021557431	0.098607776	75(75)
Antigua and Barbuda	Multiple sclerosis	0.749886887	0.570485228	0.021526565	0.548958663	114(114)
Argentina	Multiple sclerosis	0.723122973	1.610569371	0.021599151	1.58897022	144(144)
Armenia	Multiple sclerosis	0.701833194	1.619429055	0.021535605	1.597893451	145(145)
Australia	Multiple sclerosis	0.844252814	3.107896583	0.021531196	3.086365388	166(166)
Austria	Multiple sclerosis	0.853837004	8.260571922	0.021551689	8.239020233	199(199)
Azerbaijan	Multiple sclerosis	0.694851274	0.990914228	0.021541235	0.969372993	129(129)
Bahamas	Multiple sclerosis	0.805020668	1.06896771	0.021549114	1.047418596	132(132)
Bahrain	Multiple sclerosis	0.753043204	1.096286053	0.021587568	1.074698485	133(133)
Bangladesh	Multiple sclerosis	0.492420885	0.184469341	0.021536245	0.162933096	85(85)
Barbados	Multiple sclerosis	0.746748764	0.664346611	0.021534195	0.642812415	118(118)
Belarus	Multiple sclerosis	0.784484711	2.493762722	0.021614126	2.472148597	158(158)
Belgium	Multiple sclerosis	0.853654016	7.744083014	0.021534532	7.722548482	195(195)
Belize	Multiple sclerosis	0.610229002	0.284726369	0.021571163	0.263155205	97(97)
Benin	Multiple sclerosis	0.373486574	0.03219231	0.021541436	0.010650874	9(8)
Bermuda	Multiple sclerosis	0.821365422	0.92535372	0.021575142	0.903778578	127(127)
Bhutan	Multiple sclerosis	0.473062378	0.130971961	0.021563382	0.109408579	76(76)
Bolivia (Plurinational State of)	Multiple sclerosis	0.599010799	0.255018247	0.02153458	0.233483667	93(93)
Bosnia and Herzegovina	Multiple sclerosis	0.723077893	3.763164746	0.021530518	3.741634228	173(173)
Botswana	Multiple sclerosis	0.642721629	0.151210208	0.021536348	0.12967386	80(80)
Brazil	Multiple sclerosis	0.653043887	0.896377425	0.021539004	0.874838421	125(125)
Brunei Darussalam	Multiple sclerosis	0.810234367	0.202043384	0.021582766	0.180460618	89(89)
Bulgaria	Multiple sclerosis	0.768150939	6.59238549	0.021576569	6.570808921	188(188)
Burkina Faso	Multiple sclerosis	0.285118402	0.038228849	0.021605806	0.016623043	16(15)
Burundi	Multiple sclerosis	0.289374365	0.03049722	0.021609666	0.008887554	5(4)
Cabo Verde	Multiple sclerosis	0.533534539	0.053219951	0.021525291	0.03169466	35(35)

Cambodia	Multiple sclerosis	0.473621491	0.071116786	0.021595232	0.049521554	59(59)
Cameroon	Multiple sclerosis	0.479691223	0.047763821	0.021534195	0.026229626	27(27)
Canada	Multiple sclerosis	0.87317068	8.004892885	0.0215395	7.983353385	197(197)
Central African Republic	Multiple sclerosis	0.30916769	0.047115385	0.021537427	0.025577958	26(26)
Chad	Multiple sclerosis	0.240436019	0.048702986	0.021589839	0.027113147	28(28)
Chile	Multiple sclerosis	0.771514716	0.850661011	0.02152728	0.829133731	124(124)
China	Multiple sclerosis	0.72162976	0.092916134	0.021530027	0.071386107	70(70)
Colombia	Multiple sclerosis	0.655442913	0.281895899	0.021576972	0.260318927	96(96)
Comoros	Multiple sclerosis	0.475978688	0.062193035	0.021615346	0.04057769	51(51)
Congo	Multiple sclerosis	0.583075236	0.070309943	0.021526718	0.048783225	57(57)
Cook Islands	Multiple sclerosis	0.779109955	0.063067382	0.021527976	0.041539407	52(52)
Costa Rica	Multiple sclerosis	0.700340477	0.598143902	0.021525102	0.5766188	115(115)
Croatia	Multiple sclerosis	0.798341027	4.068784095	0.021526665	4.047257431	175(175)
Cuba	Multiple sclerosis	0.668729864	1.790440313	0.02154243	1.768897883	148(148)
Cyprus	Multiple sclerosis	0.835630545	4.598137024	0.021591481	4.576545543	179(179)
Czechia	Multiple sclerosis	0.828450433	4.571745306	0.021611984	4.550133322	178(178)
C 么 te d'Ivoire	Multiple sclerosis	0.425941883	0.05936191	0.02158845	0.03777346	46(46)
Democratic People's Republic of Korea	Multiple sclerosis	0.569854634	0.096692326	0.021525902	0.075166424	73(73)
Democratic Republic of the Congo	Multiple sclerosis	0.383179849	0.031155106	0.021538546	0.00961656	6(5)
Denmark	Multiple sclerosis	0.896424204	11.60788826	0.021594468	11.58629379	204(204)
Djibouti	Multiple sclerosis	0.487958371	0.102589279	0.021534432	0.081054846	74(74)
Dominica	Multiple sclerosis	0.746967185	0.293673119	0.021526449	0.27214667	99(99)
Dominican Republic	Multiple sclerosis	0.619388201	0.363875279	0.021541424	0.342333856	103(103)
Ecuador	Multiple sclerosis	0.661017053	0.245989458	0.021588419	0.224401039	92(92)
Egypt	Multiple sclerosis	0.606787094	0.751715475	0.021526917	0.730188557	120(120)

El Salvador	Multiple sclerosis	0.563775188	0.289248273	0.021538742	0.267709531	98(98)
Equatorial Guinea	Multiple sclerosis	0.657857456	0.060565523	0.021607898	0.038957625	49(49)
Eritrea	Multiple sclerosis	0.403863943	0.055746	0.021537605	0.034208394	40(40)
Estonia	Multiple sclerosis	0.844917787	2.677863456	0.021543619	2.656319837	162(162)
Eswatini	Multiple sclerosis	0.585459713	0.055699736	0.021548905	0.034150831	39(39)
Ethiopia	Multiple sclerosis	0.358823295	0.023913443	0.021538815	0.002374628	3(2)
Fiji	Multiple sclerosis	0.675051631	0.039342923	0.021600825	0.017742098	18(17)
Finland	Multiple sclerosis	0.859831368	5.182119111	0.021546287	5.160572824	182(182)
France	Multiple sclerosis	0.838364875	5.285367508	0.021527122	5.263840385	183(183)
Gabon	Multiple sclerosis	0.634691393	0.071662465	0.021599183	0.050063282	61(61)
Gambia	Multiple sclerosis	0.40971416	0.064418801	0.021542735	0.042876066	54(54)
Georgia	Multiple sclerosis	0.732473604	1.164431563	0.021530198	1.142901365	136(136)
Germany	Multiple sclerosis	0.902957091	6.69127984	0.021542077	6.669737764	191(191)
Ghana	Multiple sclerosis	0.56493039	0.07559713	0.021535397	0.054061733	62(62)
Greece	Multiple sclerosis	0.791854408	5.828827424	0.021536469	5.807290955	185(185)
Greenland	Multiple sclerosis	0.826210336	3.708489617	0.021532847	3.68695677	172(172)
Grenada	Multiple sclerosis	0.668993028	0.619481099	0.021525502	0.597955597	117(117)
Guam	Multiple sclerosis	0.803982203	0.051142143	0.021528662	0.029613482	31(31)
Guatemala	Multiple sclerosis	0.539972424	0.198945988	0.021547075	0.177398913	87(87)
Guinea	Multiple sclerosis	0.336401293	0.053018406	0.02159167	0.031426736	34(34)
Guinea-Bissau	Multiple sclerosis	0.353109621	0.044866135	0.021529648	0.023336487	23(23)
Guyana	Multiple sclerosis	0.650812335	0.309961516	0.02153625	0.288425266	101(101)
Haiti	Multiple sclerosis	0.448278285	0.166461071	0.021587063	0.144874008	81(81)
Honduras	Multiple sclerosis	0.513037248	0.229709702	0.02157586	0.208133842	90(90)
Hungary	Multiple sclerosis	0.790754768	4.131904631	0.021603141	4.11030149	176(176)
Iceland	Multiple sclerosis	0.87636168	8.361534489	0.02156132	8.339973169	200(200)

India	Multiple sclerosis	0.575401649	0.140311523	0.021535576	0.118775947	79(79)
Indonesia	Multiple sclerosis	0.656868336	0.056748537	0.021538535	0.035210002	41(41)
Iran (Islamic Republic of)	Multiple sclerosis	0.697207398	2.243772901	0.02153231	2.222240591	154(154)
Iraq	Multiple sclerosis	0.662626231	1.329655657	0.021535357	1.3081203	140(140)
Ireland	Multiple sclerosis	0.87375385	6.748802174	0.021526238	6.727275936	193(193)
Israel	Multiple sclerosis	0.809011652	1.967576714	0.021584014	1.9459927	149(149)
Italy	Multiple sclerosis	0.805773534	4.257650512	0.021548378	4.236102134	177(177)
Jamaica	Multiple sclerosis	0.683263064	0.546289448	0.021526904	0.524762545	113(113)
Japan	Multiple sclerosis	0.871241813	0.388782169	0.021550657	0.367231511	104(104)
Jordan	Multiple sclerosis	0.725307227	2.643042811	0.021533635	2.621509176	160(160)
Kazakhstan	Multiple sclerosis	0.725144495	2.263645572	0.021565345	2.242080227	155(155)
Kenya	Multiple sclerosis	0.523768077	0.052840069	0.021531543	0.031308526	33(33)
Kiribati	Multiple sclerosis	0.527186583	0.07135333	0.02161888	0.049734449	60(60)
Kuwait	Multiple sclerosis	0.846651055	1.679076892	0.021567826	1.657509066	147(147)
Kyrgyzstan	Multiple sclerosis	0.603979328	1.123342857	0.021534231	1.101808627	135(135)
Lao People's Democratic Republic	Multiple sclerosis	0.489136091	0.059996438	0.021534873	0.038461565	48(48)
Latvia	Multiple sclerosis	0.830663516	3.350298187	0.021603503	3.328694684	168(168)
Lebanon	Multiple sclerosis	0.744746351	3.660413178	0.021545309	3.638867869	171(171)
Lesotho	Multiple sclerosis	0.510393066	0.199423236	0.021587913	0.177835323	88(88)
Liberia	Multiple sclerosis	0.352442452	0.036122168	0.021535332	0.014586836	13(12)
Libya	Multiple sclerosis	0.725771399	2.277833879	0.021538339	2.25629554	156(156)
Lithuania	Multiple sclerosis	0.856484049	3.466589599	0.021526007	3.445063592	169(169)
Luxembourg	Multiple sclerosis	0.884428955	6.068498295	0.02158006	6.046918235	187(187)
Madagascar	Multiple sclerosis	0.400246943	0.039418159	0.021586999	0.01783116	19(18)
Malawi	Multiple sclerosis	0.384553634	0.084798216	0.02160281	0.063195407	64(64)
Malaysia	Multiple sclerosis	0.742523828	0.050715332	0.021555004	0.029160328	30(30)

Maldives	Multiple sclerosis	0.650886627	0.054075693	0.021553146	0.032522547	36(36)
Mali	Multiple sclerosis	0.268579941	0.056979611	0.021550611	0.035429001	42(42)
Malta	Multiple sclerosis	0.801585034	2.721497558	0.02154313	2.699954428	163(163)
Marshall Islands	Multiple sclerosis	0.574091128	0.037107938	0.021534525	0.015573413	15(14)
Mauritania	Multiple sclerosis	0.4989451	0.092155677	0.021529943	0.070625734	69(69)
Mauritius	Multiple sclerosis	0.718260446	0.260183925	0.021588071	0.238595855	94(94)
Mexico	Multiple sclerosis	0.664575304	0.462015042	0.021587029	0.440428013	106(106)
Micronesia (Federated States of)	Multiple sclerosis	0.587534967	0.051858655	0.02153268	0.030325975	32(32)
Monaco	Multiple sclerosis	0.908262831	3.484551979	0.021533982	3.463017997	170(170)
Mongolia	Multiple sclerosis	0.617621565	1.167437141	0.021573846	1.145863295	137(137)
Montenegro	Multiple sclerosis	0.795800584	8.213360237	0.021588182	8.191772055	198(198)
Morocco	Multiple sclerosis	0.562698301	0.955266681	0.021615124	0.933651557	128(128)
Mozambique	Multiple sclerosis	0.326462614	0.067444716	0.021606366	0.04583835	55(55)
Myanmar	Multiple sclerosis	0.53390084	0.038545746	0.021535857	0.017009889	17(16)
Namibia	Multiple sclerosis	0.617564872	0.088862876	0.021541016	0.067321861	68(68)
Nauru	Multiple sclerosis	0.625177834	0.045262477	0.021535711	0.023726766	24(24)
Nepal	Multiple sclerosis	0.433174635	0.175548441	0.021593546	0.153954895	83(83)
Netherlands	Multiple sclerosis	0.888464256	6.704583981	0.021534195	6.683049786	192(192)
New Zealand	Multiple sclerosis	0.849442499	2.101528436	0.021527126	2.08000131	153(153)
Nicaragua	Multiple sclerosis	0.523958472	0.269717462	0.021538664	0.248178798	95(95)
Niger	Multiple sclerosis	0.168072774	0.029302118	0.023291221	0.006010897	4(3)
Nigeria	Multiple sclerosis	0.503390833	0.03205406	0.021536856	0.010517204	8(7)
Niue	Multiple sclerosis	0.72622205	0.054523951	0.021527375	0.032996576	37(37)
North Macedonia	Multiple sclerosis	0.750629703	5.472605867	0.021534174	5.451071693	184(184)
Northern Mariana Islands	Multiple sclerosis	0.771535213	0.054789474	0.021536051	0.033253424	38(38)
Norway	Multiple sclerosis	0.91613281	7.956157012	0.021527576	7.934629437	196(196)

Oman	Multiple sclerosis	0.773391602	0.992919821	0.02153676	0.971383061	130(130)
Pakistan	Multiple sclerosis	0.504028689	0.190565649	0.021555055	0.169010594	86(86)
Palau	Multiple sclerosis	0.754046931	0.03267921	0.021593278	0.011085932	11(10)
Palestine	Multiple sclerosis	0.631011665	2.594001566	0.02152967	2.572471896	159(159)
Panama	Multiple sclerosis	0.708864828	0.241307065	0.021533487	0.219773578	91(91)
Papua New Guinea	Multiple sclerosis	0.417797443	0.032296313	0.021573237	0.010723076	10(9)
Paraguay	Multiple sclerosis	0.635718099	0.537245362	0.021545579	0.515699783	111(111)
Peru	Multiple sclerosis	0.662054037	0.170008429	0.021550232	0.148458198	82(82)
Philippines	Multiple sclerosis	0.651219329	0.135359585	0.021529006	0.113830579	77(77)
Poland	Multiple sclerosis	0.812042809	4.88814308	0.021585061	4.866558019	180(180)
Portugal	Multiple sclerosis	0.744151851	2.021033623	0.021625154	1.999408469	151(151)
Puerto Rico	Multiple sclerosis	0.825525847	1.119585167	0.021596848	1.097988318	134(134)
Qatar	Multiple sclerosis	0.846860584	1.342805978	0.02156097	1.321245008	141(141)
Republic of Korea	Multiple sclerosis	0.886675267	0.30314908	0.02158973	0.281559351	100(100)
Republic of Moldova	Multiple sclerosis	0.732214875	1.201095649	0.021532977	1.179562672	138(138)
Romania	Multiple sclerosis	0.768453864	2.005542661	0.021531211	1.984011449	150(150)
Russian Federation	Multiple sclerosis	0.808536005	2.862942179	0.0215413	2.841400879	164(164)
Rwanda	Multiple sclerosis	0.435588706	0.09627642	0.021597381	0.074679039	72(72)
Saint Kitts and Nevis	Multiple sclerosis	0.754987055	0.713525355	0.021584787	0.691940568	119(119)
Saint Lucia	Multiple sclerosis	0.672509735	0.481322074	0.021536247	0.459785827	108(108)
Saint Vincent and the Grenadines	Multiple sclerosis	0.637195963	0.177825718	0.021527002	0.156298716	84(84)
Samoa	Multiple sclerosis	0.593392769	0.070576619	0.021587313	0.048989306	58(58)
San Marino	Multiple sclerosis	0.888005474	1.516835392	0.021624157	1.495211235	142(142)
Sao Tome and Principe	Multiple sclerosis	0.505413747	0.033392531	0.021530506	0.011862024	12(11)
Saudi Arabia	Multiple sclerosis	0.815143493	0.790715672	0.021535605	0.769180067	122(122)
Senegal	Multiple sclerosis	0.408054193	0.069119325	0.021547889	0.047571436	56(56)

Serbia	Multiple sclerosis	0.792416294	6.688219153	0.021551393	6.66666776	190(190)
Seychelles	Multiple sclerosis	0.730150775	0.0584126	0.021558387	0.036854213	44(44)
Sierra Leone	Multiple sclerosis	0.358665881	0.049147099	0.021535191	0.027611908	29(29)
Singapore	Multiple sclerosis	0.856097766	0.087651041	0.021543209	0.066107832	66(66)
Slovakia	Multiple sclerosis	0.81061053	2.940003146	0.021539508	2.918463638	165(165)
Slovenia	Multiple sclerosis	0.842430731	4.976440549	0.021539048	4.954901501	181(181)
Solomon Islands	Multiple sclerosis	0.429360316	0.063296594	0.021537809	0.041758785	53(53)
Somalia	Multiple sclerosis	0.077688109	0.039517848	0.037676063	0.001841785	2(19)
South Africa	Multiple sclerosis	0.679626598	0.341807315	0.021545377	0.320261938	102(102)
South Sudan	Multiple sclerosis	0.278371125	0.046992085	0.021591684	0.025400401	25(25)
Spain	Multiple sclerosis	0.769283698	3.840928825	0.021577635	3.81935119	174(174)
Sri Lanka	Multiple sclerosis	0.701534935	0.03189249	0.021585705	0.010306785	7(6)
Sudan	Multiple sclerosis	0.541949735	0.925308846	0.021602318	0.903706528	126(126)
Suriname	Multiple sclerosis	0.633665739	0.603562101	0.021585915	0.581976186	116(116)
Sweden	Multiple sclerosis	0.886880299	9.486523935	0.021531922	9.464992014	202(202)
Switzerland	Multiple sclerosis	0.933059111	6.608730651	0.021589173	6.587141478	189(189)
Syrian Arab Republic	Multiple sclerosis	0.623004075	1.052656479	0.021530424	1.031126055	131(131)
Taiwan (Province of China)	Multiple sclerosis	0.874747053	0.136922959	0.021540258	0.115382701	78(78)
Tajikistan	Multiple sclerosis	0.541511187	0.426170142	0.021537479	0.404632663	105(105)
Thailand	Multiple sclerosis	0.682547933	0.059153345	0.021589921	0.037563424	45(45)
Timor-Leste	Multiple sclerosis	0.444667619	0.043598276	0.021536469	0.022061807	20(20)
Togo	Multiple sclerosis	0.408533695	0.085561871	0.021571573	0.063990298	65(65)
Tokelau	Multiple sclerosis	0.686425621	0.043619329	0.021527943	0.022091386	21(21)
Tonga	Multiple sclerosis	0.626349936	0.059564615	0.021587439	0.037977175	47(47)
Trinidad and Tobago	Multiple sclerosis	0.768763254	0.500590745	0.021536917	0.479053827	109(109)
Tunisia	Multiple sclerosis	0.682432216	2.06806057	0.021588023	2.046472546	152(152)

Turkmenistan	Multiple sclerosis	0.682160776	1.657488687	0.021543323	1.635945364	146(146)
Tuvalu	Multiple sclerosis	0.576620529	0.044194937	0.02154293	0.022652007	22(22)
Türkiye	Multiple sclerosis	0.712692673	2.473453599	0.021525102	2.451928497	157(157)
Uganda	Multiple sclerosis	0.423261181	0.036907164	0.021573448	0.015333716	14(13)
Ukraine	Multiple sclerosis	0.760773913	3.143196767	0.021612735	3.121584032	167(167)
United Arab Emirates	Multiple sclerosis	0.849317734	0.789603844	0.02157657	0.768027274	121(121)
United Kingdom	Multiple sclerosis	0.859000182	8.548259425	0.021560366	8.526699058	201(201)
United Republic of Tanzania	Multiple sclerosis	0.446568273	0.087812897	0.021605972	0.066206926	67(67)
United States of America	Multiple sclerosis	0.862448354	6.789647035	0.021557526	6.768089509	194(194)
United States Virgin Islands	Multiple sclerosis	0.821830853	0.50960521	0.021530944	0.488074266	110(110)
Uruguay	Multiple sclerosis	0.719283445	2.664721879	0.021542421	2.643179459	161(161)
Uzbekistan	Multiple sclerosis	0.662621694	0.46494469	0.021528512	0.443416179	107(107)
Vanuatu	Multiple sclerosis	0.473100706	0.02235999	0.021589052	0.000770938	1(1)
Venezuela (Bolivarian Republic of)	Multiple sclerosis	0.596513059	0.538635302	0.021577265	0.517058037	112(112)
Viet Nam	Multiple sclerosis	0.627933721	0.081357231	0.021525607	0.059831625	63(63)
Yemen	Multiple sclerosis	0.450376375	1.312095683	0.021526283	1.290569401	139(139)
Zambia	Multiple sclerosis	0.505948954	0.060822407	0.021540287	0.039282121	50(50)
Zimbabwe	Multiple sclerosis	0.473819486	0.094388636	0.02154188	0.072846756	71(71)
Afghanistan	Parkinson's disease	0.337199998	-4.720926069	-15.98836251	11.26743644	75(118)
Albania	Parkinson's disease	0.706849791	-15.30232993	-18.44488529	3.14255536	4(2)
Algeria	Parkinson's disease	0.659500924	-7.342092625	-18.21560028	10.87350766	63(52)
American Samoa	Parkinson's disease	0.723727533	-5.935879814	-18.4273828	12.49150304	106(87)

				5		
Andorra	Parkinson's disease	0.869444113	-6.61689232	-18.8932159<br>8	12.27632367	99(69)
Angola	Parkinson's disease	0.453721949	-3.146531488	-16.5239853<br>4	13.37745385	132(159)
Antigua and Barbuda	Parkinson's disease	0.749886887	-4.452061393	-18.9642306<br>2	14.51216923	164(126)
Argentina	Parkinson's disease	0.723122973	-5.317828515	-18.4218055<br>4	13.10397703	124(103)
Armenia	Parkinson's disease	0.701833194	-9.389265828	-18.2418887<br>5	8.852622921	31(17)
Australia	Parkinson's disease	0.844252814	-3.60826834	-18.9833654<br>9	15.37509715	195(149)
Austria	Parkinson's disease	0.853837004	-7.437529957	-18.9769933<br>1	11.53946335	78(49)
Azerbaijan	Parkinson's disease	0.694851274	-7.466380729	-18.4451039<br>6	10.97872323	65(48)
Bahamas	Parkinson's disease	0.805020668	-4.197739201	-18.9746078<br>7	14.77686866	171(132)
Bahrain	Parkinson's disease	0.753043204	-7.921201623	-18.9540292<br>1	11.03282759	70(40)
Bangladesh	Parkinson's disease	0.492420885	-8.59388825	-16.681331	8.087442753	23(29)
Barbados	Parkinson's disease	0.746748764	-2.888927325	-18.7851015	15.89617417	201(165)
Belarus	Parkinson's disease	0.784484711	-8.951954966	-18.8439524<br>1	9.891997443	45(27)
Belgium	Parkinson's disease	0.853654016	-7.268015513	-18.5672799	11.29926448	76(54)

				9		
Belize	Parkinson's disease	0.610229002	-4.723550194	-16.6951227<br>6	11.97157256	94(117)
Benin	Parkinson's disease	0.373486574	-1.203243591	-16.5371129<br>6	15.33386937	193(201)
Bermuda	Parkinson's disease	0.821365422	-3.961705766	-18.9573429<br>3	14.99563716	178(139)
Bhutan	Parkinson's disease	0.473062378	-4.143705147	-16.5717416<br>9	12.42803654	103(134)
Bolivia (Plurinational State of)	Parkinson's disease	0.599010799	-5.01184057	-16.6921646<br>9	11.68032412	86(111)
Bosnia and Herzegovina	Parkinson's disease	0.723077893	-10.6017717	-18.3368921<br>4	7.735120441	20(10)
Botswana	Parkinson's disease	0.642721629	-4.313757396	-16.6839928<br>9	12.3702355	101(128)
Brazil	Parkinson's disease	0.653043887	-5.274902646	-17.2358970<br>4	11.9609944	93(106)
Brunei Darussalam	Parkinson's disease	0.810234367	-5.780975069	-18.9388631<br>6	13.1578881	125(93)
Bulgaria	Parkinson's disease	0.768150939	-7.18041838	-18.8413490<br>4	11.66093066	85(59)
Burkina Faso	Parkinson's disease	0.285118402	-1.316532662	-10.9193385	9.60280584	38(197)
Burundi	Parkinson's disease	0.289374365	-1.743666959	-12.2825611<br>9	10.53889423	56(192)
Cabo Verde	Parkinson's disease	0.533534539	-1.477436685	-16.7114778<br>9	15.2340412	183(193)

Cambodia	Parkinson's disease	0.473621491	-12.09911748	-16.5950982<br>1	4.495980725	6(6)
Cameroon	Parkinson's disease	0.479691223	-1.877725058	-16.6794246<br>2	14.80169956	173(187)
Canada	Parkinson's disease	0.87317068	-7.921237128	-18.9507696<br>3	11.0295325	69(39)
Central African Republic	Parkinson's disease	0.30916769	-2.085214383	-13.0900339<br>8	11.00481959	67(184)
Chad	Parkinson's disease	0.240436019	-2.176640802	-10.8829006<br>9	8.706259887	29(182)
Chile	Parkinson's disease	0.771514716	-3.704272373	-18.9467863<br>3	15.24251396	184(147)
China	Parkinson's disease	0.72162976	-15.68270199	-18.4374753	2.75477331	3(1)
Colombia	Parkinson's disease	0.655442913	-2.400427533	-16.9718937<br>7	14.57146624	165(178)
Comoros	Parkinson's disease	0.475978688	-2.915004839	-16.6694618<br>7	13.75445703	142(163)
Congo	Parkinson's disease	0.583075236	-3.081046036	-16.6785702<br>4	13.59752421	137(161)
Cook Islands	Parkinson's disease	0.779109955	-4.750334162	-18.9768463<br>2	14.22651215	158(114)
Costa Rica	Parkinson's disease	0.700340477	-4.132833928	-18.4447932	14.31195927	159(135)
Croatia	Parkinson's disease	0.798341027	-9.07762713	-18.8997752<br>1	9.822148084	43(24)
Cuba	Parkinson's disease	0.668729864	-6.54426928	-18.4400434<br>9	11.89577421	91(72)

Cyprus	Parkinson's disease	0.835630545	-9.201664837	-18.9790193 7	9.777354537	42(22)
Czechia	Parkinson's disease	0.828450433	-7.18776539	-18.7548786 4	11.56711325	79(57)
C 么 te d'Ivoire	Parkinson's disease	0.425941883	-2.477262572	-16.5272553 5	14.04999278	150(177)
Democratic People's Republic of Korea	Parkinson's disease	0.569854634	-8.950009289	-16.6994795 9	7.7494703	21(28)
Democratic Republic of the Congo	Parkinson's disease	0.383179849	-1.249038047	-16.5191080 6	15.27007001	187(199)
Denmark	Parkinson's disease	0.896424204	-10.55667115	-18.9638079 7	8.407136818	25(11)
Djibouti	Parkinson's disease	0.487958371	-4.495236296	-16.7050222 3	12.20978593	96(122)
Dominica	Parkinson's disease	0.746967185	-4.196576509	-18.9746732 6	14.77809675	172(133)
Dominican Republic	Parkinson's disease	0.619388201	-6.506901571	-16.6926234 4	10.18572187	51(73)
Ecuador	Parkinson's disease	0.661017053	-3.541705202	-18.4430346 7	14.90132947	176(150)
Egypt	Parkinson's disease	0.606787094	-9.572834214	-16.7040130 6	7.131178845	14(16)
El Salvador	Parkinson's disease	0.563775188	-2.748816242	-16.6986348 1	13.94981857	145(169)
Equatorial Guinea	Parkinson's disease	0.657857456	-2.347534168	-18.4415537 6	16.0940196	203(180)

Eritrea	Parkinson's disease	0.403863943	-1.229795298	-16.50669686	15.27690156	189(200)
Estonia	Parkinson's disease	0.844917787	-5.60231033	-18.44430353	12.8419932	115(96)
Eswatini	Parkinson's disease	0.585459713	-2.722245695	-16.68607437	13.96382868	147(170)
Ethiopia	Parkinson's disease	0.358823295	-0.90360608	-16.52272269	15.61911661	199(204)
Fiji	Parkinson's disease	0.675051631	-5.851485123	-18.42158639	12.57010127	108(91)
Finland	Parkinson's disease	0.859831368	-5.622471636	-18.78109358	13.15862195	126(94)
France	Parkinson's disease	0.838364875	-6.641407087	-18.91382765	12.27242056	98(68)
Gabon	Parkinson's disease	0.634691393	-2.573672813	-16.69723619	14.12356338	155(171)
Gambia	Parkinson's disease	0.40971416	-2.355100277	-16.51960946	14.16450918	156(179)
Georgia	Parkinson's disease	0.732473604	-8.388289078	-18.45272991	10.06444083	49(32)
Germany	Parkinson's disease	0.902957091	-7.785849097	-18.36243167	10.57658257	58(45)
Ghana	Parkinson's disease	0.56493039	-2.00846015	-16.7037193	14.69525915	168(186)
Greece	Parkinson's disease	0.791854408	-11.98680549	-18.38857352	6.401768033	9(7)
Greenland	Parkinson's disease	0.826210336	-13.82024439	-18.7092347	4.888990357	7(3)

				4		
Grenada	Parkinson's disease	0.668993028	-3.406387776	-18.44425117	15.03786339	179(154)
Guam	Parkinson's disease	0.803982203	-3.30397266	-18.61526699	15.31129433	190(156)
Guatemala	Parkinson's disease	0.539972424	-2.490056714	-16.70243544	14.21237873	157(176)
Guinea	Parkinson's disease	0.336401293	-2.49821578	-15.49299153	12.99477575	118(175)
Guinea-Bissau	Parkinson's disease	0.353109621	-1.77414703	-15.89283355	14.11868652	154(190)
Guyana	Parkinson's disease	0.650812335	-3.771184045	-16.98046593	13.20928188	129(143)
Haiti	Parkinson's disease	0.448278285	-2.907643219	-16.53478564	13.62714242	138(164)
Honduras	Parkinson's disease	0.513037248	-9.382213864	-16.67667669	7.294462824	16(18)
Hungary	Parkinson's disease	0.790754768	-5.505987187	-18.96788245	13.46189527	134(101)
Iceland	Parkinson's disease	0.87636168	-7.943992035	-18.55416972	10.61017768	59(38)
India	Parkinson's disease	0.575401649	-4.827774253	-16.7024932	11.87471895	89(113)
Indonesia	Parkinson's disease	0.656868336	-9.143026242	-18.44372032	9.300694079	35(23)
Iran (Islamic Republic of)	Parkinson's disease	0.697207398	-4.379965239	-18.44059296	14.06062772	151(127)

Iraq	Parkinson's disease	0.662626231	-13.32730328	-18.44053708	5.113233799	8(4)
Ireland	Parkinson's disease	0.87375385	-6.502246847	-18.74795506	12.24570822	97(74)
Israel	Parkinson's disease	0.809011652	-6.193867076	-18.80058386	12.60671678	109(81)
Italy	Parkinson's disease	0.805773534	-6.182448828	-18.93121211	12.74876328	113(82)
Jamaica	Parkinson's disease	0.683263064	-4.085086568	-18.43291249	14.34782592	161(136)
Japan	Parkinson's disease	0.871241813	-4.532510391	-18.95709572	14.42458533	162(121)
Jordan	Parkinson's disease	0.725307227	-8.067249337	-18.44195063	10.37470129	55(36)
Kazakhstan	Parkinson's disease	0.725144495	-4.468510327	-18.19840568	13.72989535	141(125)
Kenya	Parkinson's disease	0.523768077	-2.088125591	-16.68016343	14.59203784	166(183)
Kiribati	Parkinson's disease	0.527186583	-13.20841268	-16.68671975	3.478307071	5(5)
Kuwait	Parkinson's disease	0.846651055	-5.786464785	-18.79230612	13.00584133	120(92)
Kyrgyzstan	Parkinson's disease	0.603979328	-6.850867637	-16.70529572	9.854428084	44(64)
Lao People's Democratic Republic	Parkinson's disease	0.489136091	-9.990661846	-16.67427301	6.683611169	11(13)

Latvia	Parkinson's disease	0.830663516	-5.282790519	-18.81495134	13.53216082	135(105)
Lebanon	Parkinson's disease	0.744746351	-9.220872311	-18.96018621	9.739313899	41(21)
Lesotho	Parkinson's disease	0.510393066	-6.141488912	-16.70033664	10.55884773	57(84)
Liberia	Parkinson's disease	0.352442452	-1.417824689	-16.53635858	15.11853389	181(194)
Libya	Parkinson's disease	0.725771399	-7.423232462	-18.44594257	11.02271011	68(50)
Lithuania	Parkinson's disease	0.856484049	-5.242698461	-18.90948154	13.66678308	139(107)
Luxembourg	Parkinson's disease	0.884428955	-7.910022831	-18.55967429	10.64965146	61(42)
Madagascar	Parkinson's disease	0.400246943	-1.201437907	-16.51709006	15.31565216	191(202)
Malawi	Parkinson's disease	0.384553634	-4.471462291	-16.53694203	12.06547974	95(123)
Malaysia	Parkinson's disease	0.742523828	-6.2176429	-18.90998515	12.69234225	111(80)
Maldives	Parkinson's disease	0.650886627	-7.899928342	-17.02434435	9.124416006	33(43)
Mali	Parkinson's disease	0.268579941	-3.297477678	-10.85769302	7.560215342	18(157)
Malta	Parkinson's disease	0.801585034	-5.605658974	-18.98309386	13.37743488	131(95)

Marshall Islands	Parkinson's disease	0.574091128	-6.574925726	-16.6868436	10.11191787	50(70)
Mauritania	Parkinson's disease	0.4989451	-1.399079958	-16.67360429	15.27452433	188(195)
Mauritius	Parkinson's disease	0.718260446	-6.461813735	-18.35152205	11.88970832	90(76)
Mexico	Parkinson's disease	0.664575304	-3.180118303	-18.43281674	15.25269843	186(158)
Micronesia (Federated States of)	Parkinson's disease	0.587534967	-9.029118554	-16.71173016	7.682611603	19(26)
Monaco	Parkinson's disease	0.908262831	-9.308200836	-18.95968185	9.651481019	39(19)
Mongolia	Parkinson's disease	0.617621565	-6.062912668	-16.69978574	10.63687308	60(85)
Montenegro	Parkinson's disease	0.795800584	-11.46377861	-18.95030742	7.48652881	17(8)
Morocco	Parkinson's disease	0.562698301	-3.863836431	-16.69911487	12.83527844	114(141)
Mozambique	Parkinson's disease	0.326462614	-2.79409862	-13.89300598	11.09890736	72(167)
Myanmar	Parkinson's disease	0.53390084	-6.485027311	-16.6885827	10.20355539	52(75)
Namibia	Parkinson's disease	0.617564872	-5.058326334	-16.69928439	11.64095806	80(109)
Nauru	Parkinson's disease	0.625177834	-9.789572725	-16.70258964	6.913016912	12(15)
Nepal	Parkinson's disease	0.433174635	-8.514795968	-16.53121431	8.016418342	22(30)

Netherlands	Parkinson's disease	0.888464256	-9.073284623	-18.96742973	9.894145111	46(25)
New Zealand	Parkinson's disease	0.849442499	-5.983859051	-18.98336549	12.99950644	119(86)
Nicaragua	Parkinson's disease	0.523958472	-3.365219954	-16.70765573	13.34243577	130(155)
Niger	Parkinson's disease	0.168072774	-1.259576967	-2.948697907	1.68912094	2(198)
Nigeria	Parkinson's disease	0.503390833	-0.987894086	-16.70215048	15.71425639	200(203)
Niue	Parkinson's disease	0.72622205	-5.874281471	-18.4058367	12.53155523	107(90)
North Macedonia	Parkinson's disease	0.750629703	-9.93603899	-18.9695411	9.033502107	32(14)
Northern Mariana Islands	Parkinson's disease	0.771535213	-5.914853906	-18.77491246	12.86005855	116(88)
Norway	Parkinson's disease	0.91613281	-5.022152418	-18.97258875	13.95043633	146(110)
Oman	Parkinson's disease	0.773391602	-3.974715126	-18.82862018	14.85390505	175(138)
Pakistan	Parkinson's disease	0.504028689	-6.369512493	-16.69855282	10.32904032	54(77)
Palau	Parkinson's disease	0.754046931	-4.697366724	-18.77608073	14.078714	152(119)
Palestine	Parkinson's disease	0.631011665	-8.03447299	-16.7031224	8.668649409	28(37)
Panama	Parkinson's disease	0.708864828	-2.824846294	-18.42875451	15.60390821	198(166)
Papua New Guinea	Parkinson's disease	0.417797443	-4.232160536	-16.5366748	12.30451431	100(131)

				5		
Paraguay	Parkinson's disease	0.635718099	-8.279733102	-16.71259261	8.432859506	26(34)
Peru	Parkinson's disease	0.662054037	-4.469743451	-18.44466339	13.97491994	149(124)
Philippines	Parkinson's disease	0.651219329	-6.558552775	-18.37791421	11.81936144	87(71)
Poland	Parkinson's disease	0.812042809	-6.746969708	-18.60838564	11.86141594	88(67)
Portugal	Parkinson's disease	0.744151851	-3.759579498	-18.8266141	15.0670346	180(144)
Puerto Rico	Parkinson's disease	0.825525847	-4.068298156	-18.87987217	14.81157401	174(137)
Qatar	Parkinson's disease	0.846860584	-5.37148406	-18.95535719	13.58387313	136(102)
Republic of Korea	Parkinson's disease	0.886675267	-6.340578916	-18.98309386	12.64251494	110(78)
Republic of Moldova	Parkinson's disease	0.732214875	-4.878469316	-18.60579573	13.72732641	140(112)
Romania	Parkinson's disease	0.768453864	-5.513683114	-18.93414055	13.42045744	133(100)
Russian Federation	Parkinson's disease	0.808536005	-6.168856398	-18.60947217	12.44061577	104(83)
Rwanda	Parkinson's disease	0.435588706	-6.796676858	-16.5124359	9.715759044	40(65)
Saint Kitts and Nevis	Parkinson's disease	0.754987055	-4.549926546	-18.88337299	14.33344644	160(120)
Saint Lucia	Parkinson's disease	0.672509735	-3.875240709	-18.3067803	14.43153959	163(140)

Saint Vincent and the Grenadines	Parkinson's disease	0.637195963	-3.696349708	-16.7124454	13.0160957	121(148)
Samoa	Parkinson's disease	0.593392769	-6.771940284	-16.71158577	9.939645483	47(66)
San Marino	Parkinson's disease	0.888005474	-3.482818488	-18.96869735	15.48587886	196(152)
Sao Tome and Principe	Parkinson's disease	0.505413747	-1.386756352	-16.70497932	15.31822296	192(196)
Saudi Arabia	Parkinson's disease	0.815143493	-5.886009649	-18.93200922	13.04599957	122(89)
Senegal	Parkinson's disease	0.408054193	-1.764802603	-16.52405772	14.75925511	170(191)
Serbia	Parkinson's disease	0.792416294	-7.240914039	-18.899994	11.65907996	84(55)
Seychelles	Parkinson's disease	0.730150775	-11.39506806	-18.50224376	7.107175708	13(9)
Sierra Leone	Parkinson's disease	0.358665881	-1.776644926	-16.53474628	14.75810136	169(189)
Singapore	Parkinson's disease	0.856097766	-2.29098646	-18.95891726	16.6679308	204(181)
Slovakia	Parkinson's disease	0.81061053	-5.600081563	-18.68338798	13.08330642	123(98)
Slovenia	Parkinson's disease	0.842430731	-5.115142032	-18.95896933	13.84382729	143(108)
Solomon Islands	Parkinson's disease	0.429360316	-9.246567562	-16.53191661	7.285349049	15(20)
Somalia	Parkinson's disease	0.077688109	-2.066887066	-2.946264582	0.879377516	1(185)

South Africa	Parkinson's disease	0.679626598	-2.529283129	-18.44017628	15.91089315	202(174)
South Sudan	Parkinson's disease	0.278371125	-2.760486835	-10.89665637	8.136169535	24(168)
Spain	Parkinson's disease	0.769283698	-7.320684011	-18.97901937	11.65833536	83(53)
Sri Lanka	Parkinson's disease	0.701534935	-3.089963817	-18.44581764	15.35585383	194(160)
Sudan	Parkinson's disease	0.541949735	-4.28277055	-16.7105072	12.42773665	102(129)
Suriname	Parkinson's disease	0.633665739	-4.255293782	-16.71106708	12.4557733	105(130)
Sweden	Parkinson's disease	0.886880299	-6.940401733	-18.5824914	11.64208966	81(63)
Switzerland	Parkinson's disease	0.933059111	-7.187492989	-18.83337674	11.64588375	82(58)
Syrian Arab Republic	Parkinson's disease	0.623004075	-8.227844096	-16.70147423	8.473630138	27(35)
Taiwan (Province of China)	Parkinson's disease	0.874747053	-7.621404811	-18.6071633	10.98575849	66(46)
Tajikistan	Parkinson's disease	0.541511187	-4.74929468	-16.68217045	11.93287577	92(115)
Thailand	Parkinson's disease	0.682547933	-7.147892035	-18.26097909	11.11308705	73(60)
Timor-Leste	Parkinson's disease	0.444667619	-6.324764382	-16.53415195	10.20938757	53(79)
Togo	Parkinson's disease	0.408533695	-2.552585942	-16.51924815	13.96666221	148(173)
Tokelau	Parkinson's disease	0.686425621	-5.554853317	-18.4460277	12.89117439	117(99)

				1		
Tonga	Parkinson's disease	0.626349936	-7.918982786	-16.68400177	8.765018981	30(41)
Trinidad and Tobago	Parkinson's disease	0.768763254	-3.848333968	-18.98209654	15.13376257	182(142)
Tunisia	Parkinson's disease	0.682432216	-8.482654797	-18.44219948	9.959544685	48(31)
Turkmenistan	Parkinson's disease	0.682160776	-3.462170401	-18.44533646	14.98316606	177(153)
Tuvalu	Parkinson's disease	0.576620529	-7.374656631	-16.70256012	9.327903488	36(51)
Türkiye	Parkinson's disease	0.712692673	-7.088352077	-18.25681253	11.16846046	74(62)
Uganda	Parkinson's disease	0.423261181	-1.871283076	-16.46777964	14.59649656	167(188)
Ukraine	Parkinson's disease	0.760773913	-5.601891343	-18.79466842	13.19277708	128(97)
United Arab Emirates	Parkinson's disease	0.849317734	-4.72728607	-18.67650696	13.94922089	144(116)
United Kingdom	Parkinson's disease	0.859000182	-8.299260852	-18.98336549	10.68410464	62(33)
United Republic of Tanzania	Parkinson's disease	0.446568273	-3.706570866	-16.44685314	12.74028228	112(146)
United States of America	Parkinson's disease	0.862448354	-7.895646316	-18.9572888	11.06164248	71(44)
United States Virgin Islands	Parkinson's disease	0.821830853	-3.726256407	-18.97467326	15.24841685	185(145)

Uruguay	Parkinson's disease	0.719283445	-7.478116404	-18.44361043	10.96549402	64(47)
Uzbekistan	Parkinson's disease	0.662621694	-2.929875722	-18.44450449	15.51462877	197(162)
Vanuatu	Parkinson's disease	0.473100706	-5.285514217	-16.66403512	11.37852091	77(104)
Venezuela (Bolivarian Republic of)	Parkinson's disease	0.596513059	-3.521441088	-16.69203089	13.1705898	127(151)
Viet Nam	Parkinson's disease	0.627933721	-10.16720675	-16.68507156	6.517864813	10(12)
Yemen	Parkinson's disease	0.450376375	-7.192105803	-16.46657083	9.274465024	34(56)
Zambia	Parkinson's disease	0.505948954	-2.561907971	-16.67355447	14.11164649	153(172)
Zimbabwe	Parkinson's disease	0.473819486	-7.135381919	-16.63593463	9.500552715	37(61)

SUPPLEMENTARY FIGURES

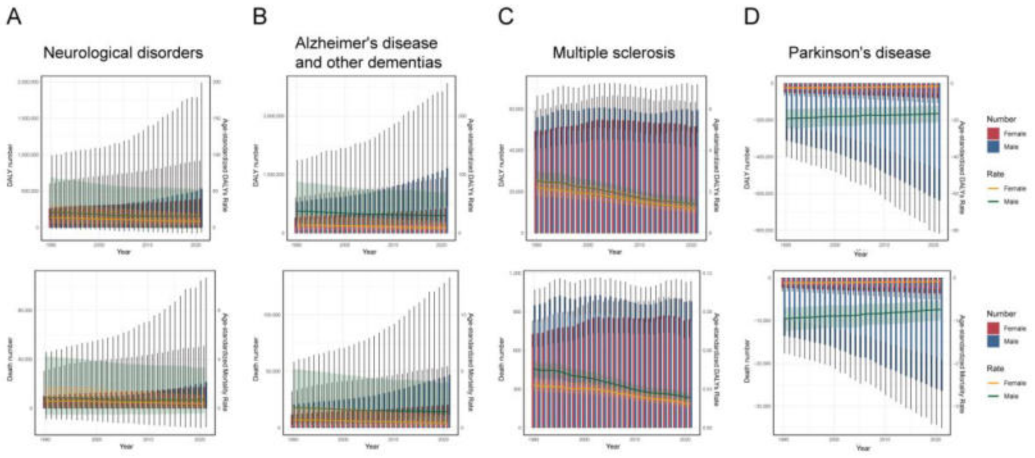

Supplementary Figure 1: Global DALYs burden of tobacco-related neurological disorders from 1990 to 2021.(A) The DALYs and ASDR for tobacco-related neurological disorders; (B) The DALYs and ASDR for tobacco-related Alzheimer’s disease and other dementias; (C) The DALYs and ASDR for tobacco-related Multiple sclerosis; (D) The DALYs and ASDR for tobacco-related Parkinson’s disease. DALYs, disability-adjusted life years; ASDR, age-standardized DALYs rate.

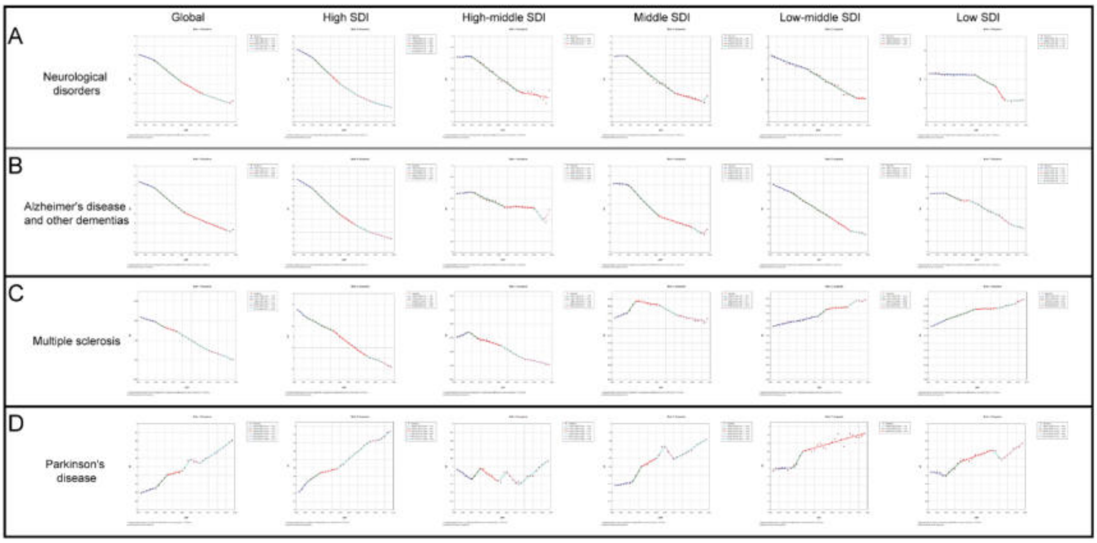

Supplementary Figure 2: Joinpoint regression analysis of ASDR in different regions from 1990 to 2021, the sequence of each line represents Global, High SDI, High-middle SDI, Middle SDI, Low-middle SDI, Low SDI. (A) ASDR for tobacco-related neurological disorders; (B) ASDR for tobacco-related Alzheimer's disease and other dementias; (C) ASDR for tobacco-related Multiple sclerosis; (D) ASDR for tobacco-related Parkinson's disease. SDI, socio-demographic index; ASDR, age-standardized DALYs rate.

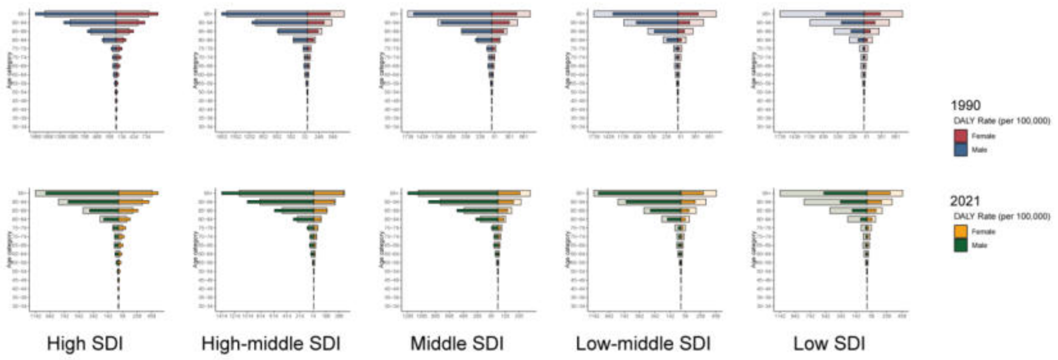

Supplementary Figure 3: Proportion of DALYs for tobacco-related neurological disorders in different sex groups, the sequence of each line represents Global, High SDI, High-middle SDI, Middle SDI, Low-middle SDI, Low SDI.

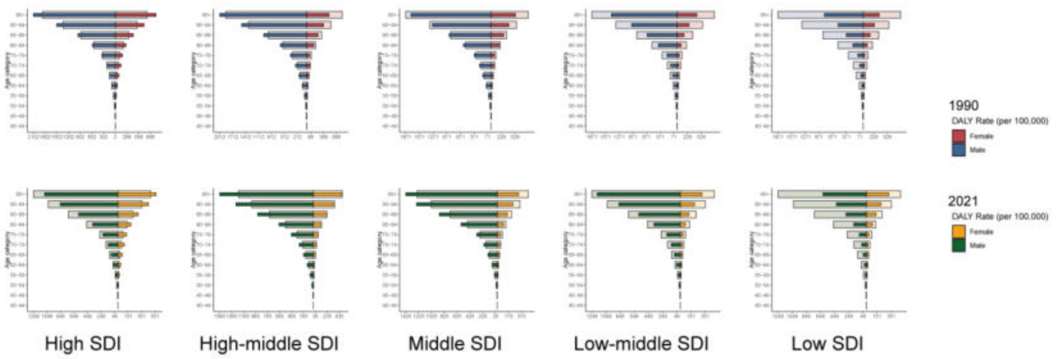

Supplementary Figure 4: Proportion of DALYs for tobacco-related Alzheimer’s disease and other dementias in different sex groups, the sequence of each line represents Global, High SDI, High-middle SDI, Middle SDI, Low-middle SDI, Low SDI.

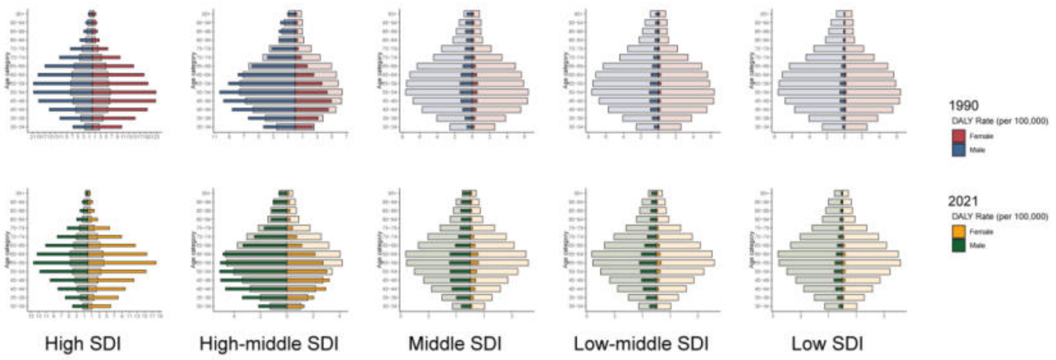

Supplementary Figure 5: Proportion of DALYs for tobacco-related Multiple sclerosis in different sex groups, the sequence of each line represents Global, High SDI, High-middle SDI, Middle SDI, Low-middle SDI, Low SDI.

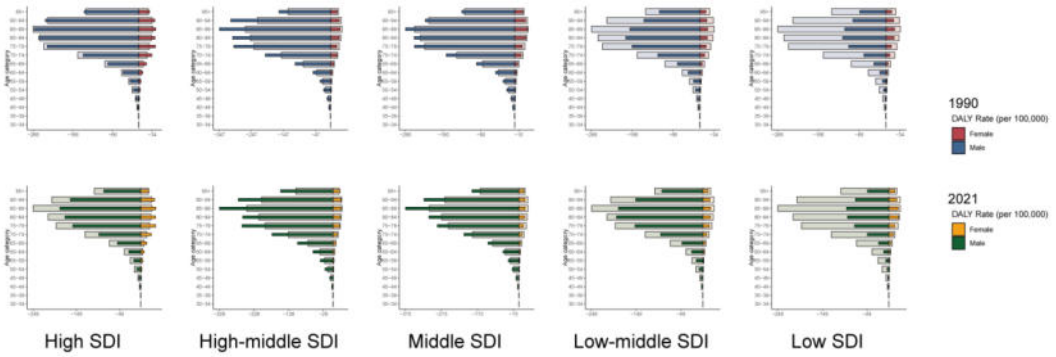

Supplementary Figure 6: Proportion of DALYs for tobacco-related Parkinson’s disease in different sex groups, the sequence of each line represents Global, High SDI, High-middle SDI, Middle SDI, Low-middle SDI, Low SDI.

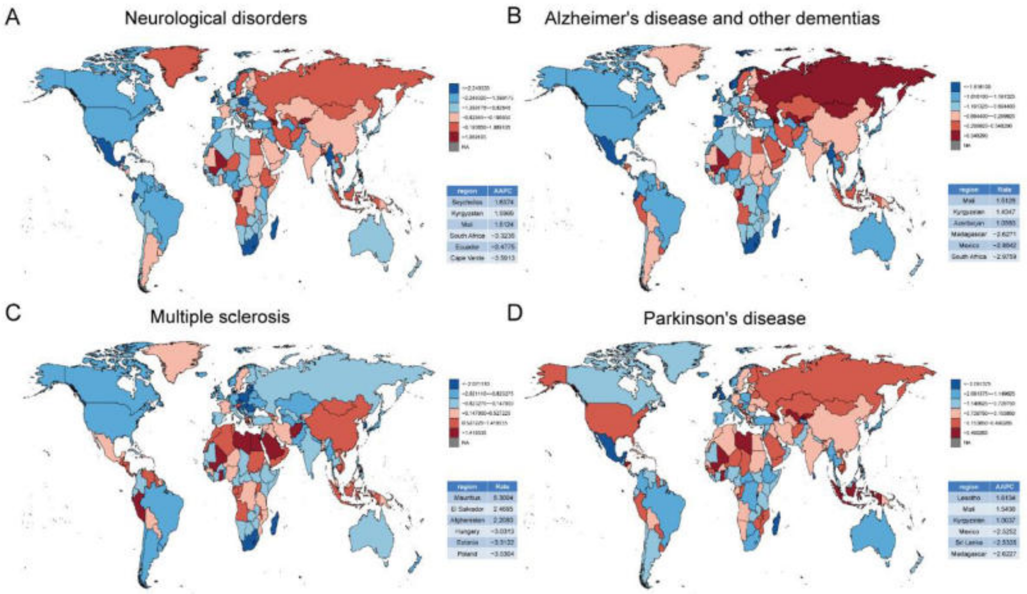

Supplementary Figure 7: AAPCs of ASDR in 204 countries worldwide. (A) AAPC for tobacco-related neurological disorders; (B) AAPC for tobacco-related Alzheimer's disease and other dementias; (C) AAPC for tobacco-related Multiple sclerosis; (D) AAPC for tobacco-related Parkinson's disease. AAPC, average annual percentage change; ASDR, age-standardized DALYs rate.

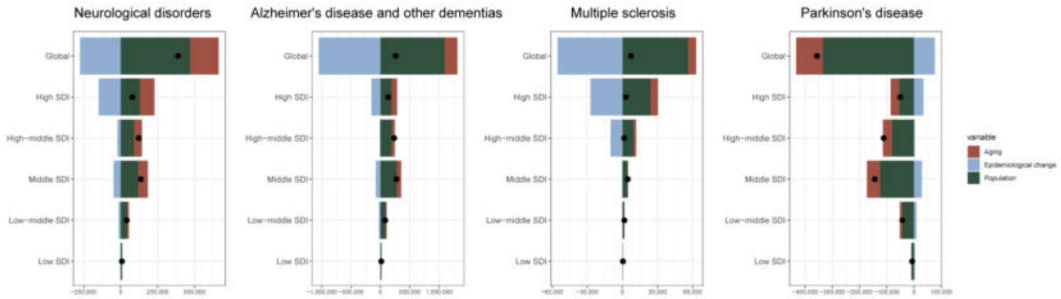

Supplementary Figure 8: Changes in DALYs of tobacco-related neurological disorders according to aging, population growth and epidemiological change from 1990 to 2021. DALYs, disability-adjusted life years.

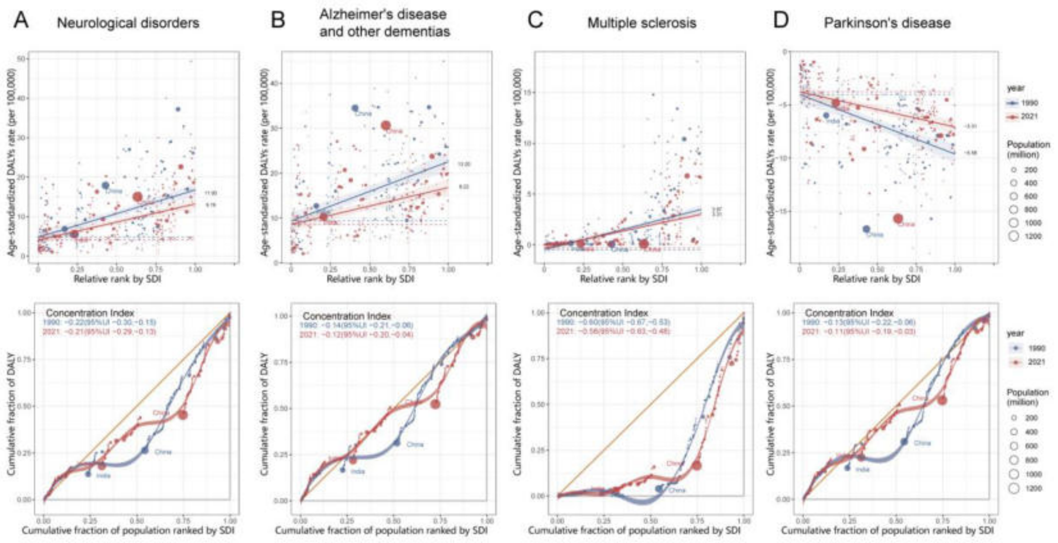

Supplementary Figure 9: Health Inequality Analysis of tobacco-related neurological disorders in ASDR from 1990 to 2021. (A) ASDR for tobacco-related neurological disorders; (B) ASDR for tobacco-related Alzheimer's disease and other dementias; (C) ASDR for tobacco-related Multiple sclerosis; (D) ASDR for tobacco-related Parkinson's disease. ASDR, age-standardized DALYs rate. ASDR, age-standardized DALYs rate.

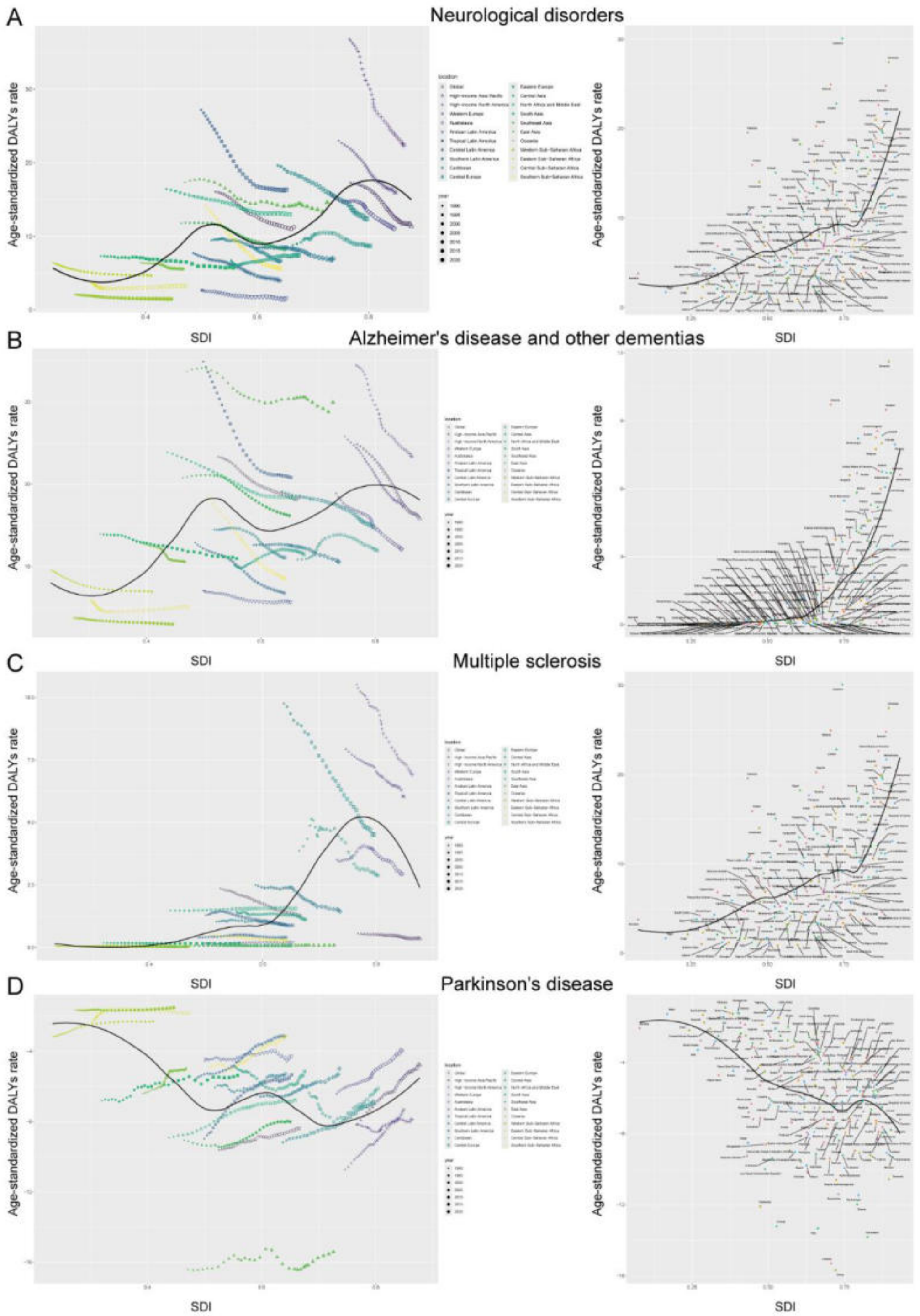

Supplementary Figure 10: Association between ASDR of and SDI. (A) ASDR for tobacco-related neurological disorders; (B) ASDR for tobacco-related Alzheimer's disease and other dementias; (C) ASDR for tobacco-related Multiple sclerosis; (D) ASDR for tobacco-related Parkinson's disease. SDI, socio-demographic index; ASDR, age-standardized DALYs rate.

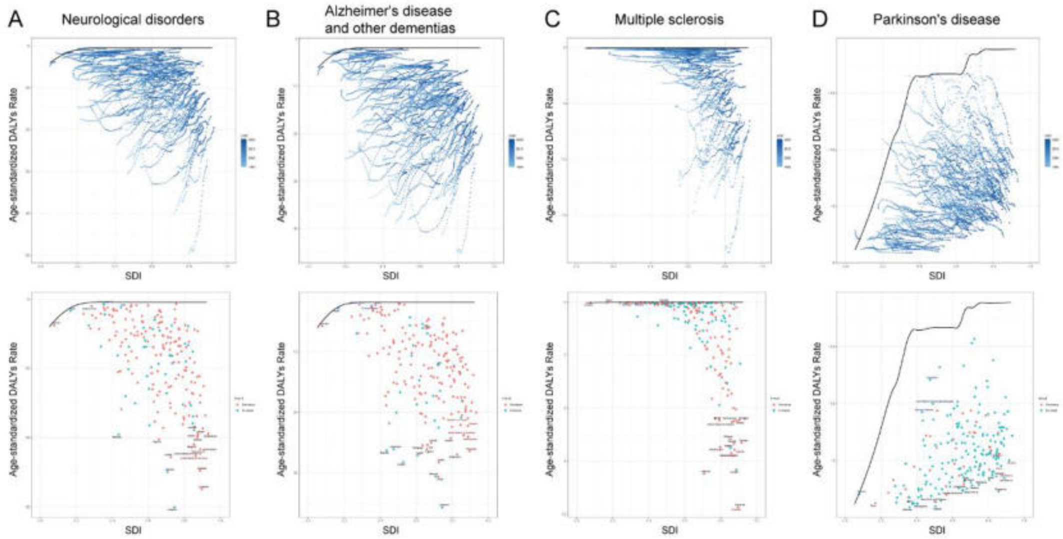

Supplementary Figure 11: Frontier analysis based on SDI and ASDR in 204 countries and territories. (A) ASDR for tobacco-related neurological disorders; (B) ASDR for tobacco-related Alzheimer's disease and other dementias; (C) ASDR for tobacco-related Multiple sclerosis; (D) ASDR for tobacco-related Parkinson's disease. ASDR, age-standardized DALYs rate.

© 2025 Zhang C. et al.
